# Radiomultiomics: quantitative CT clusters of severe asthma associated with multiomics

**DOI:** 10.1183/13993003.00207-2024

**Published:** 2024-11-21

**Authors:** Nazanin Zounemat Kermani, Kian Fan Chung, Giuseppe Macis, Giuseppe Santini, Franz A.A. Clemeno, Ali Versi, Kai Sun, Mahmoud I. Abdel-Aziz, Lars I. Andersson, Charles Auffray, Yusef Badi, Per Bakke, Chris Brightling, Paul Brinkman, Massimo Caruso, Pascal Chanez, Bertrand De Meulder, Ratko Djukanovic, Leonardo Fabbri, Stephen J. Fowler, Ildiko Horvath, Peter Howarth, Anna J. James, Johan Kolmert, Monica Kraft, Chuan-Xing Li, Anke H. Maitland-van der Zee, Mario Malerba, Alberto Papi, Klaus Rabe, Marek Sanak, Dominick E. Shaw, Dave Singh, Maria Sparreman Mikus, Maarten van Den Berge, Asa M. Wheelock, Craig E. Wheelock, Valentyna Yasinska, Yi-ke Guo, Scott Wagers, Peter J. Barnes, Andrew Bush, Peter J. Sterk, Sven-Erik Dahlen, Ian M. Adcock, Salman Siddiqui, Paolo Montuschi

**Affiliations:** 1 Data Science Institute, Imperial College London, London, UK; 2 National Heart and Lung Institute, Imperial College London, London, UK; 3 Royal Brompton and Harefield Hospitals, London, UK; 4 Radiology, Faculty of Medicine, Catholic University of the Sacred Heart, Rome, Italy; 5 Pharmacology, Faculty of Medicine, Catholic University of the Sacred Heart, Rome, Italy; 6 Department of Respiratory Sciences, University of Leicester, Leicester, UK; 7 Department of Pulmonology, Amsterdam UMC, University of Amsterdam, Amsterdam, The Netherlands; 8 Department of Medicine Huddinge, Karolinska Institutet, Stockholm, Sweden; 9 Department of Respiratory Medicine, Karolinska University Hospital, Stockholm, Sweden; 10 European Institute for Systems Biology and Medicine, CNRS-ENS-UCBL-INSERM, Université de Lyon, Lyon, France; 11 Department of Clinical Science, University of Bergen, Bergen, Norway; 12 Institute for Lung Health, NIHR Leicester Biomedical Research Centre, Department of Respiratory Sciences, University of Leicester, Leicester, UK; 13 Department of Biomedical and Biotechnological Sciences, University of Catania, Catania, Italy; 14 AP-HM – Clinique des Bronches, Allergies et Sommeil, Aix Marseille Université, Marseille, France; 15 NIHR Southampton Respiratory Biomedical Research Unit and Clinical and Experimental Sciences, University of Southampton, Southampton, UK; 16 Respiratory Medicine, Department of Translational Medicine, University of Ferrara, Ferrara, Italy; 17 Division of Infection, Immunity and Respiratory Medicine, School of Biological Sciences, University of Manchester, Manchester University NHS Foundation Trust, Manchester Academic Health Science Centre, Manchester, UK; 18 Semmelweis University, Budapest, Hungary; 19 Department of Women's and Children's Health, Karolinska Institutet, Stockholm, Sweden; 20 Institute of Environmental Medicine, Centre for Allergy Research, Karolinska Institutet, Stockholm, Sweden; 21 Samuel Bronfman Department of Medicine, Icahn School of Medicine at Mount Sinai Hospital, New York, NY, USA; 22 Department of Medicine Solna, Karolinska Institutet, Stockholm, Sweden; 23 Department of Translational Medicine, University of Piemonte Orientale, Novara, Italy; 24 LungenClinic Grosshansdorf and Department of Medicine, Christian Albrechts University, Airway Research Center North within the German Center for Lung Research (DZL), Kiel, Germany; 25 Division of Clinical Genetics and Molecular Biology, Department of Medicine, Jagiellonian University Medical College, Kraków, Poland; 26 Respiratory Research Unit, University of Nottingham, Nottingham, UK; 27 Centre for Respiratory Medicine and Allergy, Manchester University NHS Foundation Hospital Trust, University of Manchester, Manchester, UK; 28 Department of Pulmonology, University of Groningen, University Medical Center Groningen, Groningen, The Netherlands; 29 Biosci Consulting, Maasmechelen, Belgium; 30 I.M. Adcock, S. Siddiqui and P. Montuschi contributed equally to this article as lead authors and supervised the work

## Abstract

**Background:**

Lung quantitative computed tomography (qCT) severe asthma clusters have been reported, but their replication and underlying disease mechanisms are unknown. We identified and replicated qCT clusters of severe asthma in two independent asthma cohorts and determined their association with molecular pathways, using radiomultiomics, integrating qCT, multiomics and machine learning/artificial intelligence.

**Methods:**

We used consensus clustering on qCT measurements of airway and lung CT scans, performed in 105 severe asthmatic adults from the U-BIOPRED cohort. The same qCT measurements were used to replicate qCT clusters in a subsample of the ATLANTIS asthma cohort (n=97). We performed integrated enrichment analysis using blood, sputum, bronchial biopsies, bronchial brushings and nasal brushings transcriptomics and blood and sputum proteomics to characterise radiomultiomic-associated clusters (RACs).

**Results:**

qCT clusters and clinical features in U-BIOPRED were replicated in the matched ATLANTIS cohort. In the U-BIOPRED cohort, RAC1 (n=30) was predominantly female with elevated body mass index, mild airflow limitation, decreased CT lung volume and increased lung density and upregulation of the complement pathway. RAC2 (n=34) subjects had airway wall thickness and a mild degree of airflow limitation, with upregulation of proliferative pathways including neurotrophic receptor tyrosine kinase 2/tyrosine kinase receptor B, and downregulation of semaphorin pathways. RAC3 (n=41) showed increased lung attenuation area and air trapping, severe airflow limitation, hyperinflation, and upregulation of cytokine signalling and signalling by interleukin pathways, and matrix metallopeptidase 1, 2 and 9.

**Conclusions:**

U-BIOPRED severe asthma qCT clusters were replicated in a matched independent asthmatic cohort and associated with specific molecular pathways. Radiomultiomics might represent a novel strategy to identify new molecular pathways in asthma pathobiology.

## Shareable abstract

**Severe asthma qCT clusters are replicable and associated with selective molecular pathways. By clarifying the potential pathogenetic mechanisms underlying its heterogeneity, radiomultiomics might improve severe asthma personalised medicine.** https://bit.ly/3WfySPI

## Graphical abstract

**Figure:**
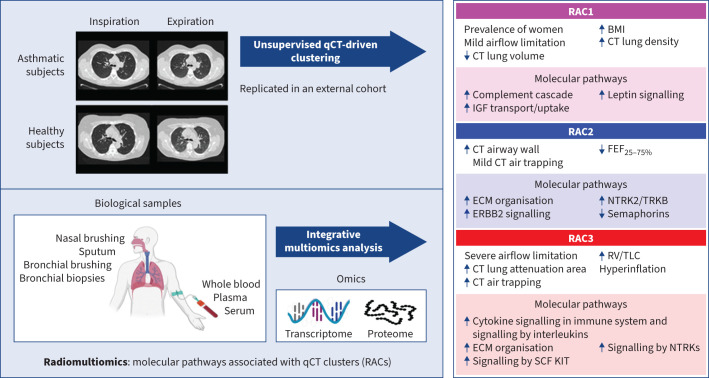
Overview of the study. (q)CT: (quantitative) computed tomography; RAC: radiomultiomic-associated cluster; BMI: body mass index; IGF: insulin-like growth factor; FEF_25–75%_: forced expiratory flow at 25–75% of forced vital capacity; ECM: extracellular matrix; RV: residual volume; TLC: total lung capacity. See the supplementary material for a full list of definitions of abbreviations of genes, proteins and pathways.

## Introduction

In asthma, quantitative high-resolution computed tomography (HRCT) (qCT) has revealed that bronchial wall thickening and/or air trapping is related to airflow obstruction, bronchodilator response [1, 2], asthma severity [3–5] and neutrophilic inflammation [6]. In addition, qCT identifies phenotypes of asthma derived from cluster analysis of clinical features [2, 7]. Furthermore, qCT phenotypes themselves are also characterised by varying degrees of luminal and airway wall changes and of air trapping reflecting asthma severity and degrees of airflow obstruction [8–10].

Here, we used qCT measurements from lung HRCT scans of the U-BIOPRED (Unbiased BIOmarker in Prediction of REspiratory Disease outcomes) asthma cohort [11] to identify qCT clusters and externally replicate them in the matched ATLANTIS (AssessmenT of smalL Airways involvemeNT In aSthma) cohort [12]. We further hypothesised that the clusters’ association to omics data from different airway compartments and blood would reveal pathways that are potentially associated with qCT clusters (radiomultiomic-associated clusters (RACs)). We refer to this approach as integrated radiomultiomics, based on a combination of qCT, transcriptomics and proteomics, and machine learning/artificial intelligence (AI). In an exploratory study, we linked these qCT clusters to molecular pathways derived from a transcriptomic and proteomic analysis obtained in patients from the U-BIOPRED study.

## Methods

### Participants

This proof-of-concept study used cross-sectional data from the multicentre pan-European U-BIOPRED study, including a baseline visit and a longitudinal visit 12–18 months apart [11]. The U-BIOPRED adult cohort (ClinicalTrials.gov: NCT01976767) included severe asthma non-smokers (n=311), severe asthma current or ex-smokers (>5 pack-years) (n=110), mild-to-moderate asthma subjects (n=88) and healthy control non-smoking participants (n=101) [11]. The flowchart of the study population selection process is shown in supplementary figure S1. Classification of asthma severity was based on international guidelines [13]. Severe and mild-to-moderate asthma participants were on maintenance treatment with inhaled corticosteroids quantified as fluticasone propionate equivalent at a daily dose of ≥500 and <500 μg·day^−1^, respectively [11]. All participant data were stored in the TranSMART data management system [14]. The U-BIOPRED study was approved by the ethics committee at each recruiting centre. Written informed consent was obtained from all participants.

### Quantitative HRCT

Lung HRCT was performed in 105 asthma participants in the U-BIOPRED study: 73 severe asthma non-smokers, 20 severe asthma current/ex-smokers, 12 mild-to-moderate asthma subjects and 15 healthy control participants (supplementary table S1). Volumetric whole-lung CT scans were obtained at total lung capacity (TLC) and residual volume (RV) following a standardised protocol [15]. Apollo software (VIDA Diagnostics, Coralville, IA, USA) was used for qCT [15]. Parameters reflecting 1) airway morphometry, including lumen area (LA), wall area (WA) and wall area as a percentage of total area (Pct WA), 2) emphysematous-like changes, including inspiratory percentage of low attenuation area with CT number frequency distribution < −950 HU (LAA −950 HU), mean lung density standard deviation of CT numbers (MLD SD) and the 15th percentile of the cumulative frequency distribution of CT numbers (percentile 15), and 3) air trapping, including expiratory percentage of low attenuation area with CT numbers < −856 HU (LAA −856 HU) were measured (supplementary table S2).

### Replication of qCT clusters

We performed internal replication of qCT clusters by splitting the U-BIOPRED dataset (n=105) into a training set (70% of the samples), which was used to build the model, and a testing set (30% of the samples), which was used for testing the model. Our external replication plan of the U-BIOPRED qCT clusters involved: 1) measuring compatibility between U-BIOPRED and ATLANTIS [12] demographic, physical–clinical and qCT variables, 2) matching cohorts for asthma severity and demographic characteristics, 3) using the same qCT measurements and machine learning workflow in both cohorts, and 4) examining whether U-BIOPRED and ATLANTIS qCT clusters were similar. More detailed methodology and a description of the analysis is provided in the supplementary material.

ATLANTIS is a multinational prospective asthma cohort of approximately 1000 individuals across nine countries (ClinicalTrials.gov: NCT01976767) [12]. We used the MatchIt R function [16] to identify an ATLANTIS subpopulation that matched U-BIOPRED. Individuals from the external replication cohort were selected to match patients in the U-BIOPRED cohort based on clinical and demographic features, including asthma severity. Individuals were not selected based on CT scan parameters. Therefore, ATLANTIS qCT clusters were used to replicate U-BIOPRED qCT clusters in similar populations. Although we could replicate qCT clusters, we could not replicate RACs as no corresponding omics datasets are available.

### Integration of qCT data and clustering

All analyses were performed in R version 4.1.2 (www.r-project.org) and Cytoscape 3.9.1 [17]. 50 qCT parameters were analysed (supplementary table S2). When two or more parameters were highly correlated (absolute Spearman ρ≥0.85), the most significant was selected.

Consensus clustering (R package ConsensusClusterPlus), applied to individuals with asthma only, was used to find the optimal number of RACs [18], using combined inspiratory and expiratory qCT data.

### Integrated functional analysis

We performed an integrated enrichment analysis to identify molecular phenotypes using transcriptomics (blood, sputum, bronchial brushings/bronchial biopsies and nasal brushings) and proteomics (plasma Human Protein Atlas antibody bead array, serum and sputum somaSCAN, and sputum LC-MSMS) datasets from U-BIOPRED [14]. First, we performed differential gene and protein expression analysis across and between the RACs (supplementary tables S3–S5).

p-values were used to prioritise genes/proteins by ActivePathways, utilising p-value merging to combine signals at the gene or protein level (evidence), followed by ranked hypergeometric tests to identify enriched pathways and processes [19]. p-values were merged row-wise using the Brown one-sided method [20] to obtain a single p-value for each gene across the various omics datasets. Pathway over-representation analysis was then conducted on the integrated gene list. Finally, by comparing gene lists of the identified pathways with the differentially expressed genes (DEGs) identified from each omics dataset, we determined the intersection between the pathway genes and the DEGs, providing supporting evidence for the identified pathways per omics dataset. p-values were adjusted by Benjamini–Hochberg false discovery rate. For each comparison and all omics, ActivePathways was run once with Reactome database version 84 [21].

We used Cytoscape [17] and the ActivePathways results are represented as enrichment maps, with nodes corresponding to pathways. Pathways with many shared genes are connected into networks. To further understand the overall expression of genes/proteins of the over-represented pathways across RACs, we created gene/protein signatures using the overlapped genes/proteins between omics data and pathways.

For each signature, we calculated the enrichment scores of the signature in the relevant omics datasets using gene set variation analysis (R package GSVA) [22]. We used the Kruskal–Wallis test for multiple comparisons and the Mann–Whitney U-test for pairwise comparisons of enrichment scores between any two RACs.

## Results

### Consensus clustering

Three RACs, RAC1 consisting of 30, RAC2 consisting of 34 and RAC3 consisting of 41 asthma participants (table 1 and supplementary table S6), were identified from consensus clustering (supplementary figure S2) of the selected 12 inspiratory and 10 expiratory lung HRCT parameters (supplementary table S7). Consensus clustering (supplementary figure S2a), the consensus matrix (supplementary figure S2b) and the k-cluster distribution (supplementary figure S2c) showed n=3 as the optimal cluster number, supported by the cumulative distribution of the k+1 cluster (supplementary figure S2d). The RAC stability was good, as shown by the correct iterative classification (supplementary figure S2b). Clusters were clearly separated, with a slight overlap between RAC2 and RAC3 (supplementary figure S2e).

**TABLE 1:** Representative characteristics of radiomultiomic-associated clusters (RACs)

	RAC1		RAC2		RAC3		p-value	RAC1 *versus* RAC2 p-value	RAC1 *versus* RAC3 p-value	RAC2 *versus* RAC3 p-value
		n		n		n				
**Participants, n**		30		34		41				
**Female, %**	77 (n=23)	30	56 (n=19)	34	41 (n=17)	41	**0.0128**	0.1145	**0.0039**	0.2508
**Age, years**	48.4±2.6	30	49.9±2.9	34	52.4±1.7	41	0.6033	0.6778	0.1870	0.3874
**BMI, kg·m^−2^**	36.8±1.4	30	27.9±0.8	34	27.8±0.8	41	**<0.0001**	**<0.0001**	**<0.0001**	0.9455
**Mild-to-moderate asthma subjects, n**	1	30	7	34	4	41	0.0874	0.0575	0.3881	0.2092
**Asthma exacerbations in previous 12 months, n**	2.5 (1.0–4.0)	30	2.0 (0–4.0)	34	1.0 (0–3.0)	41	0.0851	0.1997	**0.0237**	0.9180
**Pre-bronchodilator FEV_1_, % pred**	70.6 (59.6–90.1)	30	86.5 (71.9–98.1)	34	50.5 (38.5–78.2)	40	**<0.0001**	**0.0393**	**0.0106**	**<0.0001**
**Pre-bronchodilator FVC, % pred**	86.0±3.8	30	99.8±2.8	34	84.5±3.2	40	**0.0017**	**0.0042**	0.7561	**0.0006**
**Pre-bronchodilator FEV_1_/FVC, %**	70.8±2.6	30	70.4±1.5	34	53.9±1.9	40	**<0.0001**	0.8769	**<0.0001**	**<0.0001**
**Pre-bronchodilator FEF_25–75%_, L·s^−1^**	1.3 (0.8–2.1)	30	1.7 (0.9–2.5)	34	0.7 (0.4–1.0)	40	**0.0001**	0.2705	**0.0093**	**<0.001**
**Pre-bronchodilator FEF_25–75%_, % pred**	37.6 (22.7–57.9)	30	46.0 (27.5–65.5)	34	19.1 (13.1–37.8)	40	**<0.0001**	0.3366	**0.0069**	**<0.0001**
**Pre-bronchodilator PEF, % pred**	82.1 (64.4–92.2)	30	87.7 (74.3–103.3)	32	58.9 (42.0–80.1)	38	**<0.0001**	0.0974	**0.0087**	**<0.0001**
**Change in FEV_1_, post-salbutamol, %**	12.6 (3.0–29.8)	30	10.3 (4.2–13.8)	34	19.1 (6.6–27.1)	40	**0.0181**	0.2356	0.3138	**0.0022**
**RV, L**	2.0 (1.4–2.6)	23	2.1 (1.5–2.5)	26	2.8 (2.5–3.5)	34	**<0.0001**	0.6230	**0.0002**	**<0.0001**
**TLC, L**	5.0 (4.7–5.6)	23	6.0 (5.0–7.0)	26	7.3 (5.9–7.9)	34	**<0.0001**	**0.0019**	**<0.0001**	**0.0040**
**RV/TLC**	0.39±0.03	23	0.35±0.02	26	0.44±0.02	34	**0.0191**	0.2775	0.1412	**0.0035**
**s*G*_aw_, kPa^−1^·s^−1^**	0.6 (0.2–1.8)	21	1.1 (0.7–1.8)	23	0.6 (0.3–0.8)	34	**0.0104**	0.1654	0.3284	**0.0012**
**ACQ-5, average score**	2.4±0.2	29	1.7±0.2	30	2.3±0.2	37	0.0604	**0.0195**	0.6292	0.0669
**Current/ex-smokers/non-smokers, n**	0/9/21	30	2/7/25	34	5/11/25	41	0.2913	0.3077	0.1396	0.4643
**Pack-years, n**	10.0 (1.7–15.3)	9	5.2 (4.0–23.0)	9	10.0 (3.0–22.5)	15	0.7241	0.4885	0.5483	0.8264
***F*_ENO_, ppb**	26.0 (14.5–79.5)	25	32.3 (18.1–50.4)	34	30.0 (19.0–59.0)	39	0.9756	0.9787	0.8350	0.8755
**Blood eosinophils, cells·μL^−1^**	180 (100–300)	29	200 (100–500)	33	300 (180–500)	41	0.0781	0.1308	**0.0313**	0.3738
**Blood neutrophils, cells·μL^−1^**	4700 (3600–7050)	29	4200 (3050–6445)	33	4690 (3295–7600)	41	0.5306	0.2419	0.5396	0.6323
**Sputum eosinophils counts, %^#^**	4.4 (0.7–20.0)	12	2.1 (0.8–9.5)	18	6.0 (0.4–32.9)	20	0.8398	0.7230	0.9009	0.5775
**Sputum neutrophil counts, %^#^**	44.9±4.8	12	53.6±5.6	18	58.0±6.4	20	0.3506	0.2811	0.1626	0.6090

Data for continuous variables are presented as mean±sem or median (interquartile range); categorical variables are expressed as numbers or percentages. BMI: body mass index; FEV_1_: forced expiratory volume in 1 s; FVC: forced vital capacity; FEF_25–75%_: forced expiratory flow at 25–75% of FVC; PEF: peak expiratory flow; RV: residual volume; TLC: total lung capacity; s*G*_aw_: specific airway conductance; ACQ: Asthma Control Questionnaire; *F*_ENO_: fractional exhaled nitric oxide. ^#^: sputum cell counts are expressed as a percentage of non-squamous cells. Normality of variables was tested using the D'Agostino–Pearson omnibus normality test. Between-group comparisons were performed using ANOVA with Tukey's multiple comparison test for normally distributed data or the Kruskal–Wallis test with Dunn's multiple comparison test for non-parametric data. A Chi-squared test was used for multiple comparisons of categorical variables. If the Chi-squared test was positive, Fisher's exact test was used for pairwise comparisons of categorical variables. Bold indicates significant p-values (p<0.05).

**TABLE 2:** Values of representative high-resolution computed tomography (CT) parameters used for clustering across radiomultiomic-associated clusters (RACs) and the healthy control subject (HS) group^#^

	RAC1		RAC2		RAC3		HS		p-value	RAC1 *versus* RAC2 p-value	RAC1 *versus* RAC3 p-value	RAC2 *versus* RAC3 p-value	RAC1 *versus* HS p-value	RAC2 *versus* HS p-value	RAC3 *versus* HS p-value
		n		n		n		n							
**Participants, n**		30		34		41		15							
**Inspiratory BSA (Mosteller formula)**	2.12 (1.96–2.31)	30	1.98 (1.80–2.05)	34	1.94 (1.84–2.07)	41	2.01 (1.79–2.08)	15	**0.0047**	**<0.01**	**<0.05**	0.6547	**0.0200**	0.8427	0.9089
**Inspiratory adjusted CT lung volume, mm^3^**	4271 (3760–4842)	29	5553 (4990–6345)	34	6556 (5591–7448)	41	5239 (4613–6957)	15	**<0.0001**	**<0.001**	**<0.0001**	**0.0028**	**<0.01**	0.9464	0.0805
**Inspiratory LAA −950 HU, %**	5.7 (3.6–9.5)	29	8.2 (6.0–10.4)	34	13.8 (10.3–17.9)	41	7.2 (3.8–12.8)	15	**<0.0001**	**0.0384**	**<0.0001**	**<0.001**	0.3500	0.6665	**<0.01**
**Inspiratory LA median, mm^2^**	16.7±0.8	29	21.7±0.8	30	18.0±0.7	41	20.1±1.4	14	**0.0001**	**<0.001**	0.1938	**<0.01**	**<0.05**	0.5161	0.0657
**Inspiratory percentile 15, HU**	−914.9±4.4	29	−934.6±2.1	34	−947.8±1.8	41	−929.9±4.8	15	**<0.0001**	**<0.0001**	**<0.0001**	**<0.01**	**<0.05**	0.3050	**<0.01**
**Inspiratory WA mean BSA, mm^2^·m^−2^**	14.1±0.4	29	18.9±0.5	30	15.7±0.4	41	16.6±0.8	14	**<0.0001**	**<0.0001**	0.2800	**<0.0001**	**<0.05**	**<0.05**	0.1642
**Expiratory LAA −856 HU, %**	9.8±1.4	28	12.5±1.7	33	31.8±2.6	38	6.3±1.4	15	**<0.0001**	0.4137	**<0.0001**	**<0.0001**	0.1102	**0.0268**	**<0.0001**
**Expiratory WA mean BSA, mm^2^·m^−2^**	13.9 (11.8–17.5)	18	18.6 (15.3–19.1)	27	12.9 (11.8–15.7)	34	14.0 (13.1–15.4)	14	**<0.0001**	**<0.05**	0.4615	**<0.0001**	0.6611	**0.0032**	0.1943

Data for continuous variables are presented as mean±sem or median (interquartile range). BSA: body surface area; adjusted CT lung volume: CT lung volume adjusted for the phantom density rods; LAA −950 HU: percentage of low attenuation area with CT number < −950 HU; LA median: lumen area average of median values of right and left lung segmental bronchi; percentile 15: 15th percentile of the cumulative frequency distribution of CT numbers; WA mean BSA: wall area average of mean values of right and left lung segmental bronchi standardised by BSA; LAA −856 HU: percentage of low attenuation area with CT number < −856 HU. ^#^: only individuals with asthma were included in the cluster analysis (data from HS are shown as a control group). Normality of variables was tested using the D'Agostino and Pearson omnibus normality test. Between-group comparisons were performed using ANOVA with Tukey's multiple comparison test for normally distributed data or the Kruskal–Wallis test with Dunn's multiple comparison test for non-parametric data. Bold indicates significant p-values (p<0.05).

### Quantitative HRCT

**FIGURE 1:**
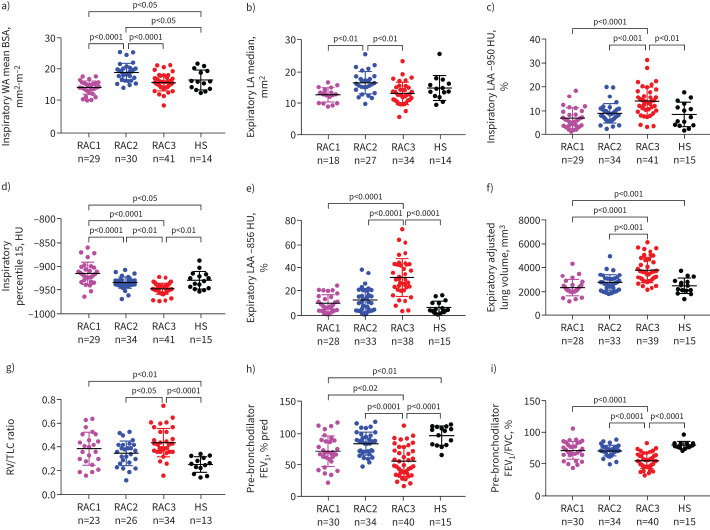
Scatter dot plots of selected quantitative computed tomography (qCT) parameters and pulmonary function tests across radiomultiomic-associated clusters (RACs) and healthy control subjects (HS). a) Inspiratory wall area average of mean values of right and left lung segmental bronchi standardised by body surface area (WA mean BSA). b) Expiratory lumen area average of median values of right and left lung segmental bronchi (LA median). c) Inspiratory percentage of low attenuation area with CT numbers < −950 HU (LAA −950 HU). d) Inspiratory 15th percentile of the cumulative frequency distribution of CT numbers (percentile 15). e) Expiratory percentage of low attenuation area with CT numbers < −856 HU (LAA −856 HU). f) Expiratory CT lung volume adjusted for the phantom density rods (adjusted CT lung volume). g) Residual volume/total lung capacity (RV/TLC) ratio. h) Pre-bronchodilator forced expiratory volume in 1 s (FEV_1_) percentage predicted. i) Pre-bronchodilator FEV_1_/forced vital capacity (FVC) ratio (percentage). Mean±sd or median with interquartile range values are shown depending on data distribution (see tables 1 and 2 and supplementary tables S6 and S7).

Supplementary table S8 shows values of all the radiological variables measured across asthma groups of different severity and healthy individuals. Table 2 shows representative qCT biomarkers across RACs and healthy control subjects. RAC2 patients had the highest qCT values reflecting airway wall thickness, including inspiratory WA average of mean values of right and left lung segmental bronchi (WA mean) (p<0.01 *versus* RAC1; p<0.001 *versus* RAC3) (supplementary table S7), and inspiratory (p<0.0001 *versus* either RAC1 or RAC3) (figure 1a and supplementary table S7) and expiratory WA standardised by body surface area (WA mean BSA) (p<0.05 *versus* RAC1; p<0.0001 *versus* RAC3) (table 2 and supplementary table S7). Compared with other RACs, RAC2 individuals also showed increased airway lumen area, including inspiratory (p<0.001 *versus* RAC1; p<0.01 *versus* RAC3) (table 2 and supplementary table S7) and expiratory LA average of median values of right and left lung segmental bronchi (LA median) (p<0.01 *versus* RAC1; p<0.01 *versus* RAC3) (figure 1b and supplementary table S7), and inspiratory (p<0.0001 *versus* RAC1; p<0.05 *versus* RAC3) (supplementary table S7) and expiratory LA average of mean values of right and left lung segmental bronchi standardised by BSA (LA mean BSA) (p<0.01 *versus* RAC1; p<0.01 *versus* RAC3) (supplementary table S7), all suggestive of predominant airway remodelling.

By contrast, RAC3 was characterised by predominant emphysematous-like changes as shown by the highest inspiratory LAA −950 HU (p<0.0001 *versus* RAC1; p<0.001 *versus* RAC2) (table 2 and figure 1c) and the lowest inspiratory percentile 15 (p<0.0001 *versus* RAC1; p<0.01 *versus* RAC2) (table 2 and figure 1d), and air trapping as reflected by the highest expiratory LAA −856 HU (p<0.0001 *versus* either RAC1 or RAC2) (table 2 and figure 1e). Inspiratory LAA −856 HU (p<0.0001 *versus* RAC1; p<0.001 *versus* RAC2) (supplementary table S7) and expiratory CT lung volume adjusted for the phantom density rods (p<0.0001 *versus* RAC1; p<0.001 *versus* RAC2) (figure 1f and supplementary table S7) were also elevated in RAC3, whereas lung density was reduced, as reflected by the lowest inspiratory (p<0.0001 *versus* RAC1; p<0.05 *versus* RAC2) (supplementary table S7) and expiratory MLD SD (p<0.0001 *versus* RAC1; p<0.01 *versus* RAC2) (supplementary table S7). All radiological values measured across all RACs are shown in supplementary table S7.

RAC1 individuals had no evidence of emphysematous-like changes or air trapping as reflected by inspiratory LAA −950 HU and expiratory LAA −856 HU values, respectively, similar to healthy individuals. In contrast, inspiratory adjusted CT lung volume (p<0.0003), reflecting lung volume, and inspiratory LA mean BSA (p<0.0004) and inspiratory LA median (p<0.02), reflecting lumen area, were reduced, while inspiratory (p<0.0001) and expiratory MLD SD (p<0.02), reflecting lung density, and Pct WA, reflecting airway wall thickness, were increased in RAC1 (supplementary table S9). Compared with healthy control subjects (supplementary tables S9–S11), airway wall thickness was increased in RAC2, as reflected by inspiratory WA mean (p<0.04) and inspiratory (p<0.02) and expiratory WA mean BSA (p<0.004) (supplementary table S10), but not in RAC1 (supplementary table S9) or RAC3 (supplementary table S11). In contrast, compared with healthy subjects, emphysematous-like changes, as reflected by inspiratory LAA −950 HU (p<0.002) and inspiratory percentile 15 (p<0.0001) were elevated in RAC3 (supplementary table S11), but not in RAC1 (supplementary table S9) or RAC2 (supplementary table S10). Compared with healthy control subjects, air trapping, as reflected by expiratory LAA −856 HU, was increased in RAC2 (p<0.02) (supplementary table S10) and, to a greater extent, in RAC3 (p<0.0001) (supplementary table S11). RAC1 showed reduced lung volume, as reflected by inspiratory adjusted CT lung volume (p<0.0003), and increased lung density, as reflected by inspiratory (p<0.0001) and expiratory MLD SD (p<0.02) (supplementary table S9). Based on asthma severity, there were no differences in qCT measurements except expiratory LAA −856 HU, elevated in severe asthma individuals, and inspiratory LA mean BSA, elevated in mild-to-moderate asthma individuals (supplementary table S8).

### Clinical features of RAC phenotypes

No differences in scores for clinical questionnaires, including Asthma Control Questionnaire (ACQ)-5, Asthma Quality of Life Questionnaire, Hospital Anxiety and Depression Scale, and Sino-Nasal Outcomes Test-20, were observed across the RACs (table 1 and supplementary table S6). However, pairwise comparisons showed that mean ACQ-5 score was higher in RAC1 than in RAC2 (p<0.02) (table 1).

The percentage of mild-to-moderate asthma subjects within each RAC was similar (table 1). Demographic and clinical characteristics of individual RACs are shown in table 1. RAC1 was characterised by a higher prevalence of women (77%; RAC2=56%, RAC3=41%) (Chi-squared p=0.013) and obesity, as reflected by elevated body mass index (BMI) (36.8±1.4 kg·m^−2^) compared with RAC2 (p<0.0001) and RAC3 (p<0.0001) (table 1), and elevated serum high-sensitivity C-reactive protein concentrations (p=0.017 *versus* RAC2; p=0.002 *versus* RAC3) and serum interleukin-6 (p<0.003 *versus* RAC2; p<0.003 *versus* RAC3) (supplementary table S6). Other clinical variables were similar across clusters (table 1 and supplementary table S6). Blood eosinophil counts, fractional exhaled nitric oxide (*F*_ENO_) and serum total IgE concentrations, reflecting a type 2 (T2)-high phenotype, were similar across RACs. Except serum interleukin-6, variables reflecting a T2-low phenotype were similar across clusters (table 1 and supplementary table S6). However, pairwise comparisons showed that blood eosinophils were higher in RAC3 than in RAC1 (p<0.04) (table 1). The data suggest that airway remodelling mostly transcends the T2-high/low concept. Furthermore, all patients had some degree of reversibility, but this was significantly greater in patients in RAC3 compared to patients in RAC2 (p=0.003) (table 1). A Chi-squared analysis (p=0.29) suggests no strong association between smoking status and RACs in asthmatic subjects (supplementary table S12).

### Pulmonary function tests

RAC3 showed the highest degree of airflow limitation, as reflected by elevated RV/TLC ratio (p<0.05 *versus* RAC2) (figure 1g), reduced pre-bronchodilator forced expiratory volume in 1 s (FEV_1_) absolute (p<0.001 *versus* RAC2) (supplementary table S6) and percentage predicted values (p<0.02 *versus* RAC1; p<0.0001 *versus* RAC2) (table 1 and figure 1h), reduced pre-bronchodilator forced vital capacity (FVC) percentage predicted values (p<0.001 *versus* RAC2) (table 1), reduced FEV_1_/FVC (p<0.0001 *versus* both RAC1 and RAC2) (table 1 and figure 1i), reduced pre-bronchodilator forced expiratory flow at 25–75% of FVC (FEF_25–75%_) absolute (p<0.001 *versus* RAC1; p<0.001 *versus* RAC2) (table 1) and percentage predicted values (p<0.01 *versus* RAC1; p<0.0001 *versus* RAC2) (table 1) and reduced pre-bronchodilator peak expiratory flow (PEF) percentage predicted values (p<0.01 *versus* RAC1; p<0.0001 *versus* RAC2) (table 1). RAC3 also showed reduced specific airway conductance compared with RAC2 (p<0.002) (table 1) and the highest degree of hyperinflation as reflected by RV (p<0.0003 *versus* RAC1; p<0.0001 *versus* RAC2) (table 1). Compared with healthy individuals, RV/TLC ratio was elevated in RAC1 (p<0.01) and RAC3 (p<0.0001) (figure 1g), but not in RAC2 participants, whereas FEV_1_/FVC was reduced only in RAC3 participants (p<0.0001) (figure 1h). The bronchodilator reversibility test results showed greater increase in post-salbutamol FEV_1_ percentage changes over baseline in RAC3 compared with RAC2 (p<0.003) (table 1).

### qCT cluster replication

**FIGURE 2:**
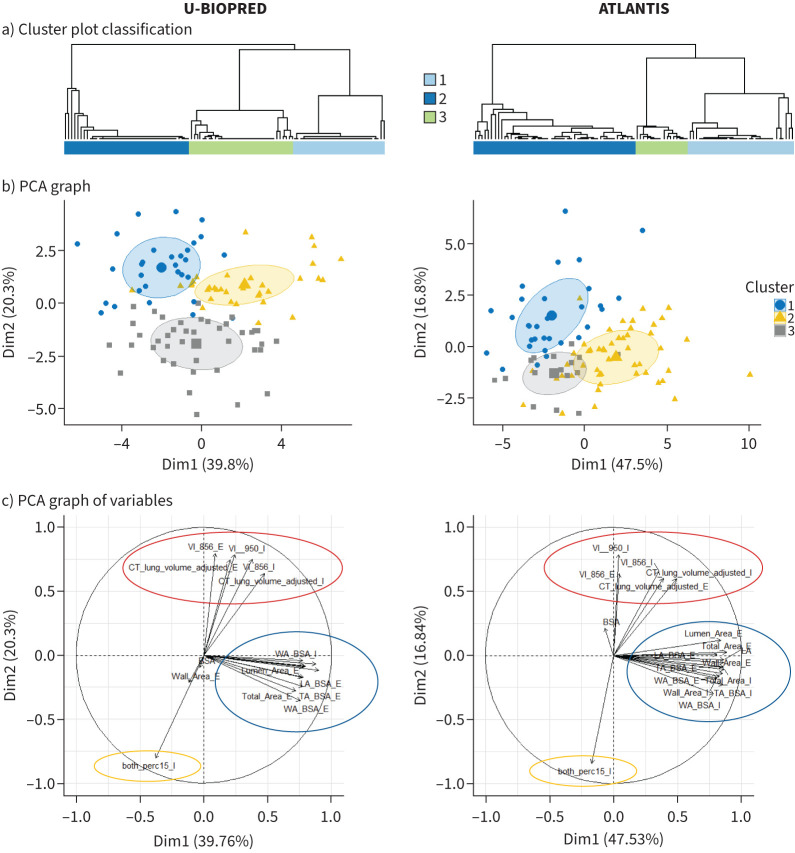
Comparative analysis of U-BIOPRED and ATLANTIS datasets across three analytical techniques. a) Clustering dendrograms of the consensus matrices for U-BIOPRED (left) and ATLANTIS (right) with the number of clusters k=3, illustrating the hierarchical clustering results and relationships between subjects based on their similarity. b) Principal component analysis (PCA) plots for U-BIOPRED (left) and ATLANTIS (right), where the first principal component (PC1) is plotted against the second principal component (PC2). Each dot represents a subject, coloured according to their membership in different quantitative computed tomography (qCT) clusters, highlighting the distribution and separation of subjects within the principal component space. c) Loadings of the used qCT features on PC1 and PC2 for U-BIOPRED (left) and ATLANTIS (right), showing the contribution and correlation of various qCT features to the principal components, aiding in understanding the underlying factors driving the clustering and separation observed in the subject-level PCA plots.

Based on 19 qCT biomarkers measured in both the discovery (supplementary table S7) and the matched ATLANTIS subset replication cohort (supplementary tables S13 and S14), three qCT clusters were identified in ATLANTIS (figure 2 and supplementary figure S3). Intra-cohort analysis, using our partial least square-linear discriminant analysis (PLS-LDA) classifier, indicated a strong performance within both the ATLANTIS and U-BIOPRED cohorts (table 3). In ATLANTIS, the classifier demonstrated a sensitivity of 0.88 (IQR 0.04), specificity of 0.94 (IQR 0.02), accuracy of 0.88 (IQR 0.05) and F1 score of 0.88 (IQR 0.04), while in U-BIOPRED, it achieved a sensitivity of 0.91 (IQR 0.08), specificity of 0.95 (IQR 0.03), accuracy of 0.91 (IQR 0.04) and F1 score of 0.90 (IQR 0.05). Moving to the inter-cohort analysis, where the classifier was tested across ATLANTIS–U-BIOPRED and U-BIOPRED–ATLANTIS cohorts, sensitivities ranged from 0.73 to 0.80, specificities from 0.85 to 0.89, accuracies from 0.72 to 0.79 and F1 scores from 0.73 to 0.78. In the combined analysis across both cohorts, the classifier achieved consistent performance with a sensitivity of 0.87 (IQR 0.06), specificity of 0.93 (IQR 0.02), accuracy of 0.88 (IQR 0.05) and F1 score of 0.87 (IQR 0.06) (table 3). These findings underscore the robustness and generalisability of our classifier across matched cohorts.

**TABLE 3:** Performance metrics of prediction of quantitative computed tomography (qCT) clusters using a partial least squares linear discriminant analysis classifier on the CT measurements dataset from two cohorts, ATLANTIS and U-BIOPRED, for intra-cohort, inter-cohort and combined analyses

Cohort	Sensitivity	Specificity	Accuracy	F1 score
**Intra-cohort**				
ATLANTIS	0.88 (0.04)	0.94 (0.02)	0.88 (0.05)	0.88 (0.04)
U-BIOPRED	0.91 (0.08)	0.95 (0.03)	0.91 (0.04)	0.90 (0.05)
**Inter-cohort**				
ATLANTIS–U-BIOPRED	0.73	0.85	0.72	0.73
U-BIOPRED–ATLANTIS	0.80	0.89	0.79	0.78
**Combined**	0.87 (0.06)	0.93 (0.02)	0.88 (0.05)	0.87 (0.06)

Interquartile range is presented in brackets.

The trend of qCT biomarkers across qCT clusters in U-BIOPRED (table 2 and supplementary table S7) and ATLANTIS (supplementary table S13) was similar, although absolute values were lower in ATLANTIS (supplementary table S14). In both cohorts, inspiratory WA mean BSA, inspiratory and expiratory LA mean BSA and LA median were elevated in qCT cluster 2; and inspiratory LAA −950 HU and expiratory LAA −856 HU were elevated in qCT cluster 3 (tables 2 and supplementary tables S7 and S13). The clinical characteristics of both cohorts showed elevated BMI in qCT cluster 1 and reduced FEV_1_/FVC%, FEF_25–75%_ absolute and percentage predicted values, and specific airway conductance in qCT cluster 3 (table 1 and supplementary tables S6 and S15).

### Molecular pathways of RACs

**FIGURE 3:**
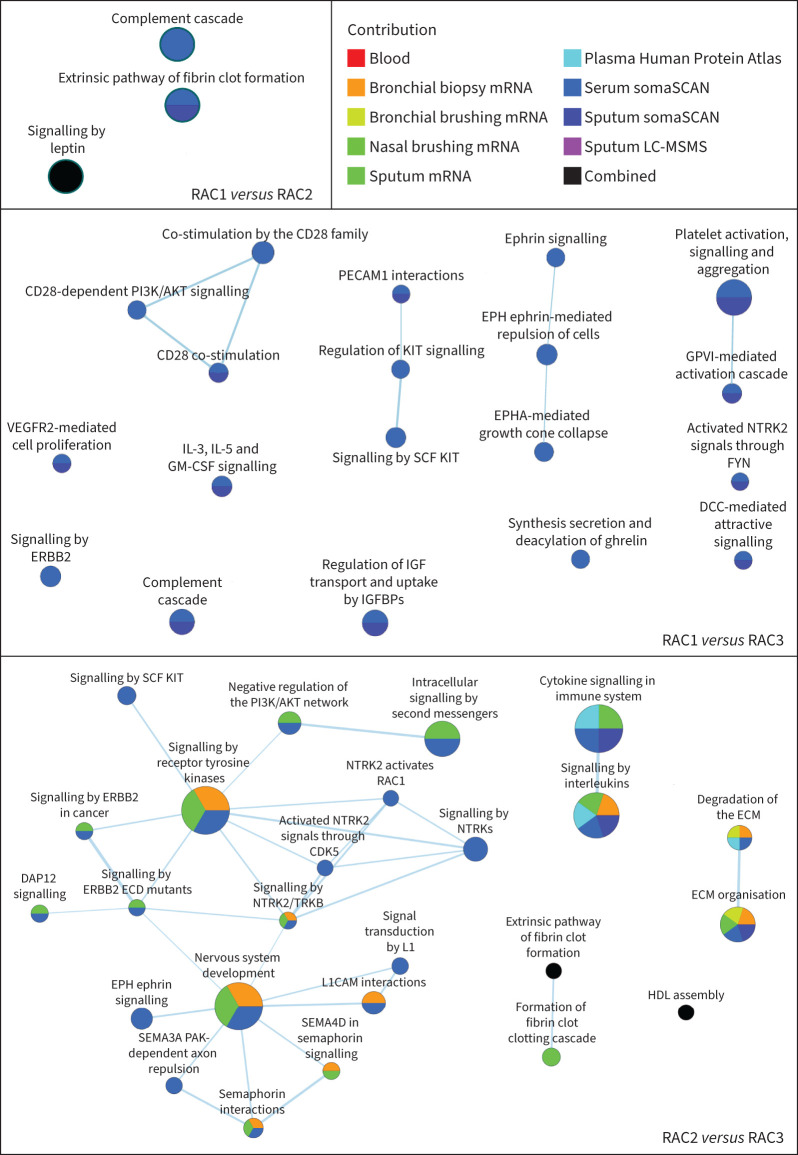
Enrichment maps for integrated pathway enrichment analysis in radiomultiomic-associated clusters (RACs). ActivePathways results are represented as enrichment maps, with nodes representing pathways and pathways with many shared genes connected into networks representing broader biological themes. Cytoscape software and the EnrichmentMap app were used to visualise integrated pathway enrichments as networks. Node colours correspond to the enriched pathways according to the supporting omics datasets, based on the scores matrix initially analysed in ActivePathways. See the supplementary material for a full list of definitions of abbreviations of genes, proteins and pathways.

Molecular pathways identified by differentially expressed proteins and genes were selectively associated with individual RACs (figure 3, table 4, supplementary table S16 and supplementary figure S4). Most of the differences in pathways were observed between RAC2 and RAC3 and between RAC1 and RAC3 (figure 3). Serum and sputum proteomics could detect all differences in pathway modulation between RAC1 and RAC3, whereas endotype differences between RAC2 and RAC3 were dependent upon both transcriptomic and proteomic signals from multiple compartments (figure 3).

**TABLE 4:** Overexpression of representative pathways/molecular phenotype expression across radiomultiomic-associated clusters (RACs)^#^

Pathway	Omic/biological matrix	RAC1 enrichment score	RAC2 enrichment score	RAC3 enrichment score	Kruskal–Wallis p-value	RAC1 *versus* RAC2 p-value	RAC1 *versus* RAC3 p-value	RAC2 *versus* RAC3 p-value	Overlapping genes or proteins
**Complement cascade RAC1 *versus* RAC2**	Proteomics/ serum	0.54 (0.24–0.67)	−0.05 (−0.35–0.23)	0.07 (−0.33–0.37)	**1.3e-05**	**1e-05**	**2e-05**	0.598561	CFH, CFI, C5, C9, C1S, CFD
**Complement cascade RAC1 *versus* RAC3**	Proteomics/ serum	0.48 (0.21–0.64)	0.09 (−0.19–0.35)	−0.08 (−0.36–0.32)	**0.00015**	**0.00113**	**5e-05**	0.27836	CFI, C9, CFH, C5, CD55, C3, FCN1, CRP
**Signalling by ERBB2 in cancer**	Proteomics/ serum	−0.16 (−0.32–0.07)	0.08 (−0.14–0.26)	−0.09 (−0.29–0.17)	0.05125	**0.02194**	0.74612	**0.049392**	SHC1, ERBB4, EGFR, ERBB2
**ECM organisation**	Transcriptomics/ bronchial biopsy	−0.31 (−0.36–0.15)	0.21 (−0.10–0.38)	−0.42 (−0.48–0.03)	**0.0184**	**0.01592**	0.28238	**0.03649**	DCN, ITGA6, LAMA4, DDR2, FBLN1, CTSB, LTBP2, DST, LTBP4
**GPVI-mediated activation cascade**	Proteomics/ serum	−0.56 (−0.72–0.10)	−0.35 (−0.66–0.03)	0.27 (−0.49–0.59)	**0.00146**	0.283067	**0.001364**	**0.00539**	PTPN6, LYN, FYN, PDPK1, CLEC1B, GP6
**Regulation of KIT signalling**	Proteomics/ serum	−0.53 (−0.78–0.03)	−0.33 (−0.58–0.09)	0.26 (−0.38–0.64)	**0.00494**	0.12495	**0.00268**	**0.04282**	PTPN6, LYN, FYN, SRC
**Regulation of IGF transport and uptake by IGFBPs**	Proteomics/ serum	0.43 (0.35–0.52)	−0.15 (−0.26–0.03)	−0.20 (−0.44–0.07)	**0.02478**	0.13333	**0.00586**	0.92308	C3, MFGE8, FN1, PAPPA, ALB, GPC3, SPARCL1, F2, FGG, MEP
**Degradation of the ECM**	Proteomics/ serum	−0.17 (−0.31–0.05)	−0.02 (−0.20–0.13)	0.08 (−0.19–0.30)	**0.01557**	0.10863	**0.00596**	0.13233	COL23A1, MMP2, BSG, CASP3, MMP12, KLK7, MMP17, ADAMTS5
**Signalling by interleukins**	Proteomics plasma	−0.25 (−0.46–0.22)	−0.22 (−0.33–0.06)	0.14 (−0.16–0.41)	**0.0038**	0.88862	**0.00886**	**0.0018**3	TSLP, NFKB2, NOS2, MMP9, MMP1, CCL11, IFNG, HSP90B1, ANXA2
**Signalling by SCF KIT**	Proteomics/ serum	−0.41 (−0.56–0.02)	−0.26 (−0.45–0.08)	0.07 (−0.33–0.50)	**0.00968**	0.35257	**0.00620**	**0.02438**	RAC1, PTPN11, TEC, PTPN6, FER, LYN, PRKCA, SRC, JAK2, LCK
**Signalling by NTRKs**	Proteomics/ serum	−0.32 (−0.42–0.24)	−0.36 (−0.52–0.12)	0.09 (−0.20–0.41)	**0.00109**	0.26896	**0.03042**	**0.00019**	RAC1, SHC1, PTPN11, RPS6KA3, ADCYAP1, CRK, MAPK1, DUSP3
**DAP1 signalling**	Proteomics/ serum	−0.34 (−0.65–0.18)	−0.47 (−0.68–0.22)	0.28 (−0.39–0.60)	**0.00131**	0.35257	**0.01193**	**0.00053**	RAC1, SHC1, BTK
**SEMA3A PAK-dependent axon repulsion**	Proteomics/ serum	−0.28 (−0.65–0.17)	−0.54 (−0.67–0.20)	0.27 (−0.22–0.53)	**0.00084**	0.24218	**0.01539**	**0.00026**	CFL1, RAC1
**Signalling by NTRK2/TRKB**	Proteomics/ serum	−0.21 (−0.62–0.15)	−0.47 (−0.68–0.29)	0.17 (−0.13–0.61)	**0.00106**	0.11975	**0.03245**	**3e-04**	RAC1, SHC1, PTPN11

Enrichment scores are presented as median (interquartile range). ^#^: the complete list of enrichment of molecular pathways across RACs is shown in supplementary table S16. See the supplementary material for a full list of definitions of abbreviations of genes, proteins and pathways. Datasets: Bronchial biopsy transcriptomics: RAC1, n=8; RAC2, n=13; RAC3, n=6. Sputum transcriptomics: RAC1, n=3; RAC2, n=5; RAC3, n=14. Plasma proteomics: RAC1, n=29; RAC2, n=33; RAC3, n=36. Serum proteomics: RAC1, n=27; RAC2, n=30; RAC3, n=41. Sputum proteomics: RAC1, n=4; RAC2, n=2; RAC3, n=11. Between-group comparisons were performed using the Kruskal–Wallis test with Dunn’s multiple comparison test. Bold indicates significant p-values (p<0.05).

**FIGURE 4:**
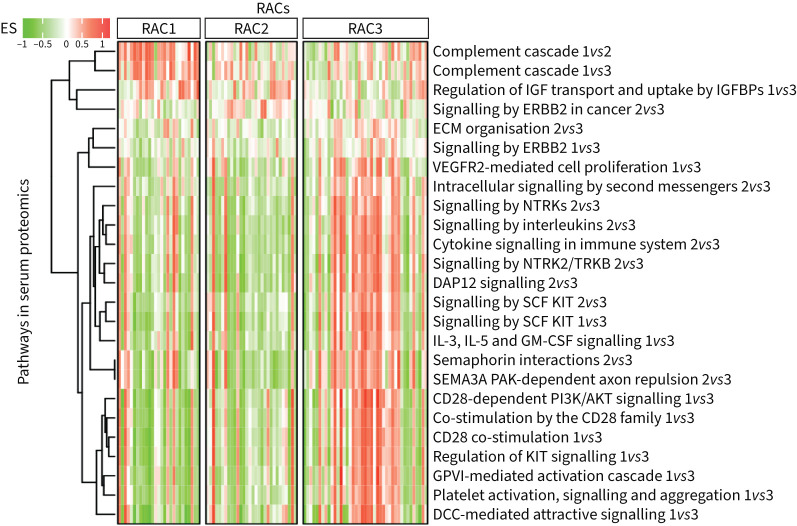
Modulation of serum proteomic pathways in severe asthma radiomultiomic-associated clusters (RACs). Enrichment scores (ESs) of 25 pathways (rows) are illustrated for subjects (columns) segmented by RACs. For each column, pathway names are shown on the right. Signatures and a list of proteins that represent a subset of pathways are shown in supplementary table S17. Serum proteins are measured by the somaSCAN proteomics and are differentially expressed across RACs (shown at the end of the pathway names for each comparison, *e.g.* 1*vs*2). See the supplementary material for a full list of definitions of abbreviations of genes, proteins and pathways.

**FIGURE 5:**
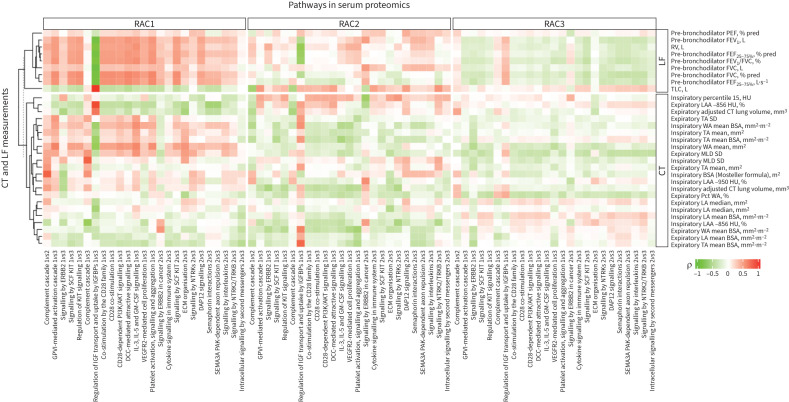
Heatmap showing the correlations between serum proteomics pathways and lung function (LF) tests and quantitative computed tomography (qCT) parameters in the three radiomultiomic-associated clusters (RACs). Positive correlations are shown in red; negative correlations are shown in green. See the supplementary material for a full list of definitions of abbreviations of genes, proteins and pathways. See the main text and supplementary table S2 for the definitions and details of the qCT parameters.

46 pathways were identified with adjusted p<0.05 (supplementary tables S3–S5). To visualise the pathway enrichment scores (table 4 and supplementary table S16) and molecular signatures across the RACs by omics datasets, we selected the serum proteomics dataset, because this is a readily accessible compartment and 25 over-represented pathways originated from this dataset (figure 4 and supplementary table S17). A heatmap shows correlations between CT parameters, lung function tests and enrichment scores of serum proteomics pathways (figure 5).

In bronchial biopsies, an extracellular matrix (ECM) organisation pathway, including fibulin-1 and latent transforming growth factor binding protein 2 (LTBP2) genes, functionally involved in tissue remodelling in respiratory diseases [23, 24], was upregulated in RAC2 compared with RAC1 (p<0.02) and RAC3 (p<0.04) (table 4, supplementary table S16 and supplementary figure S5a). RAC2 individuals also showed upregulation of the proliferative neurotrophic receptor tyrosine kinase 2/tyrosine kinase receptor B (NTRK2/TRKB) signalling, characterised by overexpression of the phosphatidylinositol-4,5-bisphosphate 3-kinase catalytic subunit α (PIK3CA) gene, using sputum transcriptomics, compared with RAC3 (p<0.003) (supplementary table S16 and supplementary figure S5b). Sputum proteomics also identified significant enrichment of ECM organisation (Kruskal–Wallis p<0.04) and interleukin molecular pathways (Kruskal–Wallis p<0.02), involving transforming growth factor (TGF)-β, in RAC2 individuals compared with RAC3 individuals (supplementary table S16 and supplementary figures S5c and d). In RAC2, sputum transcriptomics showed downregulation of the semaphorin interactions (p<0.04 *versus* RAC1; p<0.004 *versus* RAC3) and semaphorin 4D (SEMA4D) in semaphorin signalling (p<0.03 *versus* RAC3) pathways (supplementary table S16)

In serum proteomics, the proliferative signalling by Erb-B2 receptor tyrosine kinase 2 (ERBB2) in cancer molecular pathway, characterised by elevated serum SHC adaptor protein 1 (SHC1), Erb-B4 receptor tyrosine kinase 4 (ERBB4), epidermal growth factor receptor (EGFR) and ERBB2, was upregulated in RAC2 (p<0.03 *versus* RAC1; p<0.05 *versus* RAC3) (table 4, supplementary table S16 and supplementary figure S5e). The semaphorin 3A (SEMA3A) p21-activated kinase (PAK)-dependent axon repulsion (table 4, supplementary table S16 and supplementary figure S5f) and semaphorin interactions pathways (supplementary table S16) were downregulated in RAC2 compared with RAC3 (p<0.0003 for both). Cytokine signalling in the immune system (Kruskal–Wallis p<0.009) (supplementary table S16 and supplementary figure S5g) and signalling by interleukin (Kruskal–Wallis p<0.004) (table 4 and supplementary figure S5h) pathways, involving matrix metallopeptidase 1 (MMP1), MMP9 and nitric oxide synthase 2 (NOS2), and an ECM organisation pathway (Kruskal–Wallis p<0.04) (supplementary table S16 and supplementary figure S5i), involving MMP2, were selectively elevated in RAC3 as shown by plasma proteomics and serum proteomics data, respectively.

RAC3 showed elevated signalling by NTRKs (Kruskal–Wallis p<0.002) (table 4 and supplementary figure S6a), NTRK2/TRKB (Kruskal–Wallis p<0.002) (table 4 and supplementary figure S6b), interleukins (Kruskal–Wallis p<0.05) (supplementary table S16 and supplementary figure S6c) and intracellular second messengers (Kruskal–Wallis p<0.04) (supplementary table S16 and supplementary figure S6d), as reflected by increased serum levels of various proteins, including protein tyrosine phosphatase non-receptor type 11 (PTPN11), also known as SH2 domain-containing protein tyrosine phosphatase 2 (SHP2) (table 4 and supplementary table S16).

In RAC1, proteomics showed upregulation of two complement cascade pathways in serum (Kruskal–Wallis p<0.00002 and p<0.0002) (table 4 and supplementary figure S6e and f), elevated activity of regulation of insulin-like growth factor (IGF) transport and uptake by IGF binding proteins (IGFBPs) pathway in serum (Kruskal–Wallis p<0.03) (table 4 and supplementary figure S6g) and sputum (Kruskal–Wallis p<0.03) (supplementary table S16 and supplementary figure S6h), and elevated signalling by leptin (p<0.05) (supplementary table S3).

To summarise the results, we identified three qCT clusters in the severe asthma U-BIOPRED discovery cohort and replicated them in the matched ATLANTIS replication cohort. In the U-BIOPRED cohort, we found specific molecular pathways associated with individual qCT clusters, that we called RACs. However, we could not replicate these pathways because no asthma multiomic datasets with paired lung CT scan data are currently available.

## Discussion

In this study, we performed an initial qCT-based clustering that identified three clusters in the U-BIOPRED cohort, which was replicated in the matched ATLANTIS cohort. We then proceeded to associate these qCT clusters with proteomics and transcriptomics data. We identified associations with specific pathways that have helped us to understand the potential pathogenetic mechanisms underlying airway wall remodelling and air trapping in severe asthma. To the best of our knowledge, this is the first attempt to apply the combination of qCT, transcriptomics, proteomics analyses and machine learning/AI.

The three RACs were defined by differences in airway wall thickness and size, the degree of air trapping, and changes in lung density. RAC1 showed airway wall dimensions similar to healthy controls, but with reduced areas of low density and lower lung volumes. Compared with healthy subjects, RAC2 was characterised by increased airway wall thickness and mild air trapping, which was more pronounced in RAC3; only RAC3 showed the presence of large lungs and increasing lung areas of low density. The lung function indices showed that RAC1 had mild airflow obstruction, RAC2 mild reduction in peripheral airways function, and RAC3 the most severe degree of airflow obstruction and increased TLC and RV consistent with qCT biomarkers of air trapping changes that could underlie the severe airflow obstruction.Finally, RAC1 individuals were the most obese group.

We have externally replicated U-BIOPRED qCT clusters in a matched independent asthmatic cohort with a cross-replication balanced accuracy >0.80. Although qCT biomarker values in ATLANTIS were generally lower than those in U-BIOPRED, the trend across qCT clusters within each cohort was similar, with measures reflecting airway wall thickness and LA elevated in both qCT clusters 2 and those reflecting LAAs and air trapping elevated in both qCT clusters 3.

Compared with RAC3, RAC2 showed upregulation of an ECM organisation molecular pathway in bronchial biopsy involving fibulin-1 and LTBP2 genes, which contribute to airway remodelling [23, 24]. Fibulin-1 binding to LTBP2 activates TGF-β/Smad2 signalling [23, 24], a pivotal mechanism in airway remodelling [25]. RAC2 also showed upregulation of proliferative pathways, including signalling by NTRK2/TRKB, characterised by overexpression of the PIK3CA gene, and signalling by ERBB2 in cancer, as reflected by elevated serum concentrations of SHC1, ERBB4, EGFR and ERBB2, compared with RAC3. PIK3CA plays a pivotal role in human airway smooth muscle (ASM) cell proliferation [26, 27]. Our findings support a role for enhanced EGFR signalling in asthma airway remodelling [28, 29] and might suggest a functional involvement of ERBB2, also known as human epidermal growth factor receptor 2 (HER2), and ERBB4, also known as HER4, in its pathogenesis [30].

In contrast, serum proteomics indicated downregulation of the SEMA3A PAK-dependent axon repulsion pathway in RAC2. Likewise, sputum transcriptomics showed downregulation of the semaphorin interactions and SEMA4D in semaphorin signalling pathways. The data support the anti-remodelling effects of semaphorins reported in human ASM cells from asthma individuals *in vitro* [31] and in an *in vivo* mouse model of allergic asthma [32].

Serum proteomics in RAC3 showed upregulation of molecular pathways involving PTPN11, including cytokine signalling in the immune system, signalling by stem cell factor (SCF) KIT and signalling by NTRKs. PTPN11 has been shown to upregulate cigarette smoke-induced epithelial–mesenchymal transition by increasing MMP9 production in mouse lungs *in vivo* and in pulmonary epithelial cell cultures *in vitro* [33]. Serum proteomics also showed upregulation of cytokine signalling in the immune system and signalling by interleukin pathways involving MMP1, MMP9 and NOS2 in RAC3. These findings are consistent with elevated serum concentrations of MMPs in COPD individuals with emphysema [34] and with a role for NOS2 in mediating emphysematous remodelling [35].

The data highlight the importance of the pathways dysregulation, as subtle differences in pathway activation or molecular components may alter the overall functionality. As an example, the ECM organisation pathway characterised by elevated fibulin-1 and LTBP2 gene expression in bronchial biopsies was upregulated in RAC2, whereas the ECM organisation pathway, including collagen type XXIII α1 chain, collagen type IX α2 chain, syndecan 1 and tenascin C gene expression in sputum cells, was downregulated in RAC2. These data suggest different pathogenetic mechanisms underlying remodelling in the peripheral airways (RAC2) and in the lungs (RAC3). However, opposite regulation of individual pathways/molecular phenotypes in the same RAC might also be explained by different omics (transcriptomics, proteomics) and biological matrices used for analyses. The lack of correlation of the airway remodelling pathways with FEV_1_ can only be explained by an effect on ASM mass or on subepithelial fibrosis. We cannot check this but we would assume that there was a greater effect on subepithelial fibrosis rather than on ASM mass. The relationships between the modulation of selected ECM organisation pathways, inflammation and remodelling in RAC2 and RAC3 have to be clarified.

RAC1 was characterised by the presence of obesity, and upregulation of the complement pathway and leptin signalling. These findings are consistent with a 7-year longitudinal cohort study which has shown that activation of the complement system could be involved in the development of obesity [36]. Alternatively, the activation of the complement cascade might be a consequence of obesity [37].

The number of omics required to obtain the most complete information on molecular phenotype differences across RACs depends on the RAC pair comparisons. This supports the utility of radiomultiomics in deeply characterising severe asthma molecular endotypes. By combining structural and molecular information, radiomultiomics might provide a unique tool to explore the pathophysiological heterogeneity of severe asthma with the ultimate goal of predicting individual disease trajectories and therapeutic response.

Our study underscores the significance of inter-cohort generalisation and the advantages of combining datasets. Although CT measurements exhibited robust predictive capabilities within the distinct ATLANTIS and U-BIOPRED cohorts, cross-cohort assessments unveiled a marginal decline in performance. Nevertheless, through dataset amalgamation, we attained heightened predictive accuracy. This highlights the pivotal role of expansive and diverse datasets in refining predictive models for respiratory health outcomes. However, our study has some limitations, including the inability to determine a causative link between modulation of molecular phenotypes and airway remodelling and/or emphysematous-like changes, which requires future mechanistic studies and the use of one-sided rather than two-sided procedures for pooling p-values. In our study, the distribution of smokers/ex-smokers across RACs and their smoking exposure assessed by pack-years was similar, which would exclude a prominent role of smoking. However, further research is required to establish the potential effects of smoking on molecular pathways associated with RACs. HRCT longitudinal studies are required to determine the behaviour of RACs over time, their response to endotype-based pharmacotherapies, including anti-T2 therapies, and the effect of environmental exposure. The number of individuals with transcriptomic and proteomic data from all compartments studied (sputum, biopsies and blood) was also limited. These transcriptomic and proteomic molecular phenotypes characterising individual RACs require external replication in independent datasets from other severe asthma cohorts that have these corresponding parameters, but these do not currently exist.

In conclusion, we showed that qCT clusters can be replicated in an external matched severe asthma cohort and that they are associated with the modulation of specific molecular pathways. Including multiomics data and machine learning/AI into CT scan assessment might expand its diagnostic potential, while integrating CT scan data into multiomic studies might provide molecular pathway analysis with non-invasive structural correlates. This approach is of potential general application. By providing unique insights into the possible pathogenetic mechanisms associated with severe asthma heterogeneity, radiomultiomics might pave the way for a new approach to personalised medicine.

## Supplementary material

10.1183/13993003.00207-2024.Supp1 **Please note:** supplementary material is not edited by the Editorial Office, and is uploaded as it has been supplied by the author.
Supplementary material ERJ-00207-2024.Supplement
Supplementary table S3 ERJ-00207-2024.Table_S3
Supplementary table S4 ERJ-00207-2024.Table_S4
Supplementary table S5 ERJ-00207-2024.Table_S5

## Shareable PDF

10.1183/13993003.00207-2024.Shareable1 This PDF extract can be shared freely online.
Shareable PDF ERJ-00207-2024.Shareable
