# Supplementary material for: Radiomultiomics: quantitative CT clusters of severe asthma associated with multiomics
Source: Eur Respir J. 2024 Nov 21;64(5):2400207. doi: 10.1183/13993003.00207-2024 (PMC11579543; doi:10.1183/13993003.00207-2024)
Supplement: Supplementary file 1 [file ERJ-00207-2024.Supplement.pdf]

Supplementary information

Radiomultiomics: quantitative CT clusters of severe asthma associated with multi-omics

Nazanin Zounemat Kermani[1], Kian Fan Chung[2,3] Giuseppe Macis[4], Giuseppe Santini[5], Franz A. A. Clemeno[6], Ali Versi[1], Kai Sun[1], Mahmoud I. Abdel-Aziz[7], Lars I. Andersson[8,9], Charles Auffray[10], Yusef Badi[1], Per Bakke[11], Chris Brightling[12], Paul Brinkman[7], Massimo Caruso[13], Pascal Chanez[14], Bertrand De Meulder[10], Ratko Djukanovic[15], Leonardo Fabbri[16], Stephen J. Fowler[17], Ildiko Horvath[18], Peter Howarth[15], Anna J. James[19], Johan Kolmert[20], Monica Kraft[21], Chuan-Xing Li[22], Anke H. Maitland-van der Zee[7], Mario Malerba[23], Alberto Papi[17], Klaus Rabe[24], Marek Sanak[25], Dominick E. Shaw[26], Dave Singh[27], Maria Spearman Mikus[8], Maarten van Den Berge[28], Asa M. Wheelock[22], Craig E. Wheelock[20], Valentyna Yasinska[8,9], Yi-ke Guo[1], Scott Wagers[29], Peter J. Barnes[2], Andrew Bush[2], Peter J. Sterk[7], Sven-Erik Dahlen[8,9,20], *Ian M. Adcock[2], Salman Siddiqui[2] and *Paolo Montuschi[2,5], on behalf of the U-BIOPRED and ATLANTIS Study Groups

[1]Data Science Institute, Imperial College, London, UK

[2]National Heart and Lung Institute, Imperial College London, London, UK

[3]Royal Brompton and Harefield Hospitals, London, UK

[4]Radiology, Faculty of Medicine, Catholic University of the Sacred Heart, Roma, Italy

[5]Pharmacology, Faculty of Medicine, Catholic University of the Sacred Heart, Roma, Italy

[6]Depatment of Respiratory Sciences. University of Leicester, Leicester, United Kingdom

[7]Department of Pulmonology, Amsterdam UMC, University of Amsterdam, Amsterdam, The Netherlands

[8]Department of Medicine Huddinge, Karolinska Institutet, Stockholm, Sweden

[9]Department of Respiratory Medicine, Karolinska University Hospital, Stockholm, Sweden

[10]European Institute for Systems Biology and Medicine, CNRS-ENS-UCBL-INSERM, Université de Lyon, France

[11]Department of Clinical Science, University of Bergen, Bergen, Norway

[12]Institute for Lung Health, NIHR Leicester Biomedical Research Centre, Department of Respiratory Sciences, University of Leicester, Leicester, UK

[13]Department of Biomedical and Biotechnological Sciences, University of Catania, Catania, Italy

[14]Assistance publique des Hôpitaux de Marseille - Clinique des bronches, allergies et sommeil, Aix Marseille Université, Marseille, France

[15]NIHR Southampton Respiratory Biomedical Research Unit and Clinical and Experimental Sciences, Southampton, UK
[16]Respiratory Medicine, Department of Translational Medicine, University of Ferrara, Ferrara, Italy
[17]Division of infection, immunity and respiratory medicine, School of biological sciences, University of Manchester, Manchester University NHS Foundation Trust, Manchester Academic Health Science Centre, Manchester, United Kingdom
[18]Semmelweis University, Budapest, Hungary
[19]Department of Women's and Children's Health, Karolinska Institutet, Stockholm, Sweden
[20]Institute of Environmental Medicine, Centre for Allergy Research, Karolinska Institutet, Stockholm, Sweden
[21]Samuel Bronfman Department of Medicine, Icahn School of Medicine at Mount Sinai Hospital, New York, NY, USA
[22]Department of Medicine Solna, Karolinska Institutet, Stockholm, Sweden
[23]Department of Translational Medicine, University of Piemonte Orientale, Novara, Italy
[24]LungenClinic Grosshansdorf and Department of Medicine, Christian Albrechts University, Member of the German Center for Lung Research (DZL), Kiel, Germany
[25]Division of Clinical Genetics and Molecular Biology, Department of Medicine, Jagiellonian University Medical College, Kraków, Poland
[26]Respiratory Research Unit, University of Nottingham, UK
[27]Centre for Respiratory Medicine and Allergy, Manchester University NHS Foundation Hospital Trust, University of Manchester, Manchester, UK
[28]Department of Pulmonology, University of Groningen, University Medical Center Groningen, Groningen, The Netherlands
[29]Biosci Consulting, Maasmechelen, Belgium

*Corresponding Authors:
Prof. Paolo Montuschi, M.D.
Visiting Professor
Imperial College London
National Heart and Lung Institute
Faculty of Medicine
Guy Scadding Building
RBH Campus
Dovehouse Street

London SW3 6LY
United Kingdom
e-mail: p.montuschi@imperial.ac.uk

Prof. Ian M Adcock
Imperial College London
National Heart and Lung Institute
Faculty of Medicine
Guy Scadding Building
Dovehouse Street
London SW3 6LY
United Kingdom
e-mail: ian.adcock@imperial.ac.uk

Introduction

High resolution computed tomography (HRCT) of the lungs provides a comprehensive evaluation of the tracheo-bronchial tree and lung parenchyma that has been applied to various lung diseases, which has in turn been linked to disease control and lung function measurements.

In asthma, quantitative high resolution computed tomography (HRCT) (qCT) has revealed that the extent of bronchial wall thickening is related to airflow obstruction, greater bronchodilator response [1,2], asthma control scores and severity [3-5]. In addition, the presence of air trapping measured by qCT has been associated with severe asthma, greater airflow limitation and neutrophilic inflammation [6]. qCT measurements of the lungs also were distinct according to clinical phenotypes of asthma derived from cluster analysis of clinical features and outcomes were also distinguished by distinct qCT measurements that reflect varying degrees of airway wall thickening and dilatation, and air trapping [2,7]. Furthermore, qCT phenotypes themselves are also characterized by varying degrees of luminal and airway wall measurements and of air trapping, with concomitant differences reflected in asthma severity and degrees of airflow obstruction [8-10]

In view of the established usefulness of qCT in defining clusters that have distinctive clinical features, we used qCT measurements obtained from HRCT scans of the U-BIOPRED asthma cohort [11]. In the current study, we linked these qCT clusters to molecular pathways derived from an analysis of transcriptomic and proteomic measurements obtained in these patients. Thus, we applied a *radio-multiomics* approach to linking molecular pathways to qCT-derived clusters. We hypothesized that the association of *-omics* data obtained from different airway compartments and blood would reveal potential pathways associated with individual phenotypes of severe asthma as identified by lung HRCT scans.

Methods

qCT clusters external replication

Using ATLANTIS, a multinational prospective asthma cohort including approximately 1000 individuals in 29 centres across 9 countries [12], our external replication plan involved these steps: (1) measure compatibility between U-BIOPRED and ATLANTIS physio-demographics and qCT, (2) match cohorts for asthma severity and demographic characteristics (3) use the same qCT measurements and machine learning workflow in both cohorts, and (4) examine whether qCT clusters identified in ATLANTIS were similar to U-BIOPRED qCT clusters.

Due to between cohort differences, we added another computational step prior to clustering CT measurements in ATLANTIS to build a cohort comparable to that of U-BIOPRED cohort. We performed semi-parametric and non-parametric matching methods using the MatchIt R package [13] between U-BIOPRED and the ATLANTIS cohorts. The MatchIt R function was developed to produce more robust inferences and less sensitivity to modelling assumptions. It enabled us to identify a sub-population of the ATLANTIS cohort that matches U-BIOPRED based on age, sex, GINA classification of asthma severity, history of asthma exacerbations, lung function testing and oral corticosteroid therapy. To be agnostic to the various matching methods, we applied the Matchit function for 500 different random seeds for the following methods: nearest neighbour, 'optimal matching, full matching, genetic matching and coarsened exact matching and selected ATLANTIS subjects who were matched to U-BIOPRED subjects at least 1000 times. No CT measurements were included in a matching step, ensuring that the subjects' qCT features were not included in the cohort building; therefore, ATLANTIS CT clusters can be used to replicate the U-BIOPRED qCT clusters.

Integration of CT data and clustering

Missing values were imputed using random forest (R package missForest, function missForest). Before clustering, we applied standard scaling on the CT measurements (mean=0, variance=1).

All analyses were performed in R version 4.1.2 (www.r-project.org) and Cytoscape 3.9.1 [14]. Fifty qCT parameters were analysed (**Supplementary Table S2)**. When two or more parameters were highly correlated (absolute Spearman rho ≥0.85), only one of them was selected, giving 12 inspiratory parameters and 10 expiratory parameters for analysis (**Table 2**).

Consensus clustering (R package ConsensusClusterPlus), a resampling method that considers the cluster consensus across multiple runs of a clustering algorithm, was used to find the optimal cluster number by locating a cluster number k where the consensus matrix histogram approximates a bimodal distribution at the k cluster and a relatively small increase of the area under curve of the cumulative distribution function at the k+1 cluster yielded [15].

Machine learning and classification methods:

To gauge the efficacy of CT measurements data for predicting RACs and confirm the reproducibility across cohorts, we conducted five experiments. Two intra-cohort experiments explored various aspects within the same cohort while two inter-cohort experiments explored findings across different cohorts. Finally, combined analysis shows the added power of collating two cohorts.

In intra-cohort and combined experiments, we applied a five-fold cross-validation method to assess the performance of a partial least squares-linear discriminant analysis (PLS-LDA) model. The model underwent nested three-fold cross-validation to optimize the number of components required by the partial least square linear discriminant classifier (PLS-LDA), ensuring accurate evaluation of its predictive abilities using both CT within in each dataset and the combined dataset.

For our inter-cohort experiments, we conducted two distinct analyses. In the first, we trained our classifier on the U-BIOPRED cohort and tested its performance on the matched ATLANTIS dataset. In addition, in the second analysis, we also swapped the training and testing datasets, utilizing matched ATLANTIS as the training set and U-BIOPRED as the testing set. This methodology allowed us to assess the extent to which our findings generalize across matched cohorts. In each

experiment, we assessed model performance using standard metrics: sensitivity, specificity, accuracy, and F1 score.

Integrated functional analysis

Batch/technical effects, age, sex, BMI, and administration of oral corticosteroids were considered as covariates in the linear regression model when analysing for differentially expressed genes/proteins regarding the groups of interest (R package limma).

As the first step, we performed differential gene and protein expression analysis across and between the RACs. This produced a numerical matrix of P values, where each row corresponds to a gene and each column to an omics dataset (evidence). When a gene or protein was missing in an omics dataset, we converted the score for this gene across the missing omics to one (**Supplementary Table S3-S5**).

P values were used to prioritize genes/proteins by ActivePathways, a framework utilizing P-value merging to combine signals at the gene or protein level (evidence), followed by ranked hypergeometric tests to identify enriched pathways and processes [16]. This approach enables interpretation of a series of omics datasets within the context of established biological knowledge and gene function and discovery of associations that may only become evident when multiple datasets are integrated [16].

To obtain a single P-value for each gene across the multiple omics datasets, these P-values were merged row-wise using a data fusion approach of P-value merging, i.e, Fisher or Brown method [17]. A Fisher's technique adds log P values (sum(-2log(p)) for each omics dataset and compares them to a chi-squared distribution to combine the P-values. Brown's approach extends Fisher's method by accounting for covariance in P values and when the tests of significance used to generate P values are not always independent, it is more appropriate [17]. As a result, the Brown's method is suggested for several omics integration approaches.

The output of the ActivePathways for each over-represented pathway was the integrated gene list, associated genes with significant Brown P-values, and evidence annotations for each pathway [16].

For each signature, we calculated the enrichment scores of the signature in the relevant omics datasets using a gene set variation analysis (GSVA) (R package, GSVA, function gsva, arguments abs.ranking=FALSE, mx.diff=TRUE) [18]. GSVA calculates sample wise enrichment scores directly from expression values [18].

Results

Participant characteristics

Clusters were clearly separated, with a slight overlap between RAC2 and RAC3 (**Figure 1E**), consistent with a mixed chronic bronchiolitis/gas-trapping/emphysematous-like pattern [19].

Quantitative HRCT

Interestingly, RAC2 (predominant airway remodeling) showed lower values of inspiratory percentile 15 (P<0.0001) and MLD SD (P<0.001) and higher values of CT lung volume adjusted for the phantom density rods (P<0.01) compared with RAC1 (predominant obesity), whereas inspiratory, but not expiratory, LA mean BSA (P<0.01) and inspiratory WA BSA (P<0.05) were significantly different in RAC3 (predominant emphysema) compared with RAC1 (**Table 2**).

RAC2 showed higher values of inspiratory WA mean (P=0.0312), inspiratory WA mean BSA (P=0.0181) and expiratory WA mean BSA (P=0.0032), reflecting airway wall thickness, than healthy control subjects, whereas were inspiratory LAA -950 HU (emphysema) (P=0.67) and expiratory LAA -856 HU (air trapping) (P=0.12) were similar. (**Supplementary Table S7**). Compared with healthy control subjects, inspiratory LAA -950 HU (P=0.0011) and expiratory LAA -856 HU (P<0.0001) were elevated in RAC3, whereas inspiratory WA mean (P=0.47), inspiratory WA mean BSA (P=0.30), expiratory WA mean BSA (P=0.19) and wall area as a percent of total area were similar (**Supplementary Table S8**).

These data support the commonly observed co-existence of peripheral airway pathological changes and emphysema, which is also shown by the PCA partial overlap of RAC2 and RAC3.

qCT external replication

In terms of clinical characteristics, U-BIOPRED and ATLANTIS cohorts were similar. ATLANTIS qCT cluster 1 subjects were obese (**Supplementary Table S12**); qCT cluster 2 showed higher pre-bronchodilator FEV_1, higher pre-bronchodilator FVC absolute values, higher pre-

bronchodilator FVC % predicted value compared to other qCT clusters (**Supplementary Table S12**). Compared to other ATLANTIS qCT clusters, ATLANTIS qCT cluster 3 subjects showed a higher RV, lower pre-bronchodilator FEV_1/FVC%, and lower pre-bronchodilator $FEF_{25\%-75\%}$, percentage of predicted value (**Supplementary Table S12**).

Analysis of CT cluster validation using PLS-LDA:

In the intra-cohort analysis, our PLS-LDA classifier exhibited strong performance within both the ATLANTIS and U-BIOPRED cohorts (**Supplementary Table S12**). In ATLANTIS, the classifier demonstrated a sensitivity of 0.88 (IQR 0.04), specificity of 0.94 (IQR 0.02), accuracy of 0.88 (IQR 0.05), and F1 score of 0.88 (IQR 0.04), while in UBIOPRED, it achieved a sensitivity of 0.91 (IQR 0.08), specificity of 0.95 (IQR 0.03), accuracy of 0.91 (IQR 0.04), and F1 score of 0.9 (IQR 0.05). Moving to the inter-cohort analysis, where the classifier was tested across ATLANTIS-U-BIOPRED and U-BIOPRED-ATLANTIS cohorts, sensitivities ranged from 0.73 to 0.8, specificities from 0.85 to 0.89, accuracies from 0.72 to 0.79, and F1 scores from 0.73 to 0.78. In the combined analysis across both cohorts, the classifier achieved consistent performance with a sensitivity of 0.87 (IQR 0.06), specificity of 0.93 (IQR 0.02), accuracy of 0.88 (IQR 0.05), and F1 score of 0.87 (IQR 0.06). Through this systematic analysis, we gained insights into the power of CT measurements for RACs prediction and ensured robustness and reproducibility across diverse matched cohorts.

Molecular Pathways of RACs

Serum and sputum proteomics were sufficient to detect all differences in pathway modulation between RAC1 and RAC3, whereas identification of molecular phenotype differences between RAC2 and RAC3 required a 9 omic-based platform and data integration (**Table 3, Figure 3**). Comparisons between RAC1 versus RAC3 and RAC2 versus RAC3 showed several significant pathways being activated or repressed (**Table 3, Figure 3**). In contrast, there were fewer molecular pathway differences between RAC1 and RAC2 (**Figure 3**).

Discussion

We propose a new unique radiomultiomics approach, which has the potential to improve personalized medicine in severe asthma by providing insights into disease mechanisms.

The greater increase in post-salbutamol FEV_1 percent, but not absolute values, over baseline observed in RAC3 compared with RAC2 might reflect either more room for improvement due to lower pre-bronchodilator FEV_1 values in RAC3 or a higher degree of airway hyperresponsiveness in this cluster or both.

In contrast, in RAC2, we observed down-regulation of an ECM organization pathway, as reflected by reduced MMP2 and MMP9 protein expression, which was recently postulated as a potential mechanism of asthma airway remodelling in primary human airway epithelial cells stimulated with chymase to mimic mast cell activation [20]. A role for NOS2 in mediating emphysematous remodeling in mice in vivo has been reported [21].

Within group comparisons of qCT biomarkers across qCT clusters in either U-BIOPRED or ATLANTIS group show a similar trend. However, values of qCT biomarkers were generally lower in the ATLANTIS group than in the U-BIOPRED group. These might reflect inter-site differences, including data acquisition and/or data analysis.

As the replication cohort was selected to match the demographic characteristics, asthma severity, and lung function of the U-BIOPRED cohort, the generalizability of the RACs in younger asthma individuals and/or with less severe asthma cannot be established.

References

1. Aysola RS, Hoffman EA, Gierada D, et al. Airway remodeling measured by multidetector CT is increased in severe asthma and correlates with pathology. *Chest* 2008; 134: 1183-1191.
2. Gupta S, Siddiqui S, Haldar P, et al. Quantitative analysis of high-resolution computed tomography scans in severe asthma subphenotypes. *Thorax* 2010; 65: 775-781.

3. Brillet PY, Grenier PA, Fetita CI, et al. Relationship between the airway wall area and asthma control score in moderate persistent asthma. *Eur Radiol* 2013; 23: 1594-1602.
4. Niimi A, Matsumoto H, Amitani R, et al. Airway wall thickness in asthma assessed by computed tomography. Relation to clinical indices. *Am J Respir Crit Care Med* 2000; 162: 1518-1523.
5. Chetta A, Foresi A, Del Donno M, et al. Airways remodeling is a distinctive feature of asthma and is related to severity of disease. *Chest* 1997; 111: 852-857.
6. Busacker A, Newell JD, Jr., Keefe T, et al. A multivariate analysis of risk factors for the air-trapping asthmatic phenotype as measured by quantitative CT analysis. *Chest* 2009; 135: 48-56.
7. Trivedi AP, Hall C, Goss CW, et al; NHLBI Severe Asthma Research Program (SARP). Quantitative CT characteristics of cluster phenotypes in the Severe Asthma Research Program cohorts. *Radiology* 2022: 210363.
8. Gupta S, Hartley R, Khan UT, et al. Quantitative computed tomography-derived clusters: redefining airway remodeling in asthmatic patients. *J Allergy Clin Immunol* 2014; 133: 729-738.e18.
9. Zhang X, Xia T, Lai Z, et al. Uncontrolled asthma phenotypes defined from parameters using quantitative CT analysis. *Eur Radiol* 2019; 29: 2848-2858.
10. Choi S, Hoffman EA, Wenzel SE, et al; National Heart, Lung and Blood Institute's Severe Asthma Research Program. Quantitative computed tomographic imaging-based clustering differentiates asthmatic subgroups with distinctive clinical phenotypes. *J Allergy Clin Immunol* 2017; 140: 690-700 e8.
11. Shaw DE, Sousa AR, Fowler SJ, et al.; U-BIOPRED Study Group. Clinical and inflammatory characteristics of the European U-BIOPRED adult severe asthma cohort. *Eur Respir J* 2015; 46:1308- 1321. Erratum in: *Eur Respir J* 2017; 49 (6): 1550779.
12. Kraft M, Richardson M, Hallmark B, et al.; ATLANTIS study group. The role of small airway dysfunction in asthma control and exacerbations: a longitudinal, observational analysis using data from the ATLANTIS study. *Lancet Respir Med* 2022; 10: 661-668.

13. Ho DE, Kosuke I, King G, et al. Matching as nonparametric preprocessing for reducing model dependence in parametric causal inference. *Political analysis* 2007; 15: 199-236.
14. Shannon P, Markiel A, Ozier O, et al. Cytoscape: a software environment for integrated models of biomolecular interaction networks. *Genome Res* 2003; 13: 2498-2504.
15. Wilkerson MD, Hayes DN. ConsensusClusterPlus: a class discovery tool with confidence assessments and item tracking. *Bioinformatics* 2010; 26: 1572-1573.
16. Paczkowska M, Barenboim J, Sintupisut N, et al.; PCAWG Drivers and Functional Interpretation Working Group; Reimand J; PCAWG Consortium. Integrative pathway enrichment analysis of multivariate omics data. *Nat Commun* 2020; 11 (1): 735.
17. Brown MB. 400: A method for combining non-independent, one-sided tests of significance. *Biometrics* 1975; 31: 987-992.
18. Hänzelmann S, Castelo R, Guinney J. GSVA: gene set variation analysis for microarray and RNA-seq data. *BMC Bioinformatics* 2013; 14: 7.
19. Hartley RA, Barker BL, Newby C, et al. Relationship between lung function and quantitative computed tomographic parameters of airway remodeling, air trapping, and emphysema in patients with asthma and chronic obstructive pulmonary disease: A single-center study. *J Allergy Clin Immunol* 2016; 137: 1413-1422.e12.
20. Zhao XO, Sommerhoff CP, Paivandy A, et al. Mast cell chymase regulates extracellular matrix remodeling-related events in primary human small airway epithelial cells. *J Allergy Clin Immunol* 2022: S0091-6749 (22) 00887-9.
21. Knudsen L, Atochina-Vasserman EN, Massa CB, et al.. The role of inducible nitric oxide synthase for interstitial remodeling of alveolar septa in surfactant protein D-deficient mice. *Am J Physiol Lung Cell Mol Physiol* 2015; 309: L959-969.

Abbreviation list (Figure 4)

Abbreviations: COL9A2, collagen type IX alpha 2 chain; COL23A1, collagen type XXIII alpha 1 chain; ECM, extracellular matrix; EGFR, epidermal growth factor receptor; ERBB2, Erb-B2 receptor tyrosine kinase 2; ERBB4, Erb-B4 receptor tyrosine kinase 4; IGF, insulin-like growth factor; IGFBP6, insulin like growth factor binding protein 6; LTBP2, latent transforming growth factor beta binding protein 2; MMP1, matrix metallopeptidase 1; MMP9, matrix metallopeptidase 9; NOS2, nitric oxide synthase 2; NTRK, neurotrophic receptor tyrosine kinase; PAK, p21-activated kinase; PECAM, platelet endothelial adhesion molecule; PIK3CA, phosphatidylinositol-4,5-biphosphate 3-kinase catalytic subunit alpha; PTPN11, protein tyrosine phosphatase non-receptor type 11; SCF, Stem cell factor; SDC1, syndecan 1; SEMA6A, semaphorin 6A; SHC1, SHC adaptor protein 1; TGFB1, transforming growth factor beta 1; TNC, tenascin C; TRKB, tyrosine kinase receptor B.

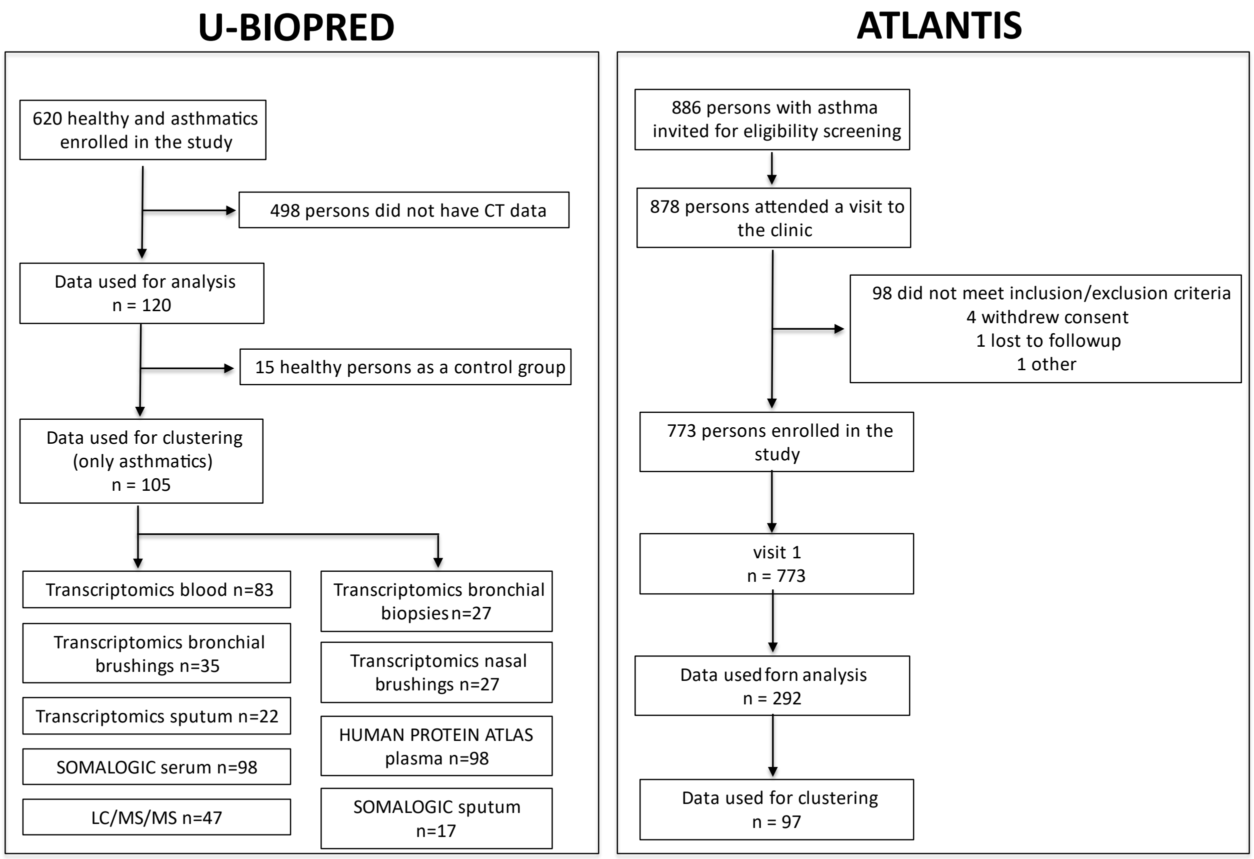

Supplementary Figure S1. Flowchart showing the study population selection process in the U-BIOPRED (discovery) and ATLANTIS (replication cohort). Selection process was driven by the availability of lung CT scan and multiomic data. The ATLANTIS replication cohort was matched to the U-BIOPRED cohort based on age, sex, GINA classification of asthma severity, history of asthma exacerbations, lung function testing and oral corticosteroid therapy. No CT measurements were included in the matching process.

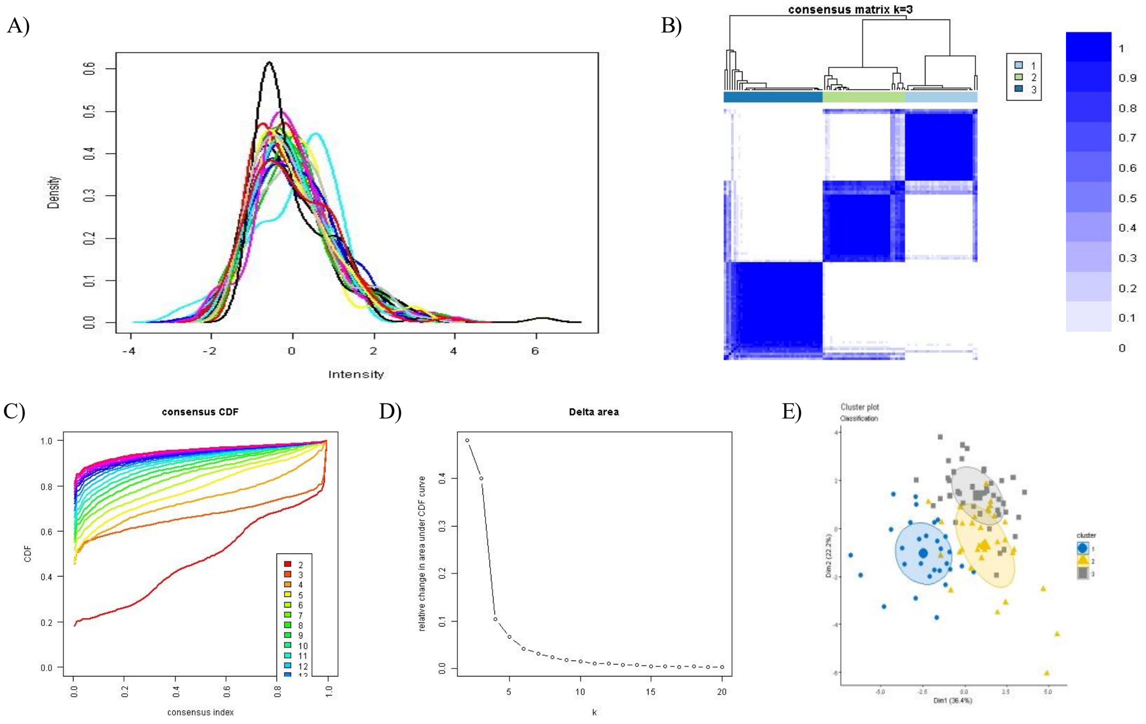

Supplementary Figure S2. Identification of asthma clusters based on quantitative CT. A) Density of individual qCT measurements, which are distinguished by various colours, after standard normalization. The optimal number of clusters was determined by finding a cluster number k where the consensus matrix histogram approximates a bimodal distribution at the k cluster and a relatively small increase in the area under the curve (AUC) of the cumulative distribution function (CDF) at the k+1 cluster yielded. B) Consensus matrix at k=3. C) CDF curves for cluster numbers k=2-9; at k=3 CDF curve approximates a bimodal distribution. D) AUC of the CDF k=2-9, where at the k=4 cluster yielded small increase. K=3 is the optimal number for clusters. E) Principal component analysis (PCA) showing that qCT radiomultiomic-associated clusters (RACs) are separated.

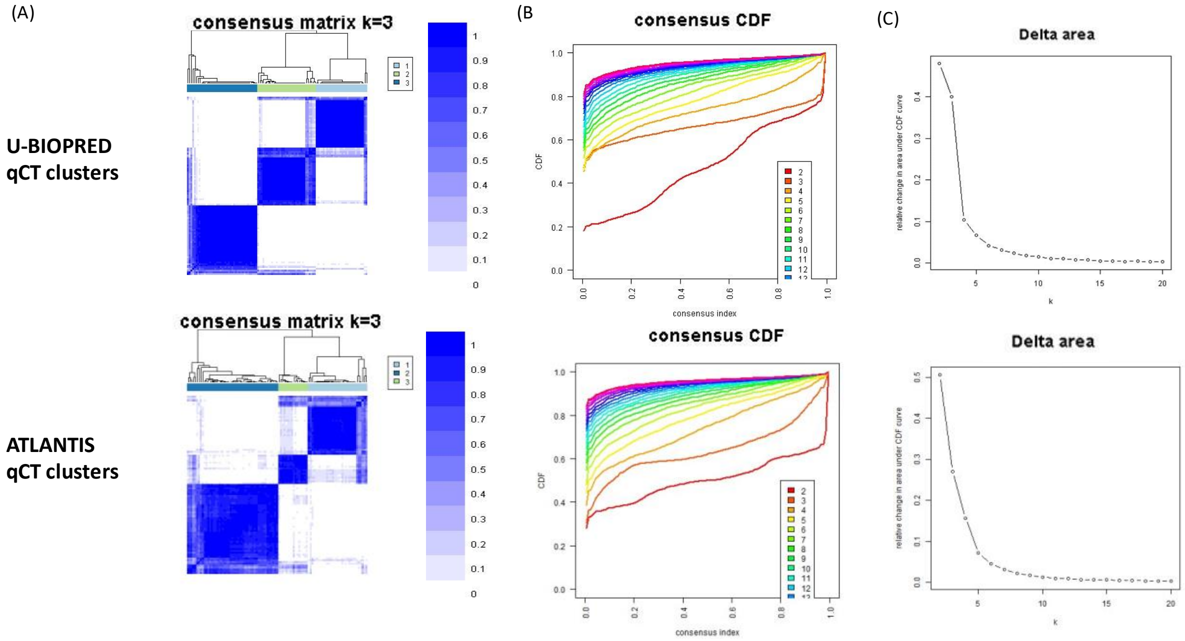

Supplementary Figure S3. Identification of severe asthma clusters in the U-BIOPRED discovery and ATLANTIS replication cohorts based on quantitative CT. A) Density of individual qCT measurements, which are distinguished by various colors, after standard normalization. The optimal number of clusters was determined by finding a cluster number k where the consensus matrix histogram approximates a bimodal distribution at the k cluster and a relatively small increase in the area under the curve (AUC) of the cumulative distribution function (CDF) at the k+1 cluster yielded. B) Consensus matrix at k=3. C) CDF curves for cluster numbers k=2-9; at k=3 CDF curve approximates a bimodal distribution. D) Principal component analysis (PCA) in ATLANTIS dataset showing that qCT clusters are separated.

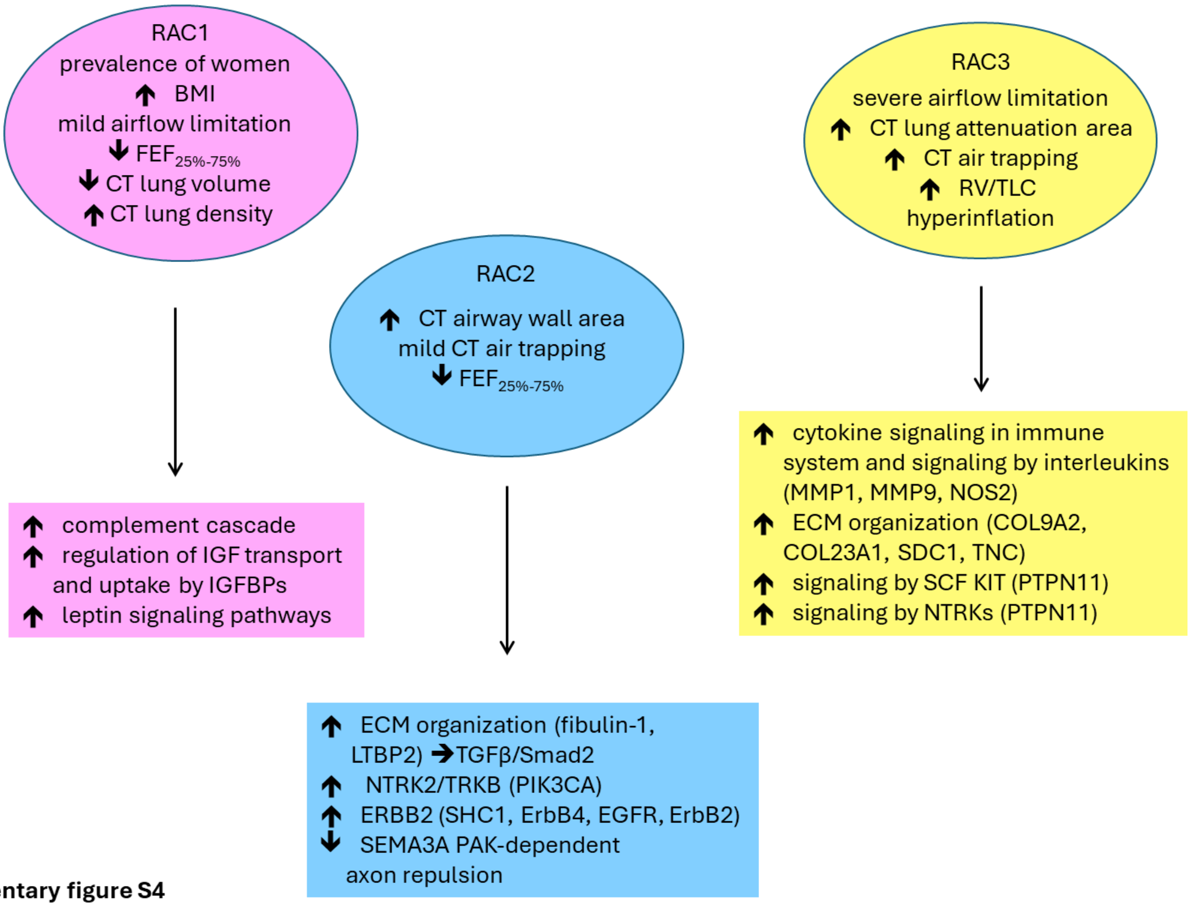

Supplementary figure S4

Supplementary Figure S4. Radiomultiomic-associated clusters (RACs): main characteristics and associations with modulation of selected endotypes. Gene expression and proteins having a role in pathways are shown in brackets.

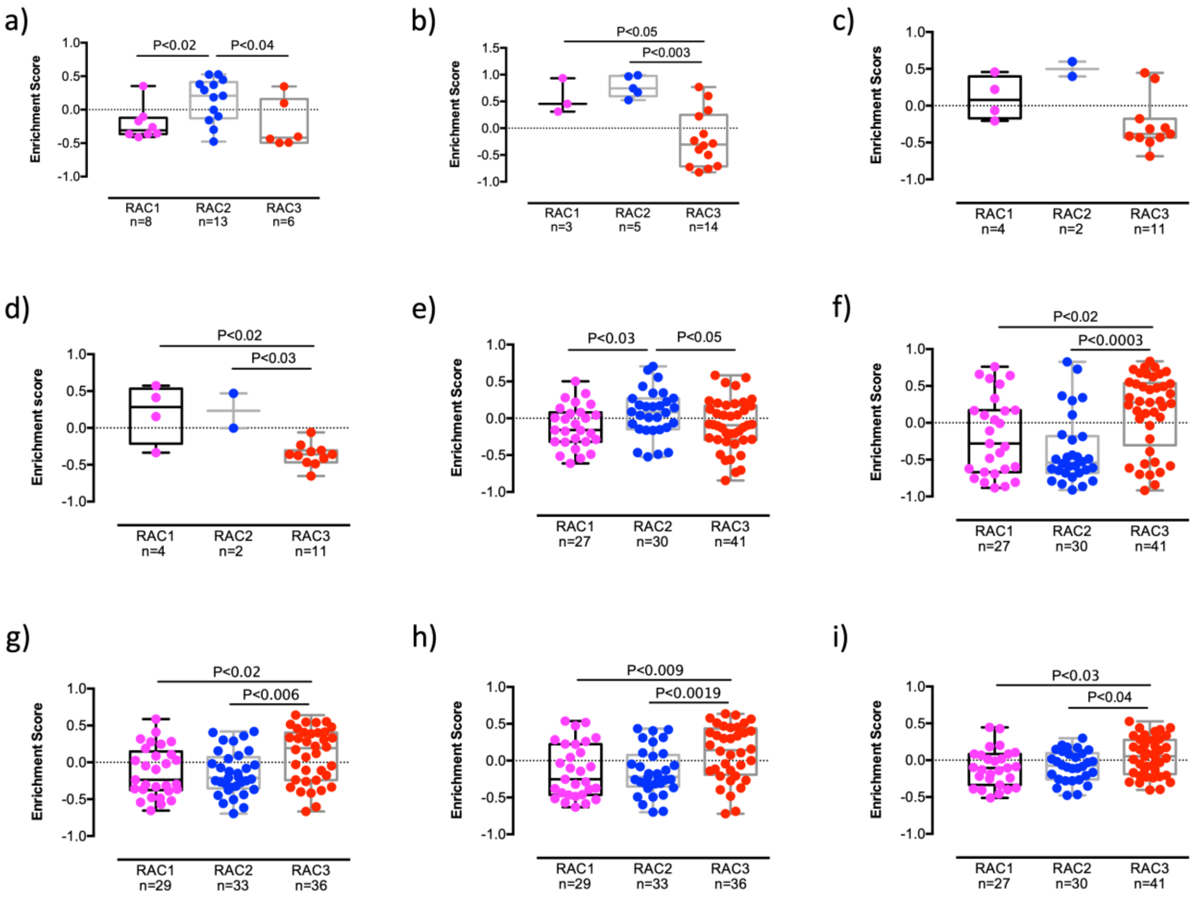

Supplementary Figure S5. Box and whiskers plots showing all points from minimum to maximum values of gene set variation analysis (GSVA) of pathways differentially expressed across radiomic-associated clusters (RACs). A) Bronchial biopsy transcriptomics, extracellular matrix organization; B) sputum transcriptomics, signaling by neurotrophic tyrosine kinase 2/tyrosine kinase receptor B (NTRK2/TRKB); C) sputum proteomics, extracellular matrix organization; D) sputum proteomics, signaling by interleukins; E) serum proteomics, signaling by Erb-B2 receptor tyrosine kinase 2 (ERBB2) in cancer; F) serum proteomics, semaphorin A4 p21-activated kinase (PAK)-dependent axon repulsion; G) plasma proteomics, cytokine signaling in immune system; H) plasma proteomics, signaling by interleukins; I) serum proteomics, extracellular matrix organization. Median values of enrichment scores with interquartile range are shown. Normality of variables was tested using

D’Agostino and Pearson omnibus normality test. Between-group comparisons were performed using Kruskal-Wallis test with Dunn’s multiple comparison test.

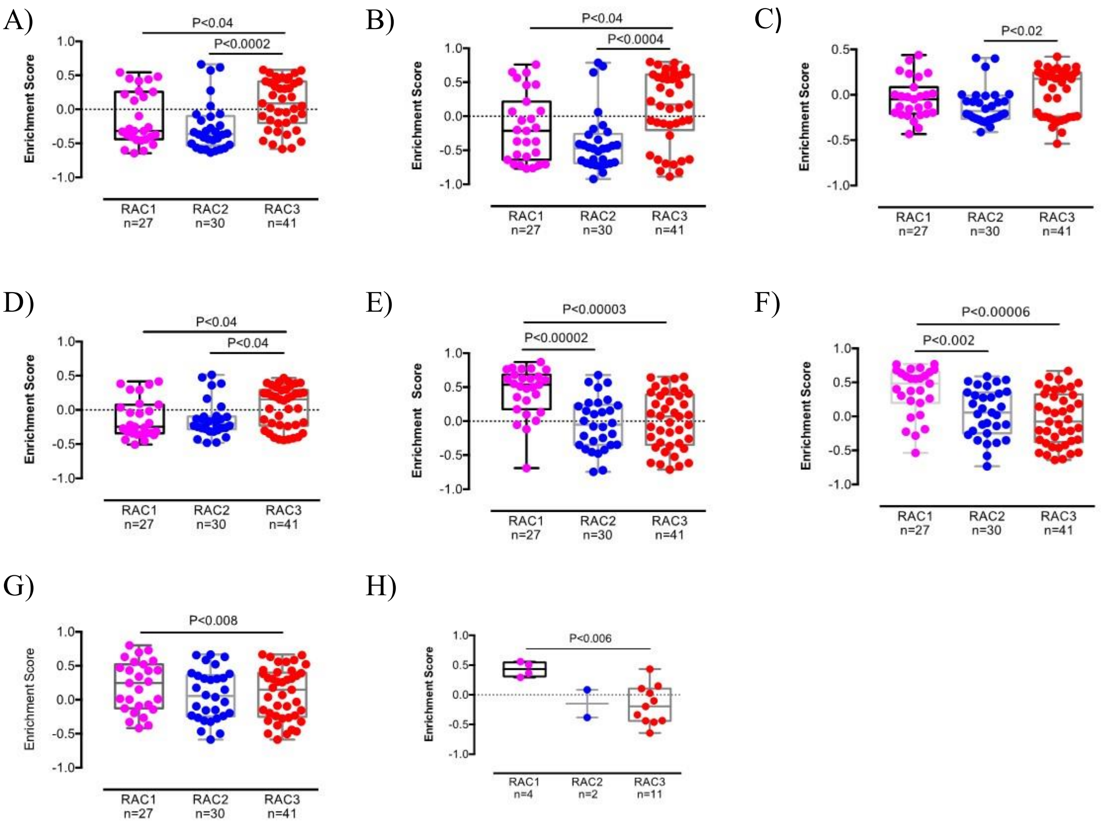

Supplementary Figure S6. Box and whiskers plots showing all points from minimum to maximum values of gene set variation analysis (GSVA) of pathways differentially expressed across radiomic-associated clusters (RACs). A) Serum proteomics, signaling by neurotrophic receptor tyrosine kinases (NTRKs); B) serum proteomics, signaling by neurotrophic receptor tyrosine kinase 2/tyrosine kinase receptor B (NTRK2/TRKB); C) serum proteomics, signaling by interleukins; D) serum proteomics, intracellular signaling by second messengers; E) serum proteomics, complement cascade; F) serum proteomics, complement cascade; G) serum proteomics, regulation of insulin-like growth factor (IGF) transport and uptake by IGF binding proteins (BPs); H) sputum proteomics, regulation of insulin-like growth factor (IGF) transport and uptake by IGF binding proteins (BPs). Median values of enrichment scores with interquartile range are shown. Normality of variables was

tested using D'Agostino and Pearson omnibus normality test. Between-group comparisons were performed using Kruskal-Wallis test with Dunn's multiple comparison test.

Definition of abbreviations of genes, proteins and pathways

A2M, Alpha-2 macroglobulin

ADAMTS5, ADAM metallopeptidase with thrombospondin type 1 motif 5

ADCYAP1, Adenylate cyclase activating polypeptide 1

ADCY5, Adenylate cyclase 5

AKT, AKT8 virus oncogene cellular homolog

ALB, Albumin

ANXA2, Annexin A2

AREG, Amphiregulin

ARHGEF11, Rho guanine nucleotide exchange factor 11

ARHGEF12, Rho guanine nucleotide exchange factor 12

BGN, Biglycan

BSG, Basigin, extracellular matrix metalloprotease inducer

BTK, Bruton tyrosine kinase

C1s, Complement C1s

C3, Complement factor 3

C5, Complement factor 5

C9, Complement factor 9

CAMK2, Calcium/calmodulin dependent protein kinase II

CAMK2B, Calcium/calmodulin dependent protein kinase II beta

CAPN12, Calpain 12

CAS, p130CAS, Crk-associated substrate

CASP3, Caspase 3

CFD, Complement factor D

CFH, Complement factor H

CFI, Complement factor I

CCL2, C-C motif chemokine ligand 2

CCL11, C-C motif chemokine ligand 11

CLEC1B, C-type lectin domain family 1 member B

COL6A6, Collagen type VI alpha 6 chain

COL9A2, Collagen type IX alpha 2 chain

COL23A1, Collagen type XXIII alpha 1 chain

CRK, CRK proto-oncogene adaptor protein

CRP, C reactive protein

CRTAP, Cartilage associated protein

CSF2, Colony stimulating factor 2

CSF3R, Colony stimulating factor 3 receptor

CSK, C-terminal Src kinase

CST3, Cystatin C

CTSB, Cathepsin B

CTSG, Cathepsin G

CXCL2, C-X-C motif chemokine ligand 2

DAP12, Death associated protein 12

DCN, Decorin

DDR2, Discoidin domain receptor tyrosine kinase 2

DMP1, Dentin matrix acidic phosphoprotein 1

DST, Dystonin

DUSP3, Dual specificity phosphatase 3

EGFR, Epidermal growth factor receptor

EIF4G2, Eukaryotic translation initiation factor 4 gamma 2

ERBB2, Erb-B2 receptor tyrosine kinase 2

ERBB4, Erb-B4 receptor tyrosine kinase 4

F2, Coagulation factor II, thrombin

F3, Coagulation factor III, tissue factor

F5, Coagulation factor V

F12, Coagulation factor XII

FBLN1, Fibulin 1

FCGR1A, Fc gamma receptor 1a

FCN1, Ficolin 1

FER, FER tyrosine kinase

FGA, Fibrinogen alpha chain

FGG, Fibrinogen gamma chain

FN1, Fibronectin 1

FSTL3, Follistatin like 3

FYN, FYN proto-oncogene, Src family tyrosine kinase

GP6, Glycoprotein VI platelet

GP9, Glycoprotein IX platelet

GPC3, Glypican 3

HAPLN1, Hyaluronan and proteoglycan link protein 1

HER2, Human epidermal growth factor receptor 2

HER4, Human epidermal growth factor receptor 4

HSP90, Heat shock protein 90

HSP90B1, Heat shock protein 90 beta family member 1

ICAM1, Intercellular adhesion molecule 1

IFI35, Interferon induced protein 35

IFNG, Interferon gamma

IGF, Insulin-like growth factor

IGFBP6, Insulin like growth factor binding protein 6

IL5, Interleukin 5

IL6, Interleukin 6

IL16, Interleukin 16

IL1RL1, Interleukin 1 receptor like 1

IL3RA, Interleukin 3 receptor subunit alpha

IL15RA, Interleukin 15 receptor subunit alpha

INSR, Insulin receptor

ITGA6, Integrin subunit alpha 6

JAK2, Janus kinase 2

JAM2, Junctional adhesion molecule 2

JAM3, Junctional adhesion molecule 3

KLK7, Kallikrein related peptidase 7

LAMA2, Laminin subunit gamma 2

LAMA4, Laminin subunit alpha 4

LCK3, Proto-oncogene tyrosine-protein kinase LCK 3

LTBP2, Latent transforming growth factor beta binding protein 2

LTBP4, Latent transforming growth factor beta binding protein 4

LYN, LYN proto-oncogene, Src family tyrosine kinase

MAPK1, Mitogen-activated kinase 1

MEP, Methylerythritol 4-phosphate

MFGE8, Milk fat globule EGF and factor VA/III domain containing

MMP1, Matrix metallopeptidase 1

MMP2, Matrix metallopeptidase 2

MMP9, Matrix metallopeptidase 9

MMP12, Matrix metallopeptidase 12

MMP17, Matrix metallopeptidase 17

MYH14, Myosin heavy chain 14

MYL12B, Myosin light chain 12B

NFKB2, Nuclear factor kappa B subunit 2

NOS2, Nitric oxide synthase 2

NRG1, Neuregulin 1

NTRK, Neurotrophic tyrosine kinase

PAK, p21-activated kinase

PAPPA, Pappalysin 1

PDPK1, 3-Phosphoinositide dependent protein kinase 1

PECAM1, Platelet endothelial adhesion molecule 1

PF4, Platelet factor 4

PIK3CA, Phosphatidylinositol-4,5-biphosphate 3-kinase catalytic subunit alpha

PLCG1, Phospholipase C gamma 1

PLG, Plasminogen

PLXNA2, Plexin A2

PPIA, Peptidylprolyl isomerase A

PPIB, Peptidylprolyl isomerase B

PRCP, Polycarboxypeptidase

PRKACA, Protein kinase CAMP-activated catalytic subunit alpha

PRKCA, Protein kinase C alpha

PRKCB, Protein kinase C beta

PRKCD, Protein kinase C delta

PRKCQ, Protein kinase C theta

PSMD5, Proteasome 25S subunit, non-ATPase 5

PTPN6, Protein tyrosine phosphatase non-receptor type 6

PTPN11, Protein tyrosine phosphatase non-receptor type 11

R, The R Project for Statistical Computing

RAC1, Rac family small GTPase 1

RAE1, Ribonucleic acid export 1

RHOB, Ras homolog family member B

ROCK1, Rho associated coiled-coil containing protein kinase 1

ROCK2, Rho associated coiled-coil containing protein kinase 2

RPS6KA3, Ribosomal protein S6 kinase A3

SCF, Stem cell factor

SDC1, Syndecan 1

SEMA3A, Semaphorin 3A

SEMA6A, Semaphorin 6A

SERPINE1, Serpin family E member 1

SHC1, SHC adaptor protein

SHP2, SH2 domain-containing protein tyrosine phosphatase 21

SPHK1, Sphingosine kinase 1

SPARCL1, SPARC like 1

SRC, SRC proto-oncogene, Src family tyrosine kinase

TEC, Tec protein tyrosine kinase

TGFB1, Transforming growth factor beta 1

THBS1, Thrombospondin 1

TNC, Tenascin C

TNFSF9, TNF superfamily member 9

TNFRSF8, TNF receptor superfamily member 9

TRIM10, Tripartite motif containing 10

TRKB, Tyrosine kinase receptor B

TSLP, Thymic stromal lymphopoietin

UBE2N, Ubiquitin conjugating enzyme E2 N

YES1, YES, proto-oncogene 1, Src family tyrosine kinase

Supplementary Table S1. Characteristics of study group participants classified on the basis of the degree of asthma severity

	San		SAs/ex-		MMA		HS		Overall P value
	Mean ± SEM or median (IQR)	n	Mean ± SEM or median (IQR)	n	Mean ± SEM or median (IQR)	n	Mean ± SEM or median (IQR)	n	
Participants, n		73		20		12		15	
Gender, % females	62%	73	50%	20	33%	12	40%	15	Ns
Age, years	^{a}52.0 (44.0-60.0)	73	^{b,}53.5 (47.3-61.8)	20	35.5 (25.5-53.0)	12	^{a,,b}32.0 (24.0-48.0)	15	0.0004
BMI, kg/m^2	30.4 (25.2-36.2)	73	29.1 (25.2-35.0)	20	25.4 (23.2-29.3)	12	32.0 (24.0-48.0)	15	Ns
Asthma exacerbations in previous 12 months	^{c}2.0 (1.0-4.0)	73	^{a}2.0 (0.3-4.0)	20	^{a,c}0 (0-0)	12	not applicable		<0.0001
Pre-bronchodilator FEV_1, L	^{a,c}2.0 (1.3-2.6)	72	^{b,d}1.7 (1.2-3.4)	20	^{a,b}3.2 (2.3-4.0)	12	^{c,d}3.5 (2.8-4.1)	15	<0.0001
Pre-bronchodilator FEV_1, % predicted value	^{a,c}67.6±3.0	72	^{b,e}69.3±4.7	20	^{a,e}92.3±4.8	12	^{b,c}97.3±3.9	15	<0.0001
Pre-bronchodilator FVC, L	^{c,f}3.1 (2.4-3.7)	72	^{a,g}3.1 (2.6-3.7)	20	^{a,c}4.6 (4.3-5.2)	12	^{f,g}4.4 (3.6-5.3)	15	<0.0001
Pre-bronchodilator FVC, % predicted value	^{b,e}86.8±2.4	72	91.7±4.1	20	^{e}108.4±3.7	12	^{b}102.9±3.9	15	0.0004
Pre-bronchodilator FEV_1/FVC, %	^{f}65.5 (49.7-75.2)	72	^{h}58.6 (48.6-70.0)	20	70.2 (64.4-74.2)	12	^{f,h}79.2 (76.5-82.3)	15	0.0002
Pre-bronchodilator $FEF_{25\%-75\%}$, L/s	^{a,c}1.0 (0.5-1.9)	72	^{g,h}1.0 (0.7-1.5)	20	^{a,g}2.6 (1.6-3.5)	12	^{c,h}3.5 (2.5-4.0)	15	<0.0001
Pre-bronchodilator $FEF_{25\%-75\%}$, % predicted value	^{c}38.5±3.4	72	^{d}35.6±4.8	20	59.2±6.2	12	^{c,d}77.1±4.7	15	<0.0001
Pre-bronchodilator PEF, % predicted value	^{a,b}72.6 (50.9-90.6)	70	78.7 (54.9-92.1)	19	^{a}102.0 (82.8-127.9)	11	^{b}94.4 (85.5-111.1)	15	0.0005
RV, L	^{a}2.5 (2.0-3.1)	57	^{b}2.4 (2.0-3.2)	16	1.9 (1.1-2.5)	10	^{a,b}1.6 (1.3-1.7)	13	0.0007
TLC, L	6.1±0.2	57	6.5±0.4	16	6.8±0.3	10	6.3±0.4	13	Ns
RV/TLC, %	^{a,f}41.6±1.7	57	^{b,g}40.7± 2.4	16	^{g}27.8±3.5	10	^{b,f}25.4±1.8	13	<0.0001
sGaw, 1/kPa*s	^{a}0.7 (0.3-1.2)	21	0.9 (0.3-1.4)	23	1.2 (0.6-2.3)	34	^{a}1.8 (1.1-2.0)	11	0.0047
[1]OCS use, daily to monthly	60%	72	60%	20	not applicable	0	not applicable	0	Ns
ICS use, at least b.i.d.	^{a}61%	69	^{g}58%	19	^{a,g}100%	12	not applicable	0	0.0247
LABA use, at least b.i.d.	47%	70	29%	19	not applicable	0	not applicable	0	Ns
Omalizumab	16%	73	33%	20	not applicable	0	not applicable	0	Ns
ACQ.5, average score	^{a}2.2±0.1	69	^{g}2.4±0.3	19	^{a,g}0.9±0.3	8	not applicable	0	0.0090
AQLQ, average score	^{f,h}4.6 (3.8-5.4)	72	4.7 (3.9-5.2)	20	^{f,h}6.5 (5.7-6.8)	12	not applicable	0	0.0001
ESS, total score	6.0 (3.0-10.0)	66	^{g}8.0 (5.8-12.3)	18	^{g}3.0 (2.0-6.0)	11	not applicable	0	0.0287
HADS, total score	^{g}11.1±1.0	72	^{a}15.9±2.8	19	^{a,g}4.0±1.2	11	not applicable	0	0.0025
SNOT, total score	^{g}27.6±1.8	71	27.5±5.4	20	^{g}13.4±3.4	12	not applicable	0	0.0247
F_ENO, ppb	33.0 (20.0-60.5)	67	18.5 (11.5-51)	19	33.8 (19.6-59.3)	12	19.0 (12.0-29.0)	15	^{i}0.0137
[1]Serum total IgE, IU/ml	^{a}140.0 (44.0-379.0)	71	^{f}310.0 (75.2-65.0)	19	84.6 (24.9-192.5)	12	^{a,f}19.0 (4.8-55.2)	14	0.0003

Serum hsCRP, mg/L	[a]2.1 (1.1-4.7)	73	[b,f]3.4 (1.6-7.2)	20	[a,f]0.6 (0.1-1.1)	12	[b]1.0 (0.2.2.6)	15	<0.0001
Serum periostin, ng/ml	45.8 (40.6-55.6)	65	42.1 (34.8-53.0)	17	52.9 (39.0-58.6)	11	55.1 (41.5-60.2)	13	Ns
Plasma eotaxin, pg/ml	105.0 (75.6-130.5)	69	113.5 (69.2-158.5)	18	84.5 (63.1-96.0)	12	83.2 (66.0-130.0)	15	Ns
Serum IL-13, pg/ml	[e]0.7 (0.4-1.2)	65	0.5 (0.2-1.1)	17	0.7 (0.2-1.1)	11	[e]0.3 (0.2-0.6)	13	0.0488
Plasma interferon-γ, pg/ml	5.3 (3.7-8.0)	69	6.5 (5.4-13.6)	18	4.5 (3.0-8.0)	12	5.2 (5.4-7.4)	15	Ns
Plasma IL-6, pg/ml	[a,f]10.1 (5.6-15.7)	69	[b,j]11.3 (6.0-18.2)	18	[a,b]4.3 (1.6-5.5)	12	[f,j]3.4 (1.6-5.3)	15	<0.0001
Plasma IL-8, pg/ml	[e]31.8 (23-7-45.9)	69	30.9 (24.7-49.4)	18	25.1 (23.1-32.6)	12	[e]21.8 (17.8-31.7)	15	0.0013
Plasma TNF-α, pg/ml	1.8 (1.4-2.2)	69	[e]2.1 (1.8-2.2)	18	1.6 (1.3-2.1)	12	[e]1.5 (1.1-1.7)	15	0.0434
[2]Skin prick test negative, n	[a]7	53	4	15	1	9	[a,]6	11	0.0154
Blood eosinophils, cells/μl	225 (100-457.5)	72	[e]300 (200-540)	19	100 (100-475)	12	[e]100 (100-120)	15	0.0251
Blood neutrophils, cells/μl	[e,f]4850 (3525-7325)	72	[b,h]6190 (4280-7900)	19	[f,h]2850 (2377.5-3250)	12	[b,e]3000 (1900-5100)	15	<0.0001
Blood lymphocytes, cells/μl	1700 (1307.5-2300)	72	2100 (1660-2500)	19	1805 (1420-2100)	12	1520 (1400-2100)	15	Ns
Blood white cells cells/μl	[b,f]8000 (6325-10180)	72	[c,h]6900 (5600-9500)	19	[c,f]5250 (4522.5-6000)	12	[b,h]5330 (4650-7000)	15	<0.0001
Blood monocytes, cells/μl	[e]545 (400-700)	72	[a,b]600 (500-840)	19	[b]400 (342.5-475)	12	[a,e]310 (300-500)	15	0.0005
Blood basophils, cells/μl	40 (0-100)	72	20 (0-100)	19	20 (0-100)	12	0 (0-100)	15	Ns
[3]Sputum eosinophils counts, %	4.1 (0.6-15.8)	31	4.8 (0.4-2.1)	12	0.8 (0.7-6.4)	7	0.4 (0-1.0)	4	Ns
Sputum neutrophil counts, %	52.7 (37.4-84.9)	31	48.3.6 (29.4-61.3)	12	33.1 (18.9-63.3)	7	29.0 (5.1-60.2)	4	Ns
Sputum macrophage counts, %	25.8 (8.9-46.1)	31	32.3 (12.7-59.8)	12	38.6 (20.2-70.0)	7	66.0 (36.9-93.7)	4	Ns
Sputum lymphocyte counts, %	0.8 (0.4-1.7)	31	0.9 (0.4-1.5)	12	0.9 (0.6-3.7)	7	1.1 (0.5-6.1)	4	Ns
Sputum mast cell counts, %	0.0 (0.0-0.0)	31	0.0 (0.0-0.04)	12	0.0 (0.0-0.0)	7	0.0 (0.0-0.0)	4	Ns

[1]Urinary glucocorticoid metabolites were measured in 53 out of 55 severe asthma participants on OCS to confirm adherence with OCS therapy. This was confirmed in 27 out of 53 severe asthma participants. [1]Atopy was defined based on results of a serum IgE assay. [2]Skin prick tests to common aeroallergens were performed. [3]Sputum cell counts were expressed as a percent of nonsquamous cells.

Normality of variables was tested using D'Agostino and Pearson omnibus normality test. Between-group comparisons were performed using ANOVA with Tukey's multiple comparison test for normally distributed data or Kruskal-Wallis test with Dunn's multiple comparison test for nonparametric data. Categorical variables were expressed as numbers or percent values. A chi-square test was used for multiple comparisons of categorical variables. If the chi-square test was positive, a Fisher exact test was used for pairwise comparisons of categorical variables.

[a]P<0.01; [b]P<0.01; [c]P<0.0001; [d]P<0.0001; [e]P<.0.05; [f]P<0.001; [g]P<0.05; [h]P<0.001; [i]Dunn's multiple comparison test negative; [j]P<0.01

Definition of abbreviations: ACQ, Asthma Control Questionnaire mean of 1-5; AQLQ, Asthma Quality of Life Questionnaire total mean; b.i.d., twice daily; BMI, body mass index; ESS, Epworth sleepiness scale; $FEF_{25\%-75\%}$, forced expiratory flow at 25%-75% of forced vital capacity, F_ENO, fractional exhaled nitric oxide; HADS, hospital anxiety and depression scale; HS, healthy subjects; hsCRP, high-sensitivity C-reactive protein; ICS, inhaled corticosteroids; IL, interleukin; IQR, interquartile range; LABA, long-acting β_2-adrenergic receptor agonists; MMA, mild-to-moderate asthma; OCS, oral corticosteroids; ppb, parts per billion; PEF, peak expiratory flow; RV, residual volume; SAn, severe asthma non-smokers; SAs/ex-, severe current or ex-smokers with a smoking history ≥5 pack/years; asthma sGaw. specific airway conductance; SNOT 20, sino-nasal outcomes test; TLC, total lung capacity; TNF, tumor necrosis factor.

Supplementary Table S2. Inspiratory and expiratory quantitative CT parameters measured in the U-BIOPRED study

Body Surface Area (BSA) (Mostseller formula)
CT lung volume adjusted for the phantom density rods
CT lung volume adjusted expressed as percent of 90th percentile of total lung capacity (TLC) predicted value
CT lung volume adjusted expressed as percent of total lung capacity (TLC) predicted value
Percent of low attenuation area (LLA) with CT numbers lower than -856 Hounsfield Units (HU)
Percent of low attenuation area (LLA) with CT numbers lower than -950 Hounsfield Units (HU)
Lumen area (LA) standard deviation (SD) of mean values of right and left lung segmental bronchi
Lumen area (LA) average of mean values of right and left lung segmental bronchi
Lumen area (LA) average of mean values of right and left lung segmental bronchi standardized by body surface area
Lumen area (LA) average of median values of right and left lung segmental bronchi
Lumen area (LA) average of median values of right and left lung segmental bronchi standardized by body surface area
Mean lung density (MLD)
Mean lung density (MLD) standard deviation (SD) of CT number frequency distribution;
Mean lung density (MLD) adjusted for the phantom density rods
Wall area (WA) as a percent of total area (TA)
15th percentile of the cumulative frequency distribution of CT numbers
15th percentile of the cumulative frequency distribution of CT numbers adjusted for the phantom density rods
Total area (TA) standard deviation (SD) of mean values of right and left lung segmental bronchi
Total area (TA) average of mean values of right and left lung segmental bronchi
Total area (TA) average of mean values of right and left lung segmental bronchi standardized by body surface area
Total area (TA) average of median values of right and left lung segmental bronchi
Total area (TA) average of mean values of right and left lung segmental bronchi standardized by body surface area
Wall area (WA) standardized by body surface area
Wall area (WA) average of mean values of right and left lung segmental bronchi
Wall area (WA) average of median values of right and left lung segmental bronchi

Volumetric whole lung CT scans were obtained at total lung capacity (TLC) and residual volume (RV) following a standardized protocol for each model and scanner manufacturer.

Supplementary Table S6. Full clinical characteristics of Radiomultiomic-associated clusters (RAC)

	RAC1		RAC2		RAC3		ANOVA or Kruskal-Wallis or $\chi 2$ P value	RAC1 vs RAC2	RAC1 vs RAC3	RAC2 vs RAC3
	**Mean ± SEM ormedian (IQR)**	**n**	**Mean ± SEM or median (IQR)**	**n**	**Mean ± SEM or median (IQR)**	**N**				
Participants, n		30		34		41				
Gender, % females	77% (n=23)	30	56% (n=19)	34	41% (n=17)	41	**0.0128**	0.1145	**0.0039**	0.2508
Age, years	48.4±2.6	30	49.9±2.9	34	52.4±1.7	41	0.6033	0.6778	0.1870	0.3874
BMI, kg/m^2	36.8±1.4	30	27.9±0.8	34	27.8±0.8	41	**<0.0001**	**<0.0001**	**<0.0001**	0.9455
Mild-to-moderate asthma persons, n	1	30	7	34	4	41	0.0874	0.0575	0.3881	0.2092
Asthma exacerbations in previous 12 months	2.5 (1.0-4.0)	30	2.0 (0-4.0)	34	1.0 (0-3.0)	41	0.0851	0.1997	**0.0237**	0.9180
Pre-bronchodilator FEV_1, L	2.0 (1.5-2.5)	30	2.5 (2.0-3.3)	34	1.6 (1.1-2.4)	40	**0.0012**	**0.0213**	0.1879	**<0.001**
Pre-bronchodilator FEV_1, % predicted value	70.6 (59.6-90.1)	30	86.5 (71.9-98.1)	34	50.5 (38.5-78.2)	40	**<0.0001**	**0.0393**	**0.0106**	**<0.0001**
Pre-bronchodilator FVC, L	2.9±0.2	30	3.7±0.2	34	3.3±0.2	40	**0.0154**	**<0.05**	0.0872	0.2110
Pre-bronchodilator FVC, % predicted value	86.0±3.8	30	99.8±2.8	34	84.5±3.2	40	**0.0017**	**0.0042**	0.7561	**0.0006**
Pre-bronchodilator FEV_1/FVC, %	70.8±2.6	30	70.4±1.5	34	53.9±1.9	40	**<0.0001**	0.8769	**<0.0001**	**<0.0001**
Pre-bronchodilator $FEF_{25\%-75\%}$, L/s	1.3 (0.8-2.1)	30	1.7 (0.9-2.5)	34	0.7 (0.4-1.0)	40	0.0001	0.2705	**0.0093**	**<0.0001**
Pre-bronchodilator $FEF_{25\%-75\%}$, % predicted value	37.6 (22.7-57.9)	30	46.0 (27.5-65.5)	34	19.1 (13.1-37.8)	40	**<0.0001**	0.3366	**0.0069**	**<0.0001**
Pre-bronchodilator PEF, % predicted value	82.1 (64.4-92.2)	30	87.7 (74.3-103.3)	32	58.9 (42.0-80.1)	38	**<0.0001**	0.0974	**0.0087**	**<0.0001**
Change in FEV_1, post-salbutamol, %	12.6 (3.0-29.8)	30	10.3 (4.2-13.8)	34	19.1 (6.6-27.1)	40	**0.0181**	0.2356	0.3138	**0.0022**
RV, L	2.0 (1.4-2.6)	23	2.1 (1.5-2.5)	26	2.8 (2.5-3.5)	34	**<0.0001**	0.6230	**<0.001**	**<0.001**
TLC, L	5.0 (4.7-5.6)	23	6.0 (5.0-7.0)	26	7.3 (5.9-7.9)	34	**<0.0001**	**0.0019**	**<0.0001**	**0.0040**
RV/TLC	0.39±0.03	23	0.35±0.02	26	0.44±0.02	34	**0.0191**	0.2775	0.1412	**0.0035**
sGaw, 1/kPa*s	0.6 (0.2-1.8)	21	1.1 (0.7-1.8)	23	0.6 (0.3-0.8)	34	**0.0104**	0.1654	0.3284	**0.0012**
[1]OCS use, daily to monthly	66%	30	38%	34	55%	41	0.0711	**0.0273**	0.4600	0.1686

ICS use, at least b.i.d.	83%	30	59%	34	71%	41	0.1001	0.0538	0.2686	0.3341
LABA use, at least b.i.d.	47%	30	31%	32	33%	39	0.3900	0.2975	0.3229	1
Omalizumab	13%	30	21%	34	15%	41	0.6913	0.5201	1	0.5512
ACQ.5, average score	2.4±0.2	29	1.7±0.2	30	2.3±0.2	37	0.0604	**0.0195**	0.6292	0.0669
AQLQ, average score	4.5±0.2	29	5.0±0.2	34	4.7±0.2	41	0.2191	0.0553	0.5277	0.2628
ESS, total score	6.5 (2.8-9.3)	26	6.0 (3.0-10.5)	32	6.0 (3.0-9.0)	37	0.9675	>0.9999	0.8211	0.8405
HADS, total score	8.0 (6.5-18.5)	29	9.5 (4.3-19.3)	28	8.3 (5.0-18.0)	40	0.9378	0.9273	0.6698	0.9481
SNOT, total score	25.5 (17.3-34.0)	30	22.0 (10.3-32.0)	34	28.0 (13.0-40.3)	40	0.3102	0.4123	0.5811	0.1231
Current/ex-smokers/non-smokers, n	0/9/21	30	2/7/259	34	5/11/256	41	0.2913	0.3077	0.1396	0.4643
Pack-years	10.0 (1.7-15.3)	9	5.2 (4.023.0)	9	10.0 3.0-22.5)	15	0.7241	0.4885	0.5483	0.8264
F_ENO, ppb	26.0 (14.5-79.5)	25	32.3 (18.1-50.4)	34	30.0 (19.0-59.0)	39	0.9756	0.9787	0.8350	0.8755
[2]Serum total IgE, IU/ml	120.0 (21.3-417.0)	29	135.5 (51.4-558.5)	32	193.0 (72.8-364.0)	40	0.4831	0.3092	0.8416	0.2854
Serum hsCRP, mg/L	5.3 (1.6-7.9)	30	1.9 (0.9-3.9))	34	1.8 (0.9-3.2)	41	**0.0076**	**0.0170**	**0.0022**	0.6894
Serum periostin, ng/ml	44.3 (39.8-55.6)	29	48.8 (39.0-62.4)	31	46.1 (38.4-56.9)	33	0.5159	0.2615	0.4135	0.8173
Plasma eotaxin, pg/ml	104.5 (67.7-134.8)	30	95.4 (67.8-119.5)	33	106.5 (84.1-148.0)	36	0.5081	0.4294	0.8006	0.2594
Serum IL-13, pg/ml	0.5 (0.3-0.8)	29	0.7 (0.4-1.1)	31	0.9 (0.3-1.4)	33	0.1452	0.1391	0.0659	0.6380
Plasma interferon-γ, pg/ml	5.2 (3.6-8.7)	30	6.1 (3.7-9.7)	33	5.1 (3.8-7.5)	36	0.6007	0.6249	0.7368	0.2908
Plasma IL-6, pg/ml	12.1 (8.0-19.5)	30	6.6 (4.0-13.4)	33	6.6 (3.7-13.1)	36	**0.0030**	**0.0028**	**0.0022**	0.9881
Plasma IL-8, pg/ml	30.7 (22.3-38.8)	30	30.1 (23.7-38.7)	33	33.5 (24.7-50.0)	36	0.5191	0.62199	0.2550	0.5326
Plasma TNF-α, pg/ml	1.8 (1.4-2.2)	30	1.8 (1.5-2.2)	33	1.7 (1.4-2.2)	36	0.9049	0.9265	0.7564	0.6738
[3]Skin prick test positive/negative, n	18/4	22	21/6	27	26/2	28	0.2817	1	0.3849	0.1430
Blood eosinophils, cells/μl	180 (100-300)	29	200 (100-500)	33	300 (180-500)	41	0.0781	0.1308	**0.0313**	0.3738
Blood neutrophils, cells/μl	4700 (3600-7050)	29	4200 (3050-6445)	33	4690 (3295-7600)	41	0.5306	0.2419	0.5396	0.6323
Blood lymphocytes, cells/μl	1700 (1135-2100)	29	2100 (1600-2270)	33	1700 (1395-2400)	41	0.2850	0.0708	0.4711	0.5606
Blood white cells cells/μl	8700±530	29	7742±397	33	8369±469	41	0.3745	0.1468	0.6441	0.3249
Blood monocytes, cells/μl	500 (400-780)	29	520 (408-700)	33	570 (390-700)	41	0.8942	0.6741	0.6884	0.9762
Blood basophils, cells/μl	0 (0-100)	29	30 (0-100)	33	50 (5-100)	41	0.0861	0.3328	**0.0200**	0.3523
[4]Sputum eosinophils counts, %	4.4 (0.7-20.0)	12	2.1 (0.8-9.5)	18	6.0 (0.4-32.9)	20	0.8398	0.7230	0.9009	0.5775
Sputum neutrophil counts, %	44.9±4.8	12	53.6±5.6	18	58.0±6.4	20	0.3506	0.2811	0.1626	0.6090
Sputum macrophage counts, %	42.5 (26.7-55.4)	12	39.1 (16.8-63.4)	18	17.8 (8.3-30.5)	20	**0.0228**	0.8128	**0.0055**	0.0545
Sputum lymphocyte counts, %	0.8 (0.2-1.4)	12	1.1 (0.5-2.2)	18	0.9 (0.4-1.6)	20	0.4169	0.2312	0.5321	0.3963
Sputum mast cell counts, %	0.0 (0.0-0.0)	12	0.0 (0.0-0.04)	18	0.0 (0.0-0.0)	20	0.5834	0.3021	0.4241	0.6641

[1]Urinary glucocorticoid metabolites were measured in 53 out of 55 severe asthma participants on OCS to confirm adherence with OCS therapy. This was confirmed in 27 out of 53 severe asthma participants. [2]Atopy was defined based on results of a serum IgE assay. [3]Skin prick tests to common aeroallergens were performed. [4]Sputum cell counts were expressed as a percent of non-squamous cells.

Data for continuous variables are presented as mean ± sem or median (interquartile range); categorical variables are expressed as numbers or percentages. Normality of variables was tested using D'Agostino and Pearson omnibus normality test. Between-group comparisons were performed using ANOVA with Tukey's multiple comparison test for normally distributed data or the Kruskal-Wallis test with Dunn's multiple comparison test for non-parametric data. A chi-square test was used for multiple comparisons of categorical variables. If the Chi-square test was positive, a Fisher exact test was used for pairwise comparisons of categorical variables. Bold indicates significant p values (<0.05).

Definition of abbreviations: ACQ, Asthma Control Questionnaire mean of 1-5; AQLQ, Asthma Quality of Life Questionnaire total mean; b.i.d., twice daily; BMI, body mass index; ESS, Epworth sleepiness scale; $FEF_{25\%-75\%}$, forced expiratory flow at 25%-75% of forced vital capacity, F_ENO, fractional exhaled nitric oxide; HADS, hospital anxiety and depression scale; hsCRP, high-sensitivity C-reactive protein; ICS, inhaled corticosteroids; IL, interleukin; IQR, interquartile range; LABA, long-acting β_2-adrenergic receptor agonists; OCS, oral corticosteroids; ppb, parts per billion; PEF, peak expiratory flow; RV, residual volume; sGaw. specific airway conductance; SNOT 20, sino-nasal outcomes test; TLC, total lung capacity; TNF, tumor necrosis factor.

Supplementary Table S7. Values of HRCT parameters used for clustering across radiomultiomics-associated clusters (RACs) and healthy control subject group*.

	RAC1		RAC2		RAC3		HS		ANOVA or Kruskal-Wallis P value	RAC1 vs RAC2	RAC1 vs RAC3	RAC2 vs RAC3	RAC1 vs HS	RAC2 vs HS	RAC3 vs HS
	Mean ± SEM or median (IQR)	N	Mean ± SEM or median (IQR)	n	Mean ± SEM or median (IQR)	n	Mean ± SEM or median (IQR)	n							
Participants, n		30		34		41		15							
Inspiratory Body Surface Area (Mostseller formula)	2.12 (1.96-2.31)	30	1.98 (1.80-2.05)	34	1.94 (1.84-2.07)	41	2.01 (1.79-2.08)	15	**0.0047**	**<0.01**	**<0.05**	0.6547	**0.0200**	0.8427	0.9089s
Inspiratory adjustedCT lung volume, mm^3	4271.0 (3760.0-4842.0)	29	5553.0 (4990.0-6345.0)	34	6556.0 (5591.0-7448.0)	41	5239.0 (4613.0-6957.0)	15	**<0.0001**	**<0.001**	**<0.0001**	**0.0028**	**<0.01**	0.9464	0.0805
Inspiratory LAA -856 HU, %	41.0±2.1	29	63.4±1.5	34	72.9±1.1	41	63.2±3.7	15	**<0.0001**	**<0.0001**	**<0.0001**	**<0.001**	**<0.0001**	0.9618	**<0.01**
Inspiratory LAA -950 HU, %	5.7 (3.6-9.5)	29	8.2 (6.0-10.4)	34	13.8 (10.3-17.9)	41	7.2 (3.8-12.8)	15	**<0.0001**	**0.0384**	**<0.0001**	**<0.001**	0.3500	0.6665	<0.01
Inspiratory LA mean BSA, mm^2/m^2	9.0 (7.5-10.4)	29	13.2 (11.2-14.2)	30	11.0 (8.9-13.3)	41	11.9 (9.1-14.5)	14	**<0.0001**	**<0.0001**	**<0.01**	**<0.05**	**<0.01**	0.2943	0.3625
Inspiratory LA median, mm^2	16.7±0.8	29	21.7±0.8	30	18.0±0.7	41	20.1±1.4	14	**0.0001**	**<0.001**	0.1938	**<0.01**	**<0.05**	0.5161	0.0657
Inspiratory MLD SD	168.4 (161.1-176.9)	29	150.7 (143.2-161.9)	34	142.9 (137.7-150.4)	41	145.8 (140.5-154.9)	15	**<0.0001**	**<0.001**	**<0.0001**	**<0.05**	**<0.0001**	0.1362	0.3210

Inspiratory percentile 15, HU	914.9±4.4	29	934.6±2.1	34	947.8±1.8	41	929.9±4.8	15	**<0.0001**	**<0.0001**	**<0.0001**	**<0.01**	**<0.05**	0.3050	**<0.01**
Inspiratory TA mean, mm^2	47.4±1.9	30	61.7±2.1	33	50.8±1.5	41	53.9±3.6	14	**<0.0001**	**<0.0001**	0.1534	**<0.001**	0.0851	0.0580	0.3460
Inspiratory TA mean BSA, mm^2/m^2	22.9 (19.2-25.4)	30	31.3 (28.3-34.5)	33	25.4 (22.8-30.4)	41	25.7 (22.3-33.4)	14	**<0.0001**	**<0.0001**	**<0.05**	**<0.001**	**<0.05**	0.0628	0.4265
Inspiratory WA mean BSA, mm^2/m^2	14.1±0.4	29	18.9±0.5	30	15.7±0.4	41	16.6±0.8	14	**<0.0001**	**<0.0001**	0.2800	**<0.0001**	**<0.05**	**<0.05**	0.1642
Inspiratory WA mean, mm^2	29.2 (24.8-33.5)	29	33.6 (30.6)-39.2)	30	28.2 (25.3-32.0)	41	29.1 (25.8-34.9)	14	**0.0003**	**<0.01**	0.9483	**<0.001**	0.6338	**0.0258**	0.6252
Expiratory adjusted CT lung volume, mm^3	2195.0 (2010.0-2555.0)	28	2727.0 (2240.0-3128.0)	33	3663.0 (2926.0-4747.0)	39	2521.0 (2104.0-2918.)	15	**<0.0001**	0.0139	**<0.0001**	**<0.001**	0.3172	0.2375	**<0.001**
Expiratory LAA -856 HU, %	9.8±1.4	28	12.5±1.7	33	31.8±2.6	38	6.3±1.4	15	**<0.0001**	0.4137	**<0.0001**	**<0.0001**	0.1102	**0.0268**	**<0.0001**
Expiratory LA mean BSA, mm^2/m^2	7.0 (6.0-8.3)	18	9.5 (8.0-11.0)	27	8.1 (6.4-9.6)	34	8.4 (6.6-10.0)	14	**0.0049**	**<0.01**	0.1807	**0.0081**	0.1446	0.1330	0.6126
Expiratory LA median, mm^2	12.9 (10.1-14.7)	18	16.3 (14.3-18.7)	27	12.8 (10.9-15.3)	34	14.3 (12.3-16.7)	14	**0.0006**	**<0.01**	0.8037	**<0.01**	0.0908	0.1065	0.1791
Expiratory MLD SD	193.9 (181.4-201.9)	28	183.4 (171.3-199.2)	33	173.0 (166.3-177.5)	39	178.7 (169.3-194.1)	15	**<0.0001**	0.1034	**<0.0001**	**<0.01**	**0.0242**	0.4602	0.0691
Expiratory Pct WA, %	69.0 (66.3-73.1)	18	66.5 (64.5-69.0)	27	66.2 (64.8-68.5)	34	65.0 (63.1-69.0)	14	**0.0224**	**0.0274**	**0.0117**	0.7539	**<0.05**	0.2645	0.3222
Expiratory TA SD	15.0 (10.2-26.9)	18	15.5 (11.4-22.3)	27	16.1 (12.3-23.4)	34	12.5 (10.9-18.9)	14	0.6166	0.9320	0.9231	0.5653	0.3518	0.3143	0.2152

Expiratory TA mean, mm^2	49.4 (42.0-59.3)	28	55.9 (44.7-68.1)	33	45.8 (36.9-56.2)	40	45.8 (40.0-56.5)	15	**0.0252**	0.0602	0.2502	**<0.05**	0.3509	0.0667	0.7708
Expiratory TA mean BSA, mm^2/m^2	23.8 (21.3-26.4)	28	28.9 (25.0-35.8)	33	23.1 (19.1-28.4)	40	23.8 (21.6-27.7)	15	**0.0021**	**<0.05**	0.8943	<0.01	0.6753	**0.0330**	0.5177
Expiratory WA mean BSA, mm^2/m^2	13.9 (11.8-17.5)	18	18.6 (15.3-19.1)	27	12.9 (11.8-15.7)	34	14.0 (13.1-15.4)	14	**<0.0001**	**<0.05**	0.4615	**<0.0001**	0.6611	**0.0032**	0.1943

*Only individuals with asthma were included in the cluster analysis. Data from healthy subjects (HS) are shown as a control group.

Data for continuous variables are presented as mean±sem or median (interquartile range); categorical variables are expressed as numbers or percentages. Normality of variables was tested using D'Agostino and Pearson omnibus normality test. Between-group comparisons were performed using ANOVA with Tukey's multiple comparison test for normally distributed data or the Kruskal-Wallis test with Dunn's multiple comparison test for non-parametric data. A chi-square test was used for multiple comparisons of categorical variables. If the Chi-square test was positive, a Fisher exact test was used for pairwise comparisons of categorical variables. Bold indicates significant p values (<0.05).

Definition of abbreviations: Adjusted CT lung volume, CT lung volume adjusted for the phantom density rods; BSA, body surface area; HS, healthy subjects; HU, Hounsfield Units; LA mean BSA, lumen area average of mean values of right and left lung segmental bronchi standardized by BSA; LA median, lumen area average of median values of right and left lung segmental bronchi; LAA -856 HU, percent of low attenuation area with CT number lower than -856 HU; LAA -950 HU, percent of low attenuation area with CT number lower than –950 HU; MLD SD, mean lung density standard deviation of CT number frequency distribution; Pct WA, wall area as a percent of total area; Percentile 15, 15th percentile of the cumulative frequency distribution of CT numbers; TA mean, total area average of mean values of right and left lung segmental bronchi; TA mean BSA, total area average of mean values of right and left lung segmental bronchi standardized by BSA; TA SD, total area standard deviation of mean values of right and left lung segmental bronchi; WA mean, wall area average of mean values of right and left lung segmental bronchi; WA mean BSA, wall area average of mean values of right and left lung segmental bronchi standardized by BSA.

Supplementary Table S8. Values of quantitative HRCT parameters in study group participants classified on the basis of the degree of asthma severity

	San		SAs/ex-		MMA		HS		Overall P value
	Mean ± SEM or median (IQR)	n	Mean ± SEM or median (IQR)	n	Mean ± SEM or median (IQR)	n	Mean ± SEM or median (IQR)	n	
Participants, n		73		20		12		15	
Inspiratory Body Surface Area (Mostseller formula)	2.0±0.02	73	2.0±0.05	20	2.0±0.07	12	2.0±0.07	15	ns
Inspiratory adjusted CT lung volume, mm^3	5489.0±158.0	72	5738.0±293.6	20	6566.0±311.6	12	5830.0±388.5	15	ns
Inspiratory LAA -856 HU, %	59.2±2.0	72	62.1±3.2	20	69.5±2.5	12	63.2±3.7	15	ns
Inspiratory LAA -950 HU, %	10.1±0.7	72	12.1±1.4	20	9.7±1.5	12	8.3±1.3		s
Inspiratory LA mean BSA, mm^2/m^2	[a]10.4 (8.6-12.5)	70	12.0 (10.1-13.2)	19	[a]13.6 (11.0-15.6)	11	11.9 (9.1-14.5)	14	**0.0116**
Inspiratory LA median, mm^2	18.1±0.6	70	19.1±0.9	19	21.9±1.4	11	20.7±1.4	14	[b]**0.0490**
Inspiratory MLD SD	155.0±1.7	72	156.1±3.6	20	147.2±2.4	12	147.8±2.8	15	ns
Inspiratory percentile 15, HU	-932.2±2.6	72	-940.3±4.0	20	-936.9±3.7	12	-929.9±4.8	15	ns
Inspiratory TA mean, mm^2	51.2 (43.0-57.8)	72	56.6 (43.8-62.0)	20	54.6 (52.1-62.5)	12	48.9 (44.0-62.6)	14	ns
Inspiratory TA mean BSA, mm^2/m^2	25.2 (22.5-30.4)	72	26.3 (22.7-31.1)	20	28.5 (25.6-34.9)	12	25.7 (22.3-33.4)	14	Ns
Inspiratory WA mean BSA, mm^2/m^2	15.9±0.4	70	16.6±0.8	19	17.5±0.8	11	16.6±0.8	14	ns
Inspiratory WA mean, mm^2	29.8 (35.7-33.6)	70	32.6 (25.7-35.1)	19	33.2 (29.9-35.5)	11	29.1 (25.8-34.9)	14	ns
Expiratory adjusted CT lung volume, mm^3	2829 (2195-3687)	69	2925 (2532-3766)	19	2646 (2108-3169)	12	2521 (2104.2918)	15	ns
Expiratory LAA -856 HU, %	[c]15.5 (6.1-27.0)	69	[a,d]16.9 (11.7-30.4)	19	[a]4.4 (0.7-23.5)	11	[c,d]4.1 (2.3-10.5)	15	**0.0003**
Expiratory LA mean BSA, mm^2/m^2	8.1 (6.5-10.1)	57	7.9 (6.5-8.4)	13	8.9 (8.7-1.1)	9	8.4 (6.6-1.0)	14	ns
Expiratory LA median, mm^2	13.6 (11.2-16.7)	57	12.9 (11.6-15.2)	13	15.7 (14.8-17.3)	9	14.3 (12.3-16.7)	14	ns
Expiratory MLD SD	180.5 (167.7-193.9)	69	183.7 (174.3-202.1)	19	183.5 (177.0-190.2)	12	178.7 (169.3-194.1)	15	ns
Expiratory Pct WA, %	67.1 (64.8-70.1)	57	67.3 (65.4-69.1)	13	65.8 (64.5-67.6)	9	65.0 (63.1-69.0)	14	ns
Expiratory TA SD	15.7 (11.4-23.3)	57	14.4 (12.2-23.9)	13	15.5 (11.7-19.2)	9	12.5 (10.9-18.9)	14	ns
Expiratory TA mean, mm^2	50.5 (41.2-60.4)	72	48.8 (40.3-61.3)	18	51.6 (44.3-63.1)	11	45.8 (40.0-56.5)	15	ns
Expiratory TA mean BSA, mm^2/m^2	25.0 (20.7-30.3)	72	23.3 (19.6-30.3)	18	27.1 (24.7-29.6)	11	23.8 (21.6-27.7)	15	ns
Expiratory WA mean BSA, mm^2/m^2	14.9 (12.1-17.7)	57	12.7 (12.1-16.7)	13	16.4 (14.6-18.2)	9	14.0 (13.1-15.4)	14	ns

Data for continuous variables are presented as mean±sem or median (interquartile range); categorical variables are expressed as numbers or percentages. Normality of variables was tested using D'Agostino and Pearson omnibus normality test. Between-group comparisons were performed using ANOVA with Tukey's multiple comparison test for normally distributed data or the Kruskal-Wallis test with Dunn's multiple comparison test for non-parametric data. A chi-square test was used for multiple comparisons of categorical variables. If the Chi-square test was positive, a Fisher exact test was used for pairwise comparisons of categorical variables. Bold indicates significant p values (<0.05).

[a]P<0.05; [b]Dunn's multiple comparison test negative; [c]P<0.01; [d]P<0.01.

Definition of abbreviations: Adjusted CT lung volume, CT lung volume adjusted for the phantom density rods; BSA, body surface area; HU, Hounsfield Units; LA mean BSA, lumen area average of mean values of right and left lung segmental bronchi standardized by BSA; LA median, lumen area average of median values of right and left lung segmental bronchi; LAA -856 HU, percent of low attenuation area with CT numbers lower than -856 HU; LAA -950 HU, percent of low attenuation area with CT numbers lower than –950 HU; MLD SD, mean lung density standard deviation of CT number frequency distribution; Pct WA, wall area as a percent of total area; Percentile 15, 15th percentile of the cumulative frequency distribution of CT numbers; TA mean, total area average of mean values of right and left lung segmental bronchi; TA mean BSA, total area average of mean values of right and left lung segmental bronchi standardized by BSA; TA SD, total area standard deviation of mean values of right and left lung segmental bronchi; WA mean, wall area average of mean values of right and left lung segmental bronchi; WA mean BSA, wall area average of mean values of right and left lung segmental bronchi standardized by BSA.

Supplementary Table S9. Quantitative CT parameters: comparisons between radiomic-associated cluster (RAC) 1 and healthy control group

	Healthy subjects		RAC1		P value
	Mean ± SEM or median (IQR)	n	Mean ± SEM or Median (IQR)	n	
Participants, n		15		30	
Inspiratory Body Surface Area (Mostseller formula)	1.95±0.07	15	2.12±0.05	30	**0.0346**
Inspiratory adjusted CT lung volume, mm^3	5830±388.5	15	4354±173.6	29	**0.0002**
Inspiratory LAA -856 HU, %	63.3±3.7	15	41.0 ±2.1	29	**<0.0001**
Inspiratory LAA -950 HU, %	7.2 (3.8-12.8)	15	5.7 (3.6-9.5)	29	ns
Inspiratory LA mean BSA, mm^2/m^2	12.1±0.9	14	8.8±0.4	29	**0.0003**
Inspiratory LA median, mm^2	20.7±1.4	14	16.7±0.8	29	**0.0131**
Inspiratory MLD SD	147.8±2.8	15	169.1±1.9	29	**<0.0001**
Inspiratory percentile 15, HU	-929.9±4.8	15	-914.9±4.4	29	**0.0388**
Inspiratory TA mean, mm^2	53.9±3.5	14	47.4±1.9	30	ns
Inspiratory TA mean BSA, mm^2/m^2	27.8±1.6	14	22.3±0.7	30	**0.0009**
Inspiratory WA mean BSA, mm^2/m^2	16.6±0.8	14	14.1±0.4	29	**0.0041**
Inspiratory WA mean, mm^2	29.1 (25.8-34.9)	14	29.2 (24.8-33.5)	29	ns
Expiratory adjusted CT lung volume, mm^3	2512±167.8	15	2596±170.9	28	ns
Expiratory LAA -856 HU, %	6.3±1.4	15	9.8±1.4	28	ns
Expiratory LA mean BSA, mm^2/m^2	8.4 (6.6-10.0)	14	7.0 (6.0-8.3)	18	ns
Expiratory LA median, mm^2	14.3 (12.3-16.7)	14	12.8±0.5	18	ns
Expiratory MLD SD	178.7 (169.3-194.1)	15	193.9 (181.4-201.9)	28	**0.0242**
Expiratory Pct WA, %	65.6±0.9	14	69.9±1.0	18	**0.0058**
Expiratory TA SD	12.5 (10.9-18.9)	14	15.0 (10.2-26.9)	18	ns
Expiratory TA mean, mm^2	45.8 (40.0-56.5)	15	49.4 (42.0-59.3)	28	ns
Expiratory TA mean BSA, mm^2/m^2	23.8 (21.6-27.7)	15	23.8 (21.3-26.4)	28	ns
Expiratory WA mean BSA, mm^2/m^2	14.0 (13.1-15.4)	14	13.9 (11.8-17.5)	18	ns

Data for continuous variables are presented as mean±sem or median (interquartile range). Normality of variables was tested using D'Agostino and Pearson omnibus normality test. Between-group comparisons were performed using unpaired t test for normally distributed data or the Mann-Whitney test for non-parametric data. Bold indicates significant p values (<0.05).

Definition of abbreviations: Adjusted CT lung volume, CT lung volume adjusted for the phantom density rods; BSA, body surface area; HU, Hounsfield Units; LA mean BSA, lumen area average of mean values of right and left lung segments standardized by BSA; LA median, lumen area average of median values of right and left lung segmental bronchi; LAA -856 HU, percent of low attenuation area with CT number lower than -856 HU; LAA -950 HU, percent of low attenuation area with CT number lower than –950 HU; MLD SD, mean lung density standard deviation of CT number frequency distribution; Pct WA, wall area as a percent of total area; Percentile 15, 15th percentile of the cumulative frequency distribution of CT numbers; TA mean, total area average of mean values of right and left lung segments; TA mean BSA, total area average of mean values of right and left lung segments standardized by BSA; TA SD, total area standard deviation of mean values of right and left lung segments; WA mean, wall area average of mean values of right and left lung segments; WA mean BSA, wall area average of mean values of right and left lung segmental bronchi standardized by BSA.

Supplementary Table S10. Quantitative CT parameters: comparisons between radiomic-associated cluster (RAC) 2 and healthy control group

	Healthy subjects		RAC2		P value
	Mean ± SEM or median (IQR)	n	Mean ± SEM or Median (IQR)	n	
Participants, n		15		34	
Inspiratory Body Surface Area (Mostseller formula)	1.95±0.07	15	1.93±0.04	34	ns
Inspiratory adjusted CT lung volume, mm^3	5830±388.5	15	5727±160.0	34	ns
Inspiratory LAA -856 HU, %	63.3±3.7	15	63.4 ±1.5	34	ns
Inspiratory LAA -950 HU, %	7.2 (3.8-12.8)	15	8.2 (6.0-10.4)	34	ns
Inspiratory LA mean BSA, mm^2/m^2	11.9 (9.1-14.5)	14	13.2 (11.2-14.2)	30	ns
Inspiratory LA median, mm^2	20.7±1.4	14	21.7±0.8	30	ns
Inspiratory MLD SD	145.8 (140.5-154.9)	15	150.7 (143.2-161.9)	34	ns
Inspiratory percentile 15, HU	-929.9±4.8	15	-934.5±2.1	34	ns
Inspiratory TA mean, mm^2	53.9±3.5	14	61.7±2.1	33	ns
Inspiratory TA mean BSA, mm^2/m^2	25.7 (22.3-33.4)	14	31.3 (28.3-34.5)	33	ns
Inspiratory WA mean BSA, mm^2/m^2	16.6±0.8	14	18.9±0.5	30	**0.0181**
Inspiratory WA mean, mm^2	30.4±1.7	14	34.8±1.1	30	**0.0312**
Expiratory adjusted CT lung volume, mm^3	2521.0 (2104.0-2918.0)	15	2727.0 (2240.0-3128.0)	33	ns
Expiratory LAA -856 HU, %	6.3±1.4	15	12.5±1.7	33	**0.0268**
Expiratory LA mean BSA, mm^2/m^2	8.4 (6.6-10.0)	14	9.5 (8.0-11.0)	27	ns
Expiratory LA median, mm^2	14.3 (12.3-16.7)	14	16.3 (14.3-18.7)	27	ns
Expiratory MLD SD	178.7 (169.3-194.1)	15	183.4 (171.3-199.2)	33	ns
Expiratory Pct WA, %	65.6±0.9	14	66.9±0.7	27	ns
Expiratory TA SD	12.5 (10.9-18.9)	14	15.5 (11.4-22.3)	27	ns
Expiratory TA mean, mm^2	45.8 (40.0-56.5)	15	55.9 (44.7-68.1)	33	ns
Expiratory TA mean BSA, mm^2/m^2	23.8 (21.6-27.7)	15	28.9 (25.0-35.8)	33	**0.0330**
Expiratory WA mean BSA, mm^2/m^2	14.0 (13.1-15.4)	14	18.6 (15.3-19.1	27	**0.0032**

Data for continuous variables are presented as mean±sem or median (interquartile range). Normality of variables was tested using D'Agostino and Pearson omnibus normality test. Between-group comparisons were performed using unpaired t test for normally distributed data or the Mann-Whitney test for non-parametric data. Bold indicates significant p values (<0.05).

Definition of abbreviations: Adjusted CT lung volume, CT lung volume adjusted for the phantom density rods; BSA, body surface area; HU, Hounsfield Units; LA mean BSA, lumen area average of mean values of right and left lung segments standardized by BSA; LA median, lumen area average of median values of right and left lung segmental bronchi; LAA -856 HU, percent of low attenuation area with CT number lower than -856 HU; LAA -950 HU, percent of low attenuation area with CT number lower than –950 HU; MLD SD, mean lung density standard deviation of CT number frequency distribution; Pct WA, wall area as a percent of total area; Percentile 15, 15th percentile of the cumulative frequency distribution of CT numbers; TA mean, total area average of mean values of right and left lung segments; TA mean BSA, total area average of mean values of right and left lung segments standardized by BSA; TA SD, total area standard deviation of mean values of right and left lung segments; WA mean, wall area average of mean values of right and left

lung segments; WA mean BSA, wall area average of mean values of right and left lung segmental bronchi standardized by BSA.

Supplementary Table S11. Quantitative CT parameters: comparisons between radiomic-associated cluster (RAC) 3 and healthy control group

	Healthy subjects		RAC3		P value
	Mean ± SEM or median (IQR)	N	Mean ± SEM or Median (IQR)	n	
Participants, n		15		41	
Inspiratory Body Surface Area (Mostseller formula)	2.01 (1.79-2.08)	15	1.94 (1.84-2.07)	41	ns
Inspiratory adjusted CT lung volume, mm^3	5239.0 (4613.0-6957.0)	15	6556.0 (5591.0-7448.0)	41	ns
Inspiratory LAA -856 HU, %	63.3±3.7	15	72.9±1.1	41	**0.0012**
Inspiratory LAA -950 HU, %	7.2 (3.8-12.8)	15	13.8 (10.3-17.9	34	**0.0011**
Inspiratory LA mean BSA, mm^2/m^2	12.1±0.9	14	11.1±0.4	41	ns
Inspiratory LA median, mm^2	20.7±1.4	14	18.0±0.7	41	ns
Inspiratory MLD SD	147.8±2.8	15	144.3±1.3	41	ns
Inspiratory percentile 15, HU	-929.9±4.8	15	-947.8±1.8	41	**<0.0001**
Inspiratory TA mean, mm^2	53.9±3.5	14	50.8±1.5	41	ns
Inspiratory TA mean BSA, mm^2/m^2	27.8±1.6	14	26.0±0.7	41	ns
Inspiratory WA mean BSA, mm^2/m^2	16.6±0.8	14	15.7±0.4	41	ns
Inspiratory WA mean, mm^2	30.4±1.7	14	29.2±0.8	41	ns
Expiratory adjusted CT lung volume, mm^3	2512.0±167.8	15	3843±172.4	39	**<0.0001**
Expiratory LAA -856 HU, %	6.3±1.4	15	31.8±2.6	38	**<0.0001**
Expiratory LA mean BSA, mm^2/m^2	8.4 (6.6-10.0)	14	8.1 (6.4-9.6)	34	ns
Expiratory LA median, mm^2	14.3 (12.3-16.7)	14	12.8 (10.9-15.3)	34	ns
Expiratory MLD SD	178.7 (169.3-194.1)	15	173.0 (166.3-177.5)	39	ns
Expiratory Pct WA, %	65.0 (63.1-69.0)	14	66.2 (64.8-68.5)	34	ns
Expiratory TA SD	12.5 (10.9-18.9)	14	15.5 (11.4-22.3)	27	ns
Expiratory TA mean, mm^2	45.8 (40.0-56.5)	15	45.8 (36.9-56.2)	40	ns
Expiratory TA mean BSA, mm^2/m^2	23.8 (21.6-27.7)	15	23.1 (19.1-28.4)	40	ns
Expiratory WA mean BSA, mm^2/m^2	14.0 (13.1-15.4)	14	12.9 (11.8-15.7)	34	ns

Data for continuous variables are presented as mean±sem or median (interquartile range). Normality of variables was tested using D'Agostino and Pearson omnibus normality test. Between-group comparisons were performed using unpaired t test for normally distributed data or the Mann-Whitney test for non-parametric data. Bold indicates significant p values (<0.05).

Definition of abbreviations: Adjusted CT lung volume, CT lung volume adjusted for the phantom density rods; BSA, body surface area; HU, Hounsfield Units; LA mean BSA, lumen area average of mean values of right and left lung segments standardized by BSA; LA median, lumen area average of median values of right and left lung segmental bronchi; LAA -856 HU, percent of low attenuation area with CT number lower than -856 HU; LAA -950 HU, percent of low attenuation area with CT number lower than –950 HU; MLD SD, mean lung density standard deviation of CT number frequency distribution; Pct WA, wall area as a percent of total area; Percentile 15, 15th percentile of the cumulative frequency distribution of CT numbers; TA mean, total area average of mean values of right and left lung segments; TA mean BSA, total area average of mean values of right and left lung segments standardized by BSA; TA SD, total area standard deviation of mean values of right and left lung segments; WA mean, wall area average of mean values of right and left lung segments; WA mean BSA, wall area average of mean values of right and left lung segmental bronchi standardized by BSA..

Supplementary Table S12. Association Between Smoking Status and RACs Among U-BIOPRED Asthmatics.

Cohort	Smoking status	RAC1	RAC2	RAC3	P value
U-BIOPRED	**Current Smoker**	0(0.0)	2(5.9)	5(12.2)	0.2913
	**Ex-smoker**	9(30.0)	7(20.6)	11(26.8)	
	**Non-smoker**	21(70.0)	25(73.5)	25(61.0)	
ATLANTIS	**Current Smoker**	0 (0%)	0 (0%)	1 (4.0%)	0.2
	**Ex-smoker**	6 (46%)	10 (23%)	5 (20%)	
	**Non-smoker**	7 (54%)	33 (77%)	19 (76%)	

Supplementary Table S13. Radiological characteristics of qCT clusters in the ATLANTIS external replication cohort

	qCT 1		qCT 2		qCT 3		ANOVA or Kruskal-Wallis P value	qCT 1 vs qCT 2	qCT 1 vs qCT 3	qCT2 vs qCT 3
	Mean ± SEM or median (IQR)	n	Mean ± SEM or median (IQR)	n	Mean ± SEM or median (IQR)	n				
Participants, n		16		49		32				
Inspiratory Body Surface Area (Mostseller formula)	2.18 (2.07-2.22)	16	1.76 (1.67-1.95)	49	1.92 (1.81-2.14)	32	**<0.001**	**<0.001**	**0.006**	**0.007**
Inspiratory adjusted CT lung volume, mm^3	4418 (3110-4926)	16	5244 (4576-6235)	49	5469 (4312-6061)	32	**0.009**	**0.006**	**0.004**	ns
Inspiratory LAA -856 HU, %	42.3 (27.3-46.3)	16	67.4 (58.4-71.6)	49	72.1 (63.2-77.2)	32	**<0.001**	**<0.001**	**<0.001**	ns
Inspiratory LAA -950 HU, %	2.0 (0.7 - 3.1)	16	3.7 (1.8 - 6.3)	49	6.4 (3.9-12.6)	32	**<0.001**	**0.018**	**<0.001**	**0.003**
Inspiratory LA mean BSA, mm^2/m^2	8.5 (6.7-9.6)	16	11.7 (10.8-13.2)	49	8.1 (6.5- 9.2)	32	**<0.001**	**<0.001**	ns	**<0.001**
Inspiratory LA median, mm^2	17.8±0.9	16	22.0±0.8	49	15.8±0.7	32	**<0.001**	**0.004**	ns	**<0.001**
Inspiratory percentile 15, HU	-893 (-910-883)	16	-922 (-933-912)	49	-933 (-945-923)	32	**<0.001**	**<0.001**	<0.001	**0.01**
Inspiratory TA mean, mm^2	52.2 (45.6-55.2)	16	59.1 (51.9-65.7)	49	44.1 (40.6-50.0)	32	**<0.001**	**0.036**	ns	**<0.001**
Inspiratory TA mean BSA, mm^2/m^2	24.7 (20.2-26.1)	16	32.1 (29.5-35.0)	49	24.0 (20.2-26.5)	32	**<0.001**	**<0.001**	ns	**<0.001**
Inspiratory WA mean BSA, mm^2/m^2	16.1 (13.3-17.0)	16	19.5 (17.8-21.3)	49	15.4 (13.1-16.6)	32	**<0.001**	**<0.001**	ns	**<0.001**
Inspiratory WA mean, mm^2	33.7 (29.2-35.7)	16	36.3 (32.8-40.6)	49	28.5 (26.5-32.9)	32	**<0.001**	ns	ns	**<0.001**
Expiratory adjusted CT lung volume, mm^3	2459 (2121-2865)	16	2766 (2245-3167)	49	2836 (2395-3522)	32	ns	ns	ns	ns
Expiratory LAA -856 HU, %	5.3 (3.9 - 10.2)	16	13.8 (4.1-18.6)	49	23.3 (12.0-40.8)	32	**<0.001**	ns	**<0.001**	**0.002**
Expiratory LA mean BSA, mm^2/m^2	6.1 (4.6 - 6.6)	16	8.3 (7.2-9.1)	49	4.8 (4.0-6.7)	32	**<0.001**	**<0.001**	ns	**<0.001**
Expiratory LA median, mm^2	12.0 (9.5-14.3)	16	15.1 (13.3-16.8)	49	10.4 (8.0-13.1)	32	**<0.001**	**0.007**	ns	**<0.001**
Expiratory Pct WA, %	25.9 (24.0-29.1)	16	28.7 (26.2-33.4)	49	23.0 (20.7-26.6)	32	**<0.001**	ns	ns	**<0.001**
Expiratory TA mean, mm^2	38.3 (33.7-46.5)	16	44.7 (39.8-50.6)	49	35.4 (28.8-39.2)	32	**<0.001**	**0.009**	ns	**<0.001**
Expiratory TA mean BSA, mm^2/m^2	18.5 (16.1-19.7)	16	25.1 (22.8-28.1)	49	16.6 (15.5-20.3)	32	**<0.001**	**<0.001**	ns	**<0.001**
Expiratory WA mean BSA, mm^2/m^2	12.4 (11.3-13.5)	16	16.1 (14.9-18.1)	49	11.6 (10.6-13.5)	32	**<0.001**	**<0.001**	ns	**<0.001**

Data for continuous variables are presented as median (interquartile range). Between-group comparisons were performed using the Kruskal-Wallis test with Dunn's multiple comparison test. Bold indicates significant p values (<0.05).

Definition of abbreviations: Adjusted CT lung volume, CT lung volume adjusted for the phantom density rods; BSA, body surface area; HU, Hounsfield Units; LA mean BSA, lumen area average of mean values of right and left lung segmental bronchi standardized by BSA; IQR, interquartile range; LA median, lumen area average of median values of right and left lung segmental bronchi; LAA -856 HU, percent of low attenuation area with CT number lower than -856 HU; LAA -950 HU, percent of low attenuation area with CT number lower than – 950 HU; Pct WA, wall area as a percent of total area; Percentile 15, 15th percentile of the cumulative frequency distribution of CT numbers; qCT, quantitative CT; TA mean, total area average of mean values of right and left lung segmental bronchi; TA mean BSA, total area average of mean values of right and left lung segmental bronchi standardized by BSA; WA mean, wall area average of mean values of right and left lung segments; WA mean BSA, wall area average of mean values of right and left lung segmental bronchi standardized by BSA.

Supplementary Table S14. Radiological characteristics of qCT clusters in the U-BIOPRED discovery cohort and in the ATLANTIS external replication cohort.

	U-BIOPRED			ATLANTIS			U-BIOPRED vs ATLANTIS		
	qCT 1 (n=30)	qCT 2 (n=34)	qCT 3 (n=41)	qCT 1 (n=16)	qCT 2 (n=49)	qCT 3 (n=32)	qCT 1 P value	qCT 2 P value	qCT 3 P value
	Mean ± SEM or median (IQR)	Mean ± SEM or median (IQR)	Mean ± SEM or median (IQR)	Mean ± SEM or median (IQR)	Mean ± SEM or median (IQR)	Mean ± SEM or median (IQR)			
Inspiratory Body Surface Area (Mostseller formula)	2.12 (1.96-2.31)	1.98 (1.80-2.05)	1.94 (1.84-2.07)	2.18 (0.15)	1.76 (0.28)	1.92 (0.34	ns	**0.007**	ns
Inspiratory adjusted CT lung volume, mm^3	4271 (3760-4842)	5553 (4990-6345)	6556 (5591-7448	4418 (3110-4926)	5244 (4576-6235)	5469 (4312-6061)	ns	ns	**0.002**
Inspiratory LAA -856 HU, %	41.0±2.1	63.4±1.5	72.9±1.1	42.3 (27.3-46.3)	67.4 (58.4-71.6)	72.1 (63.2-77.2)	ns	ns	ns
Inspiratory LAA -950 HU, %	5.7 (3.6-9.5)	8.2 (6.0-10.4)	13.8 (10.3-17.9)	2.0 (0.7-3.1)	3.7 (1.8-6.3)	6.4 (3.9-12.6)	**<0.001**	**<0.001**	**<0.001**
Inspiratory LA mean BSA, mm^2/m^2	9.0 (7.5-10.4)	13.2 (11.2-14.2)	11.0 (8.9-13.3)	8.5 (6.7-9.6)	11.7 (10.8-13.2)	8.1 (6.5-9.2)	ns	**0.03**	**<0.001**
Inspiratory LA median, mm^2	16.7±0.8	21.7±0.8	18.0±0.7	17.8±0.9	22.0±0.8	15.8±0.7	ns	ns	**0.023**
Inspiratory percentile 15, HU	914.9±4.4	934.6±2.1	947.8±1.8	893 (-910- 883)	-922 (-933- 912)	-933 (-945- 923)	**0.002**	**0.001**	ns
Inspiratory TA mean, mm^2	47.4±1.9	61.7±2.1	50.8±1.5	52.2 (45.6-55.2)	59.1 (51.9-65.7)	44.1 (40.6-50.0)	ns	ns	**0.026**
Inspiratory TA mean BSA, mm^2/m^2	22.9 (19.2-25.4)	31.3 (28.3-34.5)	25.4 (22.8-30.4)	24.7 (20.2-26.1)	32.1 (29.5-35.0)	24.0 (20.2-26.5)	ns	ns	**0.035**
Inspiratory WA mean BSA, mm^2/m^2	14.1±0.4	18.9±0.5	15.7±0.4	16.1 (13.3-17.0)	19.5 (17.8-21.3)	15.4 (13.1-16.6)	ns	ns	ns
Inspiratory WA mean, mm^2	29.2 (24.8-33.5)	33.6 (30.6)-39.2)	28.2 (25.3-32.0)	33.7 (29.2-35.7)	36.3 (32.8-40.6)	28.5 (26.5-32.9)	**0.02**	ns	ns
Expiratory adjusted CT lung volume, mm^3	2195 (2010-2555)	2727 (2240-3128)	3663 (2926-4747)	2459 (2121-2865)	2766 (2245-3167)	2836-(2395-3522)	ns	ns	**0.002**
Expiratory LAA -856 HU, %	9.8±1.4	12.5±1.7	31.8±2.6	5.3 (3.9 - 10.2)	13.8 (4.1-18.6)	23.3 (12.0-40.8)	ns	ns	ns
Expiratory LA mean BSA, mm^2/m^2	7.0 (6.0-8.3)	9.5 (8.0-11.0)	8.1 (6.4-9.6)	6.1 (4.6-6.6)	8.3 (7.2-9.1)	4.8 (4.0-6.7)	**<0.001**	**<0.001**	**<0.001**

Expiratory LA median, mm^2	12.9 (10.1-14.7)	16.3 (14.3-18.7)	12.8 (10.9-15.3)	12.0 (9.5-14.3)	15.1 (13.3-16.8	10.4 (8.0-13.1)	ns	ns	**0.002**
Expiratory Pct WA, %	69.0 (66.3-73.1)	66.5 (64.5-69.0)	66.2 (64.8-68.5)	25.9 (24.0-29.1)	28.7 (26.2-33.4)	23.0 (20.7-26.6)	**<0.001**	**<0.001**	**<0.001**
Expiratory TA mean, mm^2	49.4 (42.0-59.3)	55.9 (44.7-68.1)	45.8 (40.0-56.5)	38.3 (33.7-46.5)	44.7 (39.8-50.6)	35.4 (28.8-39.2)	**<0.001**	**<0.001**	**<0.001**
Expiratory TA mean BSA, mm^2/m^2	23.8 (21.3-26.4)	28.9 (25.0-35.8)	23.1 (19.1-28.4)	18.5 (16.1-19.7)	25.1 (22.8-28.1)	16.6 (15.5-20.3)	**<0.001**	**<.001**	**<0.001**
Expiratory WA mean BSA, mm^2/m^2	13.9 (11.8-17.5)	18.6 (15.3-19.1)	12.9 (11.8-15.7)	12.4 (11.3-13.5)	16.1 (14.9-18.1)	11.6 (10.6-13.5)	**0.02**	**0.007**	**0.001**

Data for continuous variables are presented as mean±sem or median (interquartile range). Normality of variables was tested using D'Agostino and Pearson omnibus normality test. Between-group comparisons were performed using ANOVA with Tukey's multiple comparison test for normally distributed data or the Kruskal-Wallis test with Dunn's multiple comparison test for non-parametric data. Bold indicates significant p values (<0.05).

Definition of abbreviations: Adjusted CT lung volume, CT lung volume adjusted for the phantom density rods; BSA, body surface area; HU, Hounsfield Units; LA mean BSA, lumen area average of mean values of right and left lung segmental bronchi standardized by BSA; IQR, interquartile range; LA median, lumen area average of median values of right and left lung segmental bronchi; LAA -856 HU, percent of low attenuation area with CT number lower than -856 HU; LAA -950 HU, percent of low attenuation area with CT number lower than –950 HU; Pct WA, wall area as a percent of total area; Percentile 15, 15th percentile of the cumulative frequency distribution of CT numbers; qCT, quantitative CT; TA mean, total area average of mean values of right and left lung segmental bronchi; TA mean BSA, total area average of mean values of right and left lung segmental bronchi standardized by BSA; WA mean, wall area average of mean values of right and left lung segments; WA BSA, wall area average of mean values of right and left lung segmental bronchi standardized by BSA.

Supplementary Table S15. Characteristics of qCT clusters in the ATLANTIS external replication cohort

	Cluster 1 (n=16)		Cluster 2 (n=49)		Cluster 3 (n=32)		P value	Cluster 1 vs cluster 2	Cluster 1 vs cluster 3	Cluster 2 vs cluster 3
	Mean ± SEM or IQR or numbers (%)	Missing values (n)	Mean ± SEM or IQR or numbers (%)	Missing values (n)	Mean ± SEM or IQR or numbers (%)	Missing values (n)				
Sex		3		6		7	ns	ns	ns	ns
Females	8 (62%)		26 (60%)		14 (56%)					
Males	5 (38%)		17 (40%)		11 (44%)					
Age, years	54 (44-60)	3	52 (42-59)	6	53 (46-59)	7	ns	ns	ns	ns
BMI, kg/m^2	33.0 (28.7-36.4)	3	26.2 (24.2-28.2)	6	27.3 (24.7-31.2)	7	**0.001**	**<0.001**	**0.037**	ns
GINA step		3		6		7				
1	0 (0%)		0 (0%)		0 (0%)					
2	1 (7.7%)		5 (12%)		2 (8%)		ns			
3	2 (15%)		6 (14%)		1 (4%)		ns			
4	8 (62%)		28 (65%)		16 (64%)		ns			
5	2 (15%)		4 (9.3%)		6 (24%)		ns			
GINA step 4-5, %	10 (77%)		32 (74.3%)		22 (88%)		ns			
Asthma exacerbations in previous 12 months, n		3		6			7			
0	10 (77%)		31 (72%)		13 (52%)		ns			
1	2 (15%)		4 (9.3%)		8 (32%)		ns			
2	1 (7.7%)		4 (9.3%)		2 (8.0%)		ns			
3	0 (0%)		1 (2.3%)		1 (4.0%)		ns			
4	0 (0%)		2 (4.7%)		1 (4.0%)		ns			
5	0 (0%)		1 (2.3%)		0 (0%)		ns			
Asthma exacerbations in previous 12 months ≥ 2, n	1 (7.7%)	3	8 (18.6%)	6	4 (16%)	7	ns			
Pre-bronchodilator FEV$_1$, L	2.6±0.2	3	2.5±0.1	6	1.9±0.2	7	**0.02**	ns	ns	**0.029**
Pre-bronchodilator FEV$_1$, % predicted value	81.6±4.4	3	82.2±3.0	6	61.7±3.5	7	**<0.001**	ns	**0.014**	**<0.001**
Pre-bronchodilator FVC, L	3.2 (2.7-3.6)	3	3.82 (3.0-4.4)	6	3.10 (2.6-4.3)	7	ns	ns	ns	ns
Pre-bronchodilator	88.0±3.9	3	97.7±2.3	6	89.3±2.7	7	**0.045**	Nn	ns	ns

FVC, % predicted value										
Pre-bronchodilator FEV_1/FVC, %	0.77±0.03	3	0.67±0.02	6	0.54±0.02	7	**<0.001**	ns	**<0.001**	**<0.001**
Pre-bronchodilator $FEF_{25\%-75\%}$, L/s	2.3 (1.4-2.7)	3	1.3 (1.0-2.4)	6	0.7 (0.5-1.0)	7	**<0.001**	ns	**<0.001**	**<0.001**
Pre-bronchodilator $FEF_{25\%-75\%}$, % predicted value	70.1 (66.4-94.1)	3	51.7 (36.4-78.9)	6	22.9 (13.0-33.7)	7	**<0.001**	ns	**<0.001**	**<0.001**
RV, L	2.08±0.12	3	2.36±0.12	9	2.51 ± 0.10	10	ns	ns	ns	ns
TLC, L	5.12 (4.43-5.42)	3	5.90 (5.03-6.63)	9	6.18 (4.86-6.61)	10	ns	ns	ns	ns
RV/TLC	0.40±0.02	3	0.39±0.02	9	0.42±0.01	10	ns	ns	ns	ns
sGaw, 1/kPa*s	1.50±0.24	3	1.30±0.11	6	0.88±0.06	7	**0.006**	ns	**0.040**	**0.034**
ICS use, at least b.i.d., %	8 (62%)	3	20 (47%)	6	11 (44%)	7	ns	ns	ns	ns
LABA use, at least b.i.d., %	6 (46%)	3	10 (23%)	6	10 (40%)	7	ns	ns	ns	ns
ACQ.6, average score	1.2 (0.5-2.2)	3	1.0 (0.5-1.8)	6	1.6 (0.7-2.3)	7	ns	ns	ns	ns
MINI, total mean score	5.5 (4.2-6.4)	3	5.7 (4.8-6.1)	6	5.1 (4.5 6.1)	7	ns	ns	ns	ns
Smoking habit		3		6		7				
Current/ex-smokers/non-smokers, n	0/6/7	3	0/10/33	6	1/5/19	7	ns	not applicable	ns	ns
F_ENO, ppb	25.0 (17.0-29.5)	5	25.0 (18.0-38.5)	10	22.0 (11.0-40.0)	11	ns	ns	ns	ns
Blood eosinophils, cells/μl	0.22 (0.11-0.29)	3	0.26 (0.12-0.50)	6	0.25 (0.20-0.40)	7	ns	ns	ns	ns
Blood neutrophils, cells/μl	4.59 (3.00-6.44)	3	3.55 (2.96-4.36)	6	4.29 (3.28-4.89)	7	ns	ns	ns	ns
Blood lymphocytes, cells/μl	1.74 (1.42-2.23)	3	1.90 (1.54-2.18)	6	1.89 (1.48-2.30)	7	ns	ns	ns	ns
Blood white cells cells/μl	7.20 (6.60-8.84)	3	6.40 (5.32-7.70)	6	6.75 (6.40-8.05)	7	ns	ns	ns	ns
Blood monocytes, cells/μl	0.50 (0.40-0.70)	3	0.47 (0.40-0.60)	6	0.55 (0.41-0.60)	7	ns	ns	ns	ns
Blood basophils, cells/μl	0.04 (0.03-0.06)	3	0.06 (0.02-0.08)	6	0.03 (0.02-0.06)	7	ns	ns	ns	ns

[4]Sputum eosinophils counts, %	0.00 (0.00-0.30)	10	1.90 (0.25-7.48)	29	2.80 (0.00-29.40)	23	ns	ns	ns	Ns
Sputum neutrophil counts, %	39.3±5.81	10	60.1±3.2	29	45.0±4.0	23	ns	ns	ns	Ns
Sputum macrophage counts, %	46±8.2	10	28.6±3.0	29	34.2±3.8	23	ns	ns	ns	Ns
Sputum lymphocyte counts, %	0.85 (0.33-1.60)	10	0.80 (0.40-1.20)	29	0.30 (0.10-0.80)	23	ns	ns	ns	Ns

Data for continuous variables are presented as mean±sem or median (interquartile range); categorical variables are expressed as numbers or percentages. Normality of variables was tested using D'Agostino and Pearson omnibus normality test. Between-group comparisons were performed using ANOVA with Tukey's multiple comparison test for normally distributed data or the Kruskal-Wallis test with Dunn's multiple comparison test for non-parametric data. A chi-square test was used for multiple comparisons of categorical variables. If the Chi-square test was positive, a Fisher exact test was used for pairwise comparisons of categorical variables. Bold indicates significant p values (<0.05).

Definition of abbreviations: ACQ, Asthma Control Questionnaire mean of 1-5; b.i.d., twice daily; BMI, body mass index; $FEF_{25\%-75\%}$, forced expiratory flow at 25%-75% of forced vital capacity, F_ENO, fractional exhaled nitric oxide; ICS, inhaled corticosteroids; LABA, long-acting β_2-adrenergic receptor agonists; OCS, oral corticosteroids; ppb, parts per billion; RV, residual volume; sGaw. specific airway conductance; TLC, total lung capacity.

Supplementary Table S16. Enrichment of molecular pathways across radiomultiomic-associated clusters (RACs).

Pathway	Omic/ Biological matrix	RAC1 median (IQR) enrichment score	RAC2 median (IQR) enrichment score	RAC3 median (IQR) enrichment score	Kruskal-Wallis P value	RAC1 vs RAC2 P value	RAC1 vs RAC3 P value	RAC2 vs RAC3 P value	Overlapping genes or proteins
Complement cascade	Proteomics Serum	0.54 (0.24-0.67)	-0.05 (-0.35-0.23)	0.07 (-0.33-0.37)	**1.3e-05**	**1e-05**	**2e-05**	0.598561	CFH, CFI, C5, C9, C1S, CFD
GPVI-mediated activation cascade	Proteomics Serum	-0.56 (-0.72-0.1)	-0.35 (-0.66-0.03)	0.27 (-0.49-0.59)	**0.00146**	0.283067	**0.001364**	**0.00539**	PTPN6, LYN, FYN, PDPK1, CLEC1B, GP6
Signaling by SCF KIT	Proteomics Serum	-0.28 (-0.5--0.07)	-0.33 (-0.52-0.02)	0.15 (-0.25-0.49)	**0.00321**	0.94312	**0.00572**	**0.00302**	PTPN6, LYN, FYN, SRC, FER, TEC, RAC1, PRKCA, PTPN11, JAK2
Regulation of KIT signalling	Proteomics Serum	-0.53 (-0.78-0.03)	-0.33 (-0.58-0.09)	0.26 (-0.38-0.64)	**0.00494**	0.12495	**0.00268**	**0.04282**	PTPN6, LYN, FYN, SRC
Complement cascade	Proteomics Serum	0.48 (0.21-0.64)	0.09 (-0.19-0.35)	-0.08 (-0.36-0.32)	**0.00015**	**0.00113**	**5e-05**	0.27836	CFI, C9, CFH, C5, CD55, C3, FCN1, CRP
Regulation of IGF transport and uptake by IGFBPs	Proteomics Serum	0.25 (-0.11-0.5)	0.05 (-0.24-0.35)	-0.09 (-0.38-0.26)	**0.0248**3	0.19989	**0.00700**	0.16522	FSTL3, C3, CST3, IL6, PLG, FGG, SPARCL1, F5, IGFBP6
Co-stimulation by the CD28 family	Proteomics Serum	-0.61 (-0.77-0.07)	-0.3 (-0.62-0.04)	0.26 (-0.51-0.57)	**0.00501**	0.24333	**0.00246**	**0.02593**	PTPN6, LYN, FYN, SRC, PDPK1, CSK
CD28 co-stimulation	Proteomics Serum	-0.55 (-0.82--0.02)	-0.39 (-0.53--0.05)	0.27 (-0.43-0.59)	**0.00345**	0.15367	**0.00223**	**0.02504**	LYN, FYN, SRC, PDPK1
CD28-dependent PI3K/AKT signalling	Proteomics Serum	-0.59 (-0.77--0.02)	-0.36 (-0.54-0)	0.18 (-0.47-0.62)	**0.00245**	0.14907	**0.00157**	**0.01662**	FYN, PDPK1
DCC-mediated attractive signalling	Proteomics Serum	-0.57 (-0.85--0.02)	-0.37 (-0.47-0.2)	0.14 (-0.43-0.61)	**0.00618**	0.06228	**0.00269**	0.09042	FYN, SRC
IL-3, IL-5 and GM-CSF signalling	Proteomics Serum	-0.25 (-0.45-0.15)	-0.28 (-0.46-0)	0.15 (-0.21-0.39)	**0.00501**	0.56246	**0.03142**	**0.00143**	PTPN6, LYN, FYN, TEC, SHC1, IL3RA, IL5, PRKACA, PTPN11
VEGFR2-mediated cell proliferation	Proteomics Serum	-0.4 (-0.52-0.18)	-0.34 (-0.55-0.14)	0 (-0.38-0.5)	**0.03767**	0.81815	**0.04305**	**0.02438**	SRC, PDPK1, PRKCD, PRKCA, PRKCB, SPHK1, PLCG1
Platelet activation signaling and aggregation	Proteomics Serum	-0.22 (-0.42-0.04)	-0.24 (-0.38--0.1)	0.07 (-0.25-0.35)	**0.00312**	1	**0.00727**	**0.00227**	PTPN6, LYN, FYN, SRC, CFL1, PDPK1, CLEC1B, CSK, PRKCQ
Regulation of IGF transport and uptake by IGFBPs	Proteomics Sputum	0.43 (0.35-0.52)	-0.15 (-0.26--0.03)	-0.2 (-0.44-0.07)	**0.02478**	0.13333	**0.00586**	0.92308	C3, MFGE8, FN1, PAPPA, ALB, GPC3, SPARCL1, F2, FGG, MEP
CD28 co-stimulation	Proteomics	-0.63	0.72	0.17	**0.01824**	0.13333	**0.05568**	**0.02564**	PDPK1, RAC1, FYN, CD86

	Sputum	(-0.72--0.5)	(0.7-0.74)	(-0.47-0.53)					
DCC-mediated attractive signaling	Proteomics Sputum	-0.58 (-0.7--0.41)	0.72 (0.71-0.72)	0.45 (-0.01-0.64)	**0.00941**	0.13333	**0.01758**	**0.02564**	PTK2, NCK1, RAC1, FYN
Extracellular matrix organization	Transcriptomics Bronchial biopsy	-0.31 (-0.36--0.15)	0.21 (-0.1-0.38)	-0.42 (-0.48--0.03)	**0.0184**	**0.01592**	0.28238	**0.03649**	DCN, ITGA6, LAMA4, DDR2, FBLN1, CTSB, LTBP2, DST, LTBP4
Formation of fibrin clots	Transcriptomics Sputum	-0.08 (-0.25--0.05)	-0.53 (-0.54--0.49)	0.07 (-0.05-0.35)	**0.00660**	0.07143	0.15588	**0.00206**	PRCP, A2M, PF4, F3, FGG, F12, GP9, FGA
Extracellular matrix organization	Transcriptomics Sputum	-0.11 (-0.14-0.16)	-0.43 (-0.53--0.4)	0.17 (-0.06-0.35)	**0.00347**	**0.03571**	0.59118	**0.00017**	COL6A6, TNC, CRTAP, COL9A2, JAM2, SDC1, CAPN12, A2M, LAMA2
DAP12 signaling	Transcriptomics Sputum	0.45 (0.38-0.69)	0.74 (0.67-0.97)	-0.3 (-0.66-0.14)	**0.00460**	0.25	**0.04706**	**0.00206**	PIK3CA
Semaphorin interactions	Transcriptomics Sputum	0.24 (0.14-0.3)	-0.37 (-0.51--0.34)	0.21 (-0.09-0.42)	**0.01313**	**0.03571**	0.95294	**0.00327**	ERBB2, SEMA6A, MYH14, PLXNA2, RHOB, MYL12B
Semaphorin 4D in semaphorin signaling	Transcriptomics Sputum	0.51 (0.29-0.54)	-0.42 (-0.52--0.2)	0.19 (-0.16-0.49)	**0.02961**	**0.03571**	0.36177	**0.0258**	ERBB2, MYH14, RHOB, MYL12B
Signaling by NTRK2/TRKB	Transcriptomics Sputum	0.45 (0.38-0.69)	0.74 (0.67-0.97)	-0.3 (-0.66-0.14)	**0.00460**	0.25	**0.04706**	**0.00206**	PIK3CA
Cytokine signaling in immune system	Proteomics Plasma	-0.24 (-0.37-0.11)	-0.21 (-0.34-0.05)	0.19 (-0.24-0.4)	**0.00890**	0.94418	**0.01314**	**0.00533**	TSLP, NFKB2, NOS2, MMP9, MMP1, CCL11, IFNG, IRF5, HSP90
Signaling by interleukins	Proteomics Plasma	-0.25 (-0.46-0.22)	-0.22 (-0.33-0.06)	0.14 (-0.16-0.41)	**0.0038**	0.88862	**0.00886**	**0.0018**3	TSLP, NFKB2, NOS2, MMP9, MMP1, CCL11, IFNG, HSP90B1, ANXA2
Signaling by ERBB2 in cancer	Proteomics Serum	-0.16 (-0.32-0.07)	0.08 (-0.14-0.26)	-0.09 (-0.29-0.17)	0.05125	**0.02194**	0.74612	**0.049392**	SHC1, ERBB4, EGFR, ERBB2
Cytokine signaling in immune system	Proteomics Serum	-0.16 (-0.26-0.11)	-0.18 (-0.3--0.01)	0.12 (-0.2-0.25)	**0.04019**	0.66268	0.08144	**0.01608**	CFL1, UBE2N, SHC1, PTPN11, CSK, PPIA, PRKACA, EIF4G2, RPS6KA3
Signaling by SCF KIT	Proteomics Serum	-0.41 (-0.56-0.02)	-0.26 (-0.45-0.08)	0.07 (-0.33-0.5)	**0.00968**	0.35257	**0.00620**	**0.02438**	RAC1, PTPN11, TEC, PTPN6, FER, LYN, PRKCA, SRC, JAK2, LCK
Degradation of the extracellular matrix	Proteomics Serum	-0.17 (-0.31-0.05)	-0.02 (-0.2-0.13)	0.08 (-0.19-0.3)	**0.01557**	0.10863	**0.00596**	0.13233	COL23A1, MMP2, BSG, CASP3, MMP12, KLK7, MMP17, ADAMTS5
Extracellular matrix organization	Proteomics Serum	-0.11 (-0.29-0.08)	-0.08 (-0.25-0.09)	0.05 (-0.17-0.27)	**0.03046**	0.61723	**0.02108**	**0.03716**	COL23A1, DMP1, PPIB, PRKCA, ICAM1, MMP2, JAM3, BSG, CAS

Signaling by NTRKs	Proteomics Serum	-0.32 (-0.42-0.24)	-0.36 (-0.52--0.12)	0.09 (-0.2-0.41)	**0.00109**	0.26896	**0.03042**	**0.00019**	RAC1, SHC1, PTPN11, RPS6KA3, ADCYAP1, CRK, MAPK1, DUSP3
DAP12 signaling	Proteomics Serum	-0.34 (-0.65-0.18)	-0.47 (-0.68--0.22)	0.28 (-0.39-0.6)	**0.00131**	0.35257	**0.01193**	**0.00053**	RAC1, SHC1, BTK
Semaphorin interactions	Proteomics Serum	-0.28 (-0.65-0.17)	-0.54 (-0.67--0.2)	0.27 (-0.22-0.53)	**0.00084**	0.24218	**0.01539**	**0.00026**	CFL1, RAC1
Semaphorin 3A PAK-dependent axon repulsion	Proteomics Serum	-0.28 (-0.65-0.17)	-0.54 (-0.67--0.2)	0.27 (-0.22-0.53)	**0.00084**	0.24218	**0.01539**	**0.00026**	CFL1, RAC1
Signaling by interleukins	Proteomics Serum	-0.05 (-0.2-0.05)	-0.18 (-0.26--0.03)	0.18 (-0.24-0.23)	**0.04041**	0.11225	0.29458	**0.01608**	CFL1, UBE2N, SHC1, PTPN11, PPIA, PRKACA, RPS6KA3, TEC
Signaling by NTRK2/TRKB	Proteomics Serum	-0.21 (-0.62-0.15)	-0.47 (-0.68--0.29)	0.17 (-0.13-0.61)	**0.00106**	0.11975	**0.03245**	**3e-04**	RAC1, SHC1, PTPN11
Intracellular signaling by second messangers	Proteomics Serum	-0.25 (-0.34-0.06)	-0.2 (-0.27--0.1)	0.16 (-0.22-0.29)	**0.03923**	0.6512	**0.03803**	**0.03020**	PDPK1, RAC1, PTPN11, AREG, PRKACA, ERBB4, CAMK2B, CAMK2
Cytokine signaling in immune system	Proteomics Sputum	0.1 (-0.18-0.41)	0.37 (0.27-0.46)	-0.42 (-0.49--0.26)	**0.02206**	0.8	**0.03956**	**0.02564**	CTSG, TNFRSF8, CD36, CCL2, FCGR1A, CSF3R, IL1RL1, TNFSF
Extracellular matrix organization	Proteomics Sputum	0.08 (-0.1-0.28)	0.5 (0.45-0.55)	-0.38 (-0.43--0.24)	**0.03091**	0.26667	0.05568	0.05128	CTSG, SERPINE1, THBS1, CTSB, TGFB1, PECAM1, HAPLN1, BGN
Signaling by interleukins	Proteomics Sputum	0.28 (0.03-0.45)	0.23 (0.12-0.35)	-0.36 (-0.44--0.31)	**0.013931**	1	**0.01393**	**0.02564**	CTSG, CD36, CCL2, CSF3R, IL1RL1, IL16, TGFB1, IL15RA

Data are presented as median (interquartile range). Between-group comparisons were performed using the Kruskal-Wallis test with Dunn's multiple comparison test. Bold indicates significant p values (<0.05).

Datasets: Blood transcriptomics: RAC1, n=25; RAC2, n=28; RAC3, n=30. Bronchial biopsy transcriptomics: RAC1, n=8; RAC2, n=13; RAC3, n=6. Bronchial brushing transcriptomics: RAC1, n=13; RAC2, n=14; RAC3, n=8. Sputum transcriptomics: RAC1, n=3; RAC2, n=5; RAC3, n=14. Plasma proteomics: RAC1, n=29; RAC2, n=33; RAC3, n=36. Serum proteomics: RAC1, n=27; RAC2, n=30; RAC3, n=41. Sputum proteomics: RAC1, n=4; RAC2, n=2; RAC3, n=11.

See abbreviation list for abbreviations.

Supplementary Table S17. Differential expression of serum proteomic molecular signatures across the severe asthma radiomultiomic-associated clusters (RACs)

**Signature names**	**Proteins in serum**
Complement cascade RAC1 vs RAC2	CFH, CFI, C5, C9, C1S, CFD
GPVI-mediated activation cascade RAC1 vs RAC3	PTPN6, LYN, FYN, PDPK1, CLEC1B, GP6
Signaling by ERBB2 RAC1 vs RAC3	FYN, SRC, PRKCD, PRKCA, SHC1, ERBB4, YES1, PLCG1, NRG1
Signaling by SCF KIT RAC1 vs RAC3	PTPN6, LYN, FYN, SRC, FER, TEC, RAC1, PRKCA, PTPN11, JAK2
Regulation of KIT signaling RAC1 vs RAC3	PTPN6, LYN, FYN, SRC
Complement cascade RAC1 vs RAC3	CFI, C9, CFH, C5, CD55, C3, FCN1, CRP
Regulation of IGF transport and uptake by IGFBPs RAC1 vs RAC3	FSTL3, C3, CST3, IL6, PLG, FGG, SPARCL1, F5, IGFBP6
Co-stimulation by the CD28 family RAC1 vs RAC3	PTPN6, LYN, FYN, SRC, PDPK1, CSK
CD28 co-stimulation RAC1 vs RAC3	LYN, FYN, SRC, PDPK1
CD28-dependent PI3K/AKT signaling RAC1 vs RAC3	FYN, PDPK1
DCC-mediated attractive signaling RAC1 vs RAC3	FYN, SRC
Interleukin-3, interleukin-5 and GM-CSF signaling RAC1 vs RAC3	PTPN6, LYN, FYN, TEC, SHC1, IL3RA, IL5, PRKACA, PTPN11
VEGFR2-mediated cell proliferation RAC1 vs RAC3	SRC, PDPK1, PRKCD, PRKCA, PRKCB, SPHK1, PLCG1
Platelet activation signaling and aggregation RAC1 vs RAC3	PTPN6, LYN, FYN, SRC, CFL1, PDPK1, CLEC1B, CSK, PRKCQ
Signaling by ERBB2 in cancer RAC2 vs RAC3	SHC1, ERBB4, EGFR, ERBB2
Cytokine signaling in immune system RAC2 vs RAC3	CFL1, UBE2N, SHC1, PTPN11, CSK, PPIA, PRKACA, EIF4G2, RPS6KA3
Signaling by SCF KIT RAC2 vs RAC3	RAC1, PTPN11, TEC, PTPN6, FER, LYN, PRKCA, SRC, JAK2, LCK3
Extracellular matrix organization RAC2 vs RAC3	COL23A1, DMP1, PPIB, PRKCA, ICAM1, MMP2, JAM3, BSG, CAS
Signaling by NTRK RAC2 vs RAC3	RAC1, SHC1, PTPN11, RPS6KA3, ADCYAP1, CRK, MAPK1, DUSP3
DAP12 signaling RAC2 vs RAC3	RAC1, SHC1, BTK
Semaphorin interactions RAC2 vs RAC3	CFL1, RAC1
Semaphorin 3A PAK-dependent axon repulsion RAC2 vs RAC3	CFL1, RAC1
Signaling by interleukins RAC2 vs RAC3	CFL1, UBE2N, SHC1, PTPN11, PPIA, PRKACA, RPS6KA3, TEC
Signaling by NTRK2/TRKB RAC2 vs RAC3	RAC1, SHC1, PTPN11
Intracellular signaling by second messengers RAC2 vs RAC3	PDPK1, RAC1, PTPN11, AREG, PRKACA, ERBB4, CAMK2B, CAMK2
