# Supplementary material for: Radiomultiomics: quantitative CT clusters of severe asthma associated with multiomics
Source: Eur Respir J. 2024 Nov 21;64(5):2400207. doi: 10.1183/13993003.00207-2024 (PMC11579543; doi:10.1183/13993003.00207-2024)
Supplement: Supplementary file 5 [file ERJ-00207-2024.Shareable.pdf]

Radiomultiomics: quantitative CT clusters of severe asthma associated with multiomics

Nazanin Zounemat Kermani, Kian Fan Chung, Giuseppe Macis, Giuseppe Santini, Franz A.A. Clemeno, Ali Versi, Kai Sun, Mahmoud I. Abdel-Aziz, Lars I. Andersson, Charles Auffray, Yusef Badi, Per Bakke, Chris Brightling, Paul Brinkman, Massimo Caruso, Pascal Chanez, Bertrand De Meulder, Ratko Djukanovic, Leonardo Fabbri, Stephen J. Fowler, Ildiko Horvath, Peter Howarth, Anna J. James, Johan Kolmert, Monica Kraft, Chuan-Xing Li, Anke H. Maitland-van der Zee, Mario Malerba, Alberto Papi, Klaus Rabe, Marek Sanak, Dominick E. Shaw, Dave Singh, Maria Sparreman Mikus, Maarten van Den Berge, Asa M. Wheelock, Craig E. Wheelock, Valentyna Yasinska, Yi-ke Guo, Scott Wagers, Peter J. Barnes, Andrew Bush, Peter J. Sterk, Sven-Erik Dahlen, Ian M. Adcock, Salman Siddiqui and Paolo Montuschi on behalf of the U-BIOPRED and ATLANTIS Study Groups

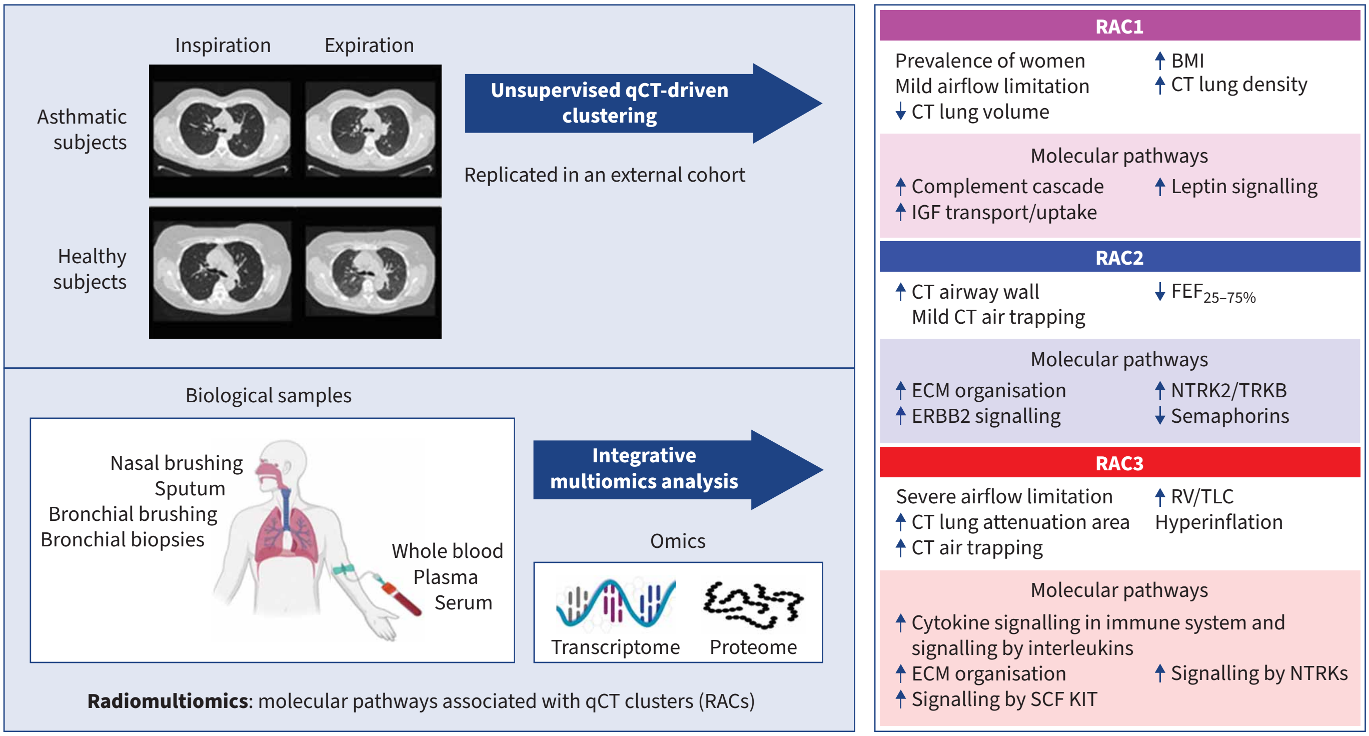

GRAPHICAL ABSTRACT Overview of the study. (q)CT: (quantitative) computed tomography; RAC: radiomultiomic-associated cluster; BMI: body mass index; IGF: insulin-like growth factor; $FEF_{25–75\%}$: forced expiratory flow at 25–75% of forced vital capacity; ECM: extracellular matrix; RV: residual volume; TLC: total lung capacity. See the supplementary material for a full list of definitions of abbreviations of genes, proteins and pathways.

SHAREABLE PDF

Radiomultiomics: quantitative CT clusters of severe asthma associated with multiomics

Nazanin Zounemat Kermani[1], Kian Fan Chung[2,3], Giuseppe Macis[4], Giuseppe Santini[5], Franz A.A. Clemeno[6], Ali Versi[1], Kai Sun[1], Mahmoud I. Abdel-Aziz[7], Lars I. Andersson[8,9], Charles Auffray[10], Yusef Badi[1], Per Bakke[11], Chris Brightling[12], Paul Brinkman[7], Massimo Caruso[13], Pascal Chanez[14], Bertrand De Meulder[10], Ratko Djukanovic[15], Leonardo Fabbri[16], Stephen J. Fowler[17], Ildiko Horvath[18], Peter Howarth[15], Anna J. James[19], Johan Kolmert[20], Monica Kraft[21], Chuan-Xing Li[22], Anke H. Maitland-van der Zee[7], Mario Malerba[23], Alberto Papi[17], Klaus Rabe[24], Marek Sanak[25], Dominick E. Shaw[26], Dave Singh[27], Maria Sparreman Mikus[8], Maarten van Den Berge[28], Asa M. Wheelock[22], Craig E. Wheelock[20], Valentyna Yasinska[8,9], Yi-ke Guo[1], Scott Wagers[29], Peter J. Barnes[2], Andrew Bush[2], Peter J. Sterk[7], Sven-Erik Dahlen[8,9,20], Ian M. Adcock[2,30], Salman Siddiqui[2,30] and Paolo Montuschi[2,5,30] on behalf of the U-BIOPRED and ATLANTIS Study Groups

[1]Data Science Institute, Imperial College London, London, UK. [2]National Heart and Lung Institute, Imperial College London, London, UK. [3]Royal Brompton and Harefield Hospitals, London, UK. [4]Radiology, Faculty of Medicine, Catholic University of the Sacred Heart, Rome, Italy. [5]Pharmacology, Faculty of Medicine, Catholic University of the Sacred Heart, Rome, Italy. [6]Department of Respiratory Sciences, University of Leicester, Leicester, UK. [7]Department of Pulmonology, Amsterdam UMC, University of Amsterdam, Amsterdam, The Netherlands. [8]Department of Medicine Huddinge, Karolinska Institutet, Stockholm, Sweden. [9]Department of Respiratory Medicine, Karolinska University Hospital, Stockholm, Sweden. [10]European Institute for Systems Biology and Medicine, CNRS-ENS-UCBL-INSERM, Université de Lyon, Lyon, France. [11]Department of Clinical Science, University of Bergen, Bergen, Norway. [12]Institute for Lung Health, NIHR Leicester Biomedical Research Centre, Department of Respiratory Sciences, University of Leicester, Leicester, UK. [13]Department of Biomedical and Biotechnological Sciences, University of Catania, Catania, Italy. [14]AP-HM – Clinique des Bronches, Allergies et Sommeil, Aix Marseille Université, Marseille, France. [15]NIHR Southampton Respiratory Biomedical Research Unit and Clinical and Experimental Sciences, University of Southampton, Southampton, UK. [16]Respiratory Medicine, Department of Translational Medicine, University of Ferrara, Ferrara, Italy. [17]Division of Infection, Immunity and Respiratory Medicine, School of Biological Sciences, University of Manchester, Manchester University NHS Foundation Trust, Manchester Academic Health Science Centre, Manchester, UK. [18]Semmelweis University, Budapest, Hungary. [19]Department of Women's and Children's Health, Karolinska Institutet, Stockholm, Sweden. [20]Institute of Environmental Medicine, Centre for Allergy Research, Karolinska Institutet, Stockholm, Sweden. [21]Samuel Bronfman Department of Medicine, Icahn School of Medicine at Mount Sinai Hospital, New York, NY, USA. [22]Department of Medicine Solna, Karolinska Institutet, Stockholm, Sweden. [23]Department of Translational Medicine, University of Piemonte Orientale, Novara, Italy. [24]LungenClinic Grosshansdorf and Department of Medicine, Christian Albrechts University, Airway Research Center North within the German Center for Lung Research (DZL), Kiel, Germany. [25]Division of Clinical Genetics and Molecular Biology, Department of Medicine, Jagiellonian University Medical College, Kraków, Poland. [26]Respiratory Research Unit, University of Nottingham, Nottingham, UK. [27]Centre for Respiratory Medicine and Allergy, Manchester University NHS Foundation Hospital Trust, University of Manchester, Manchester, UK. [28]Department of Pulmonology, University of Groningen, University Medical Center Groningen, Groningen, The Netherlands. [29]Biosci Consulting, Maasmechelen, Belgium. [30]I.M. Adcock, S. Siddiqui and P. Montuschi contributed equally to this article as lead authors and supervised the work.

Corresponding author: Paolo Montuschi (paolo.montuschi@unicatt.it; p.montuschi@imperial.ac.uk)

Check for updates

Shareable abstract (@ERSpublications)
Severe asthma qCT clusters are replicable and associated with selective molecular pathways. By clarifying the potential pathogenetic mechanisms underlying its heterogeneity, radiomultiomics might improve severe asthma personalised medicine. https://bit.ly/3WfySPI

Cite this article as: Zounemat Kermani N, Chung KF, Macis G, *et al*. Radiomultiomics: quantitative CT clusters of severe asthma associated with multiomics. *Eur Respir J* 2024; 64: 2400207 [DOI: 10.1183/13993003.00207-2024].

This extracted version can be shared freely online.

Copyright ©The authors 2024.

Abstract

Background Lung quantitative computed tomography (qCT) severe asthma clusters have been reported, but their replication and underlying disease mechanisms are unknown. We identified and replicated qCT clusters

This version is distributed under the terms of the Creative Commons Attribution Licence 4.0.

This article has an editorial commentary: https://doi.org/10.1183/13993003.01639-2024

Received: 1 Feb 2024
Accepted: 21 July 2024

of severe asthma in two independent asthma cohorts and determined their association with molecular pathways, using radiomultiomics, integrating qCT, multiomics and machine learning/artificial intelligence.

Methods We used consensus clustering on qCT measurements of airway and lung CT scans, performed in 105 severe asthmatic adults from the U-BIOPRED cohort. The same qCT measurements were used to replicate qCT clusters in a subsample of the ATLANTIS asthma cohort (n=97). We performed integrated enrichment analysis using blood, sputum, bronchial biopsies, bronchial brushings and nasal brushings transcriptomics and blood and sputum proteomics to characterise radiomultiomic-associated clusters (RACs).

Results qCT clusters and clinical features in U-BIOPRED were replicated in the matched ATLANTIS cohort. In the U-BIOPRED cohort, RAC1 (n=30) was predominantly female with elevated body mass index, mild airflow limitation, decreased CT lung volume and increased lung density and upregulation of the complement pathway. RAC2 (n=34) subjects had airway wall thickness and a mild degree of airflow limitation, with upregulation of proliferative pathways including neurotrophic receptor tyrosine kinase 2/tyrosine kinase receptor B, and downregulation of semaphorin pathways. RAC3 (n=41) showed increased lung attenuation area and air trapping, severe airflow limitation, hyperinflation, and upregulation of cytokine signalling and signalling by interleukin pathways, and matrix metallopeptidase 1, 2 and 9.

Conclusions U-BIOPRED severe asthma qCT clusters were replicated in a matched independent asthmatic cohort and associated with specific molecular pathways. Radiomultiomics might represent a novel strategy to identify new molecular pathways in asthma pathobiology.
